# A Global Synthesis of the Correspondence Between Epizoic Barnacles and Their Sea Turtle Hosts

**DOI:** 10.1093/iob/obab002

**Published:** 2021-02-05

**Authors:** John D Zardus

**Affiliations:** Department of Biology, The Citadel, 171 Moultrie Street, Charleston, SC 29409, USA

## Abstract

Barnacles that are obligate epizoites of sea turtles are not parasites in the traditional sense. However, they can impair their hosts in some instances, disqualifying the association as strictly commensal. Characterizing these interactions requires knowing which epibionts pair with which hosts, but records of barnacles from sea turtles are scattered and symbiont/host match-ups remain equivocal. The objective of this study was to collate global records on the occurrence of barnacles with sea turtles and describe each species pair quantitatively. Records reporting barnacles with sea turtles were searched spanning the last 167 years, including grey literature, and findings were enumerated for 30,580 individual turtles to evaluate prevalence. The data were summarized globally as well as subdivided across six geographic regions to assess constancy of the affiliations. Patterns of partnering were visualized by hierarchical clustering analysis of percent occurrence values for each barnacle/turtle pair and the relative selectivity of each symbiont and susceptibility of each host were evaluated. After adjusting for synonymies and taxonomic inaccuracies, the occurrence of 16 nominal species of barnacles was recorded from all 7 extant sea turtle species. Mostly, barnacles were not specific to single turtle species, partnering on average with three hosts each. Neither were barnacles entirely host-consistent among regions. Three barnacles were common to all sea turtles except leatherbacks. The most common, widespread, and least selective barnacle was *Chelonibia testudinaria*, the only symbiont of all turtles. Excluding single-record occurrences, the barnacle *Stomatolepas transversa* was the only single-host associate of any hard-shell sea turtle (the green sea turtle) and *Platylepas coriacea* and *Stomatolepas dermochelys* were exclusive associates of leatherback sea turtles. Green sea turtles were the most vulnerable to epibiosis, hosting 13 barnacle species and Kemp’s ridley sea turtles were the least, hosting three. Geographically, there was an average of nine barnacle species per world region, with diversity highest in the Pacific Ocean (12 species) and lowest in the Mediterranean Sea (6 species). It is paradoxical that the flexibility of barnacles for multiple host species contrasts with their overall strict specificity for sea turtles, with each symbiont occupying a virtually unique suite of turtle hosts.

Barnacles, to the undiscerning eye, are as boring as rivets. This is largely attributable to the erroneous impression that they don’t go anywhere and don’t do anything, ever. The truth of the matter is that they don’t go anywhere and don’t do anything merely sometimes—and that, other times, barnacle life is punctuated with adventurous travel, phantasmagorical transformations, valiant struggles, fateful decisions, and eating.

David Quammen, Point of Attachment, 1998

## Introduction

Barnacles are the epitome of a sessile animal, so it seems incongruous that some are among the widest-roving invertebrates on the planet, maybe beyond even what Mr. Quammen has conceived. All members of the barnacle superfamily Coronuloidea have acquired the borrowed ability to travel many hundreds, or thousands of kilometers over a lifetime as epibionts of their mobile hosts or basibionts. These epizoites of sea turtles, sea snakes, crabs, whales, and other mobile fauna (Darwin 1854; Zann 1975; Hayashi 2013a), utilize their live substratum as a platform for feeding and dispersal, but not as a source of nutrition. The greatest number specializes on sea turtles but their host repertoires and specificity for particular turtle species have not previously been rigorously evaluated. I endeavor to fill this gap to gain greater understanding of these associations and insight on how barnacles select a mobile home.

The nature of barnacle–turtle relationships falls somewhere between parasitism and mutualism. Not a phoretic symbiosis, whereby one animal attaches to another temporarily for conveyance (White et al. 2017), the association is most frequently considered a commensalism. The term refers to feeding at a common table (van Beneden 1876) and results in the commensal deriving benefit from the arrangement while the host remains unaffected. However, the view that turtle hosts are not negatively affected by barnacles is, depending on the situation and the symbiont involved, often plausible, in some cases debatable, and occasionally untenable. Commensalisms are rarely demonstrated as obligate and specific (Wahl and Mark 1999) and many are not stable, tipping toward the benefit or detriment of the host depending on circumstances; indeed, the argument has been made that the concept of commensalism, in the narrow sense, is a theoretical state that cannot be empirically demonstrated (Zapalski 2011). Thus, multiple states apply for epizoic barnacles.

**Fig. 1:**
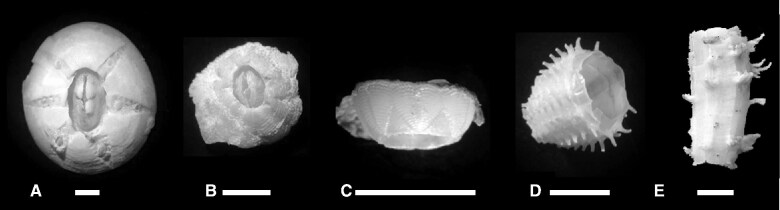
Barnacle exemplars that attach to sea turtles by: (**A**) cementing, *Chelonibia testudinaria*; (**B**) clinging, *Platyleaps hexastylos*; (**C**); embedding, *Stomatolepas elegans* [the shell is right side up!]; (**D**) penetrating, *Stephanolepas muricata*; and (**E**) boring, *Chelolepas cheloniae* (scale bars = 5 mm).

Whatever the designation, advantages certainly accrue to barnacles by living on mobile hosts, including access to reliable currents for passive feeding and protection from benthic predators (Foster 1987). The obligate nature of coronuloid barnacles on their hosts, especially as associates of whales and turtles (Scarff 1986; Hayashi 2013a), is well-demonstrated, with several taxa known from only a single species of cetacean or chelonian (Marloth 1900; Monroe and Limpus 1979). Of the more than 200 species of epibionts documented from sea turtles (Frick and Pfaller 2013), most are facultative associates, but barnacles are the most common obligate taxa. Yet, despite their affinity for sea turtles, a one barnacle–one turtle paradigm is likely the exception rather than the rule. For example, the common “turtle” barnacle, *Chelonibia testudinaria* (Fig. 1A), has the most elastic host use, regularly utilizing manatees, various crabs, and all species of sea turtles as basibionts (Zardus et al. 2014) and exceptionally, other kinds of reptiles and crustaceans (Ross and Jackson 1972; Badrudeen 2000; Ortiz et al. 2004; Nifong and Frick 2011), even various synthetic substrata (Edmondson and Ingram 1939; Frazier and Margaritoulis 1990; Sloan et al. 2014). Lacking an all-encompassing label to describe the relationship of barnacles with sea turtles, it is perhaps most easily summarized as an obligate association of generally intermediate specificity with neutral to negative consequences for the host.

No reciprocal benefit to sea turtles has been demonstrated by the attachment of barnacles, though the idea that they may provide disruptive camouflage has been suggested (Wahl and Mark 1999; Kobayashi 2000). With most barnacle species, the partnership is benign for turtles under typical conditions, depending primarily on attachment mode. Some species of barnacle cement to or bore into turtle hard parts such as shell and scales, others latch on to leathery epidermis or embed in the supple skin of the neck and limbs. For example, the skin-clinging barnacle *Platylepas hexastylos* (Fig. 1B) loosely clamps to the outer layers of turtle epidermis but causes no apparent damage or disadvantage (Flint et al. 2009). The superficially-embedding *Stomatolepas elegans* (syn. *S. praegustator*) (Fig. 1C) presents a similar but curious case, causing no obvious wounding or irritation as it presses itself, without penetration, into the epidermis, even when densely inhabiting the tongue and gullet (Pilsbry 1910). However, when turtles are injured or debilitated in some way, they may acquire an unusually heavy load of barnacles (Herbert and Jacobson 1995) which reduces their swimming efficiency, especially for sub-adult turtles. Barnacles may also arbitrarily overgrow the eyes or settle in wounds and exacerbate shell fissuring. A few species of barnacles are unequivocally and intrinsically harmful to sea turtles to varying degrees, but not in a parasitic sense. The barnacle *Stephanolepas muricata* (Fig. 1D) penetrates the epidermis of several species of sea turtles (Frick et al. 2011), mostly along the leading edges of flippers, causing pitting, lacerations, and bleeding. The most pernicious is *Chelolepas cheloniae* (*Tubicinella cheloniae* of some sources) (Fig. 1E) which bores into both epidermis and carapace (Flint et al. 2009), occasionally penetrating to the bone or body cavity, leading to infection and sometimes death of the host (Herbert and Jacobson 1995).

The evolutionary history of the association of barnacles with sea turtles is ancient but not fully resolved. Though both host and symbiont leave fossil remains, barnacle shell plates typically disarticulate at death and their remnants are scarce (Collareta et al. 2019). Fossil evidence indicates the lineage of extant sea turtles, Superfamily Chelonioidea, has its genesis around 130* *mya in the Early Cretaceous (Evers and Benson 2019). The leatherback sea turtle, *Dermochelys coriacea*, is the oldest living taxon, likely originating in the Eocene (Cadena and Parham 2015). Of the hardshell species, green and hawksbill sea turtles (*Chelonia mydas* and *Eretmochelys imbricata*, respectively) are next oldest, appearing in the Miocene (perhaps along with the soft-carapaced flatback sea turtle, *Natator depressus*, as well). They are followed by the loggerhead sea turtle, *Caretta caretta*, in the Pliocene and lastly the two species of ridley sea turtles, the olive ridley, *Lepidochelys olivacea* and Kemp’s ridley, *Lepidochelys kempii* (Bowen and Karl 2007; Cadena and Parham 2015). The oldest fossil coronuloid barnacle is an Eocene chelonibiid dating to 34–38* *mya and sharing structural features with recent *Chelonibia* species (Ross and Newman 1967). Although, turtle-fouling *per se* is argued not to have originated until 20–23* *mya with the extinct genus *Protochelonibia* (Harzhauser et al. 2011). Epizoism in coronuloid barnacles may have commenced with molluscan or crab hosts (Ross and Newman 1967) before transferring to turtles and later whales, though no fossil evidence is known. A counter view is offered by a fossil-calibrated phylogeny (Hayashi et al., 2013) which puts coronuloid origins in the Early Eocene, inferring a mid-Eocene appearance of the clade leading to the skin-attaching barnacles, *Stomatolepas*, and *Platylepas*, pre-dating by ∼10 million years the shell-cementing *Chelonibia* clade.

While adult barnacles are largely sessile, they have microscopic free-swimming larvae which, in the case of epizoic species, enable them to reach mobile hosts (Moyse 1961; Molenock and Gomez 1972; Zardus and Hadfield 2004; Nogata and Matsumura 2006; Liu et al. 2016). Barnacles typically reproduce through direct copulation and disperse via a swimming larval phase that disseminates widely in the plankton (Anderson 1994). Following a week or more of planktonic development, passing through seven larval stages, these crustaceans attach to a suitable host only at the final cyprid stage. More than a dozen species of barnacles select their hosts from a smorgasbord of seven extant sea turtle species that vary in habitat use and geographic distribution. Sea turtles mostly inhabit tropical and subtropical regions seeking food variously as herbivores, omnivores, and specialists of sponges and jellyfish, roaming as restricted coastal foragers to open ocean nomads (Spotila 2004). In addition to finding an acceptable basibiont, the barnacles must also target a specific attachment location on the host (Robinson et al. 2019).

For barnacles of rocky shores, it is possible to explain patterns of population connectivity with oceanographic processes (Chan et al. 2012; Wares 2020). But linkage dynamics for species whose dispersal is also influenced by host movements presents a challenge to characterizing biogeographic boundaries. Intuitively, the phylogeography of any hitchhiking associate of a mobile host should follow its hosts’ distribution. But contrary to this expectation, some epibionts exhibit genotypic distributions that differ substantially from their hosts’. For instance, a cosmopolitan leech, parasitic with multiple sea turtle species shows, surprisingly, no genetic variation between Atlantic and Pacific oceans like its hosts (Tseng et al. 2018). And whale lice, amphipod crustaceans which dine on sloughing skin, complete their entire life cycle on a single cetacean (Balbuena and Raga 1991) but exchange genes extensively beyond individual host platforms and boundaries of whale subpopulations (Kaliszewska et al. 2005).

Although living on mobile hosts certainly benefits dispersal of epizoites, it creates the countermanding challenge of needing to attach to itinerant substrata that vary in material properties and that are highly limited due to rarity. Sea-turtle geospatial distributions, though incompletely characterized on a fine scale, are coarsely known and five of the seven species are circumglobal in tropical and subtropical waters. The exceptions are Kemp’s ridley and flatback sea turtles which are both regionally restricted, the former nesting only in the Gulf of Mexico and wandering into the western Atlantic and the latter inhabiting the islands of the Torres Strait and the northern coast of Australia from the Indian to the Pacific Ocean (Wallace et al. 2010). Where turtles and barnacle larvae meet remains inconclusive but the feeding or nesting localities of adult turtles offer a clue that it happens coastally for most species (Sloan et al. 2014). The particular abilities of barnacles to find and colonize sea turtles may lie in life history strategies that synchronize larval production with host movements and a chemosensitive and highly versatile cyprid attachment organ (Al-Yahya et al. 2016; Dreyer et al. 2020).

That barnacles associate with sea turtles and other mobile megafauna has undoubtedly been recognized since antiquity (Blick et al. 2010), but historical documentation is known only from several hundred years ago (Hayashi 2014). The first edition of *Systema Naturae*, (Linnaeus 1758) enters green and loggerhead sea turtles into the scientific record along with one of their most common barnacles, *C. testudinaria* (as *Lepas testudinaria*). However, taxonomic identities among all extant sea turtles were only stabilized in the latter half of the 20th century through detailed survey work (Carr and Caldwell 1956; Hughes et al. 1967) and phylogenetic study (Bowen et al. 1993). Foundational literature on cirripedes over the last ≥150 years (Darwin 1854; Gruvel 1905; Pilsbry 1916; Newman and Ross 1976) set the stage for contemporary systematics of barnacles (Pérez-Losada et al. 2004; 2014), but the modern era of understanding the diversity of barnacles associated with sea turtles dawned with Monroe and Limpus (1979). To date, correspondence between the known barnacle species and their sea turtle hosts remains ill-defined, scattered among taxonomic lists of barnacles and survey records of sea turtles, hampered by unrecorded or imprecisely identified hosts in the former and misidentified or unidentified symbionts in the latter. Without a detailed compilation of global records, it cannot be stated with certainty which barnacle species affiliate with which turtle species, how specific their associations are, and whether patterns differ geographically. This hinders both our understanding of host selectivity by barnacles and knowledge of epibiont susceptibility among sea turtle.

The primary objectives of this study were to catalog the association of obligate, epizoic barnacles with sea turtles and assess their degree of specificity. Global records enumerating the numbers of turtles by species hosting each kind of barnacle were searched to collate and quantitatively evaluate the incidence of pairing between each host and symbiont and gauge the relative prevalence of association. The same comparisons were also made within geographic subdivisions to test the fidelity of barnacle/turtle match-ups globally. Breadth in host utilization was predicted to vary by symbiont, so a barnacle selectivity index was generated, evaluating the number of host species exploited by each taxon. Similarly, a measure of susceptibility to epibiosis for each turtle species was also formulated from barnacle occurrence rates.

## Methods

A heterogeneous assemblage of records documenting associations between epizoic coronuloid barnacles and sea turtles was searched from Darwin (1854), the beginning of stable barnacle taxonomies, to the present. Sources fell into three broad categories: (1) “peer-reviewed publications” comprising published journal articles, edited monographs, and books; (2) “technical reports,” consisting of government and private reports, theses, and conference proceedings; and (3) “collections,” encompassing specimens in museum and private collections. No attempt was made to compile information on species of epizoic stalked barnacles (Lepadomorpha) or opportunistic acorn barnacles. Particular attention was paid to locating records enumerating the number of turtles with and without accompanying barnacles. Accounts were disregarded that did not mention barnacles or identify them to a useful degree. A catalog was generated from the records, enumerating the sea turtles examined by species and the proportion hosting each species of barnacle (Appendix I). The data were summed and tabulated, first for global metrics then again for geographic comparisons, subdividing the world into six regions: (1) Atlantic Ocean, (2) Caribbean Sea and Gulf of Mexico, (3) Mediterranean Sea, (4) Indian Ocean, (5) Pacific Ocean, and (6) Central Indo-Pacific (defined herein as those seas and straits from the northern coast of Australia in the south to the shores of Southeast Asia bordering the South China Sea in the north, and from the east side of Sumatra across the Indonesian archipelago to the Philippine Islands in the west).

For purposes of calculating percent occurrence, a value of zero was applied to turtles for each barnacle species specifically stated as absent or not mentioned but known to occur with that host in the region. In several instances where barnacle species were not reported they could be inferred from descriptions or photographs. In surveys, where “most” or a “majority” of the turtles were listed as having barnacles, a proportion of 75% was used as an estimate. Misidentifications of host turtles and more commonly the barnacles, especially in early records, were corrected where known with the latest information on synonymies and annotated accordingly in the catalog. For example, recent work confirms that leatherback sea turtles associate with only a few specific barnacles (Robinson et al. 2017b) and for any accounts mentioning *P. hexastylos* or *S. elegans* with leatherbacks, the species *P. coriacea* and *S. dermochelys* were substituted, respectively. The single report of the sea snake barnacle *P. indicus* from a leatherback (Fernando 2006) is probably a misidentification of *P. coriacea* and was treated as such. In some records, locations on the turtle body where barnacles attached were noted, providing useful qualitative information, but such reporting was too infrequent for quantitative comparisons. Likewise, barnacle counts or densities per turtle were not analyzed due to a paucity of information.

Patterns of association between barnacles and sea turtles were visualized by charting hierarchical clustering for each barnacle/turtle pair using the unweighted pair group method with arithmetic mean (UPGMA) of Euclidean distances of the global occurrence rates. From the tabulated data, two indices of association were calculated: first a measure of host selectivity for each barnacle species (*S_b_*), ranging from 10 (most selective) to 0 (least selective), and second a value estimating the susceptibility of each turtle species to barnacle epibiosis (*S_t_*) ranging from 10 (most susceptible) to 0 (least susceptible) using quotients of barnacle and turtle species as follows: where “*T*” represents the number of sea turtle species found hosting barnacle species “x,” “*N*_t_” represents the total number of sea turtle species globally (i.e., 7), “*B*” represents the number of barnacle species hosted by turtle species “x,” and “*N*_b_” represents the total number of named barnacle species globally (i.e., 16).

1. barnacle selectivity index$S_{b}=(1-\frac{T_{x}}{N_{t}})$ · 10
2. turtle susceptibility index$S_{t}=\frac{B_{x}}{N_{b}}$ · 10,

## Results

**Fig. 2:**
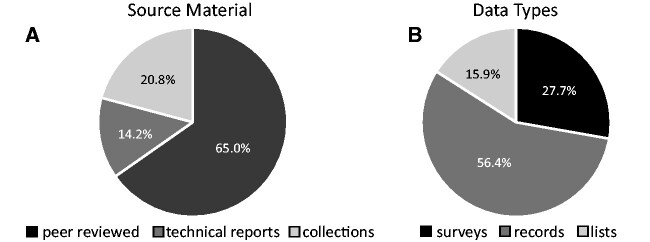
Proportion of 289 searched records by category (**A**) and by type of data resulting (**B**).

Searching peer-reviewed publications, technical reports, and collections from the past 167 years, I found 289 records reporting sea turtles hosting named coronuloid barnacles (Fig. 2A). A complete list of sources annotated with counts and explanations for debatable species determinations is provided in Supplementary Material (Appendix I). The data were spread among three categories: “surveys,” tallies of targeted sea turtle populations mentioning the presence or absence of barnacles by species and sometimes their abundance; “records,” studies of sea turtles mentioning chance reports of barnacles; and “lists,” museum or regional taxonomic inventories identifying barnacles and host species (Fig. 2B). In total, percent occurrences of turtles with barnacles were tallied from 30,580 individually observed sea turtles, covering all seven extant species, with 16 nominal species of barnacles identified as obligate associates (Table 1).

**Table 1:** Global correspondence of epibiont/basibont associations between the seven sea-turtle species of the world and the 19 coronuloid barnacle taxa (16 named species) obligate with them. Values presented are percentages of turtles hosting each epibiont apportioned by turtle species: green sea turtle, *Chelonia mydas* (C.m.); hawksbill sea turtle, *Eretmochelys imbricata*, (E.i.); loggerhead sea turtle, *Caretta caretta*, (C.c.); leatherback sea turtle, *Dermochelys coriacea*, (D.c.); olive ridley sea turtle, *Lepidochelys olivacea*, (L.o.); flatback sea turtle, *Natator depressus*, (N.d.); and Kemp’s ridley sea turtle, *L. kempii*, (L.k.) with the global average across all turtles sampled in parentheses. The number of turtles surveyed (Nt) were obtained from the published literature and other sources as detailed in Appendix 1. In some instances a barnacle species was reported present (P) with a species of turtle but no host tally was recorded. Those barnacle taxa not reported occurring with a turtle species are indicated by the null set (ø). Additionally, the final column and row of the table presents, respectively, the index of increasing host selectivity for each barnacle (Sb) and the index of decreasing vulnerability to epibiosis for each species of sea turtle (Vt) (see text for derivation of indices).

Associated barnacle	Host turtle	*C.m.*	*E.i.*	*C.c.*	*D.c.*	*L.o.*	*N.d.*	*L.k.*	S_b_ (0–10)
Taxa	N_t_ (30,580)	20,911	5,288	2,679	691	569	328	114	
*Calyptolepas bjorndale*	(0.02)	0.02	ø	ø	ø	ø	ø	ø	8.6
*Chelonibia* sp.^a^	(4.29)	1.76	12.67	9.97	ø	1.23	ø	ø	
*C. caretta*	(6.86)	0.11	34.42	9.56	ø	ø	ø	ø	5.7
*C. testudinaria*	(16.97)	17.90	3.21	32.03	0.14	25.13	75.00	22.81	0.0
*C. ramosa* ^b^	(<0.01)	<0.01	ø	ø	ø	ø	ø	ø	8.6
*Chelolepas cheloniae* (syn. *Tubicinella cheloniae*)	(1.68)	0.17	9.00	0.07	ø	ø	0.30	ø	4.3
*Cylindrolepas darwiniana*	^f^(18.74)	27.41	ø	P	ø	0.18	ø	ø	5.7
*C. sinica*	(0.01)	0.01	P	P	ø	ø	ø	ø	5.7
*Platylepas* sp.^c^	(1.87)	0.51	1.46	2.24	ø	ø	100.00	ø	
*P. coriacea*	(1.72)	ø	ø	ø	76.12	ø	ø	ø	8.6
*P. decorata*	^f^(14.38)	20.58	1.68	ø	ø	1.05	ø	ø	5.7
*P. hexastylos*	(3.61)	3.41	0.32	9.48	ø	20.91	p	1.75	1.4
*Stephanolepas muricata*	(0.55)	0.09	P	5.41	ø	P	ø	ø	4.3
*Stomatolepas* sp.^d^	(0.03)	0.04	ø	ø	ø	ø	ø	ø	
*S. dermochelys*	(1.45)	ø	ø	ø	63.97	ø	ø	ø	8.6
*S. elegans* (syn. *S. praegustator*)	(1.58)	0.05	0.08	5.49	ø	55.36	1.52	0.88	1.4
*S. pilsbryi*	(0.01)	ø	ø	ø	0.29	ø	ø	ø	8.6
*S. pulchra* ^e^	(<0.01)	<0.01	ø	ø	ø	ø	ø	ø	8.6
*S. transversa*	(0.16)	0.24	ø	ø	ø	ø	ø	ø	8.6
	*V_t_* (10-0)		8.1	5.0	5.0	2.5	3.8	2.5	1.9

a Specific epithet not reported in sources but assumed herein not to be *C. ramosa* (see footnote 2).

b Validity uncertain, described from one specimen that was destroyed (see Korschelt 1933).

c Specific epithet not reported in sources but assumed herein not to be *P. coriacea* (see the text for justification).

d Probable unidentified species (see Pinou et al., 2013).

e Possible synonym of *S. transversa* (see Hayashi, 2013a).

f Value skewed upward by an indefinite amount (see the text for explanation).

### Barnacle perspective

**Fig. 3:**
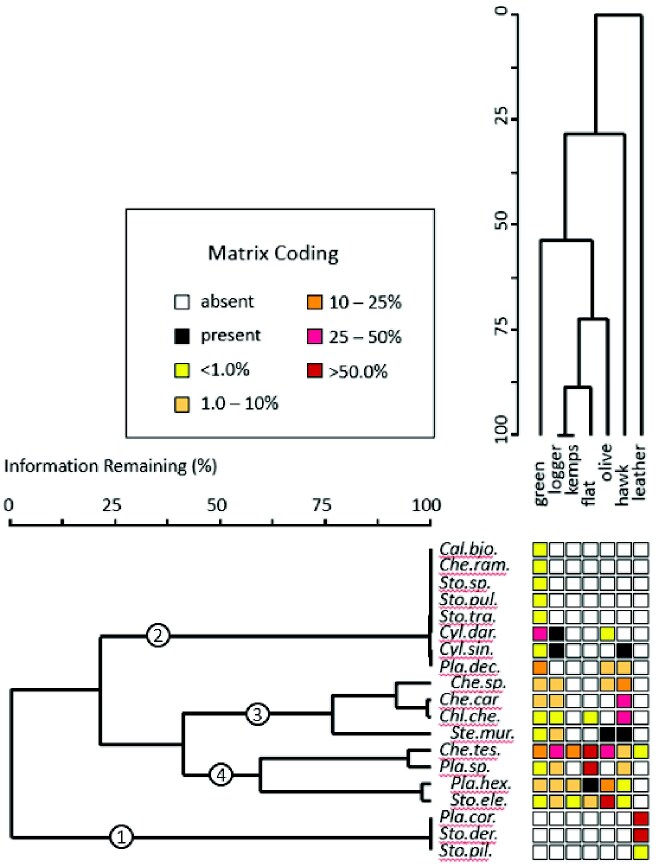
Hierarchical clustering of barnacle and sea turtle species by global percent occurrence for each barnacle/turtle pair using UPGMA Euclidean distances. Clustering of the seven sea turtle partners is displayed vertically and clustering of the 16 named barnacle partners is presented horizontally. Circled numbers identify four barnacle clusters: 1) barnacles specific to leatherbacks, 2) barnacles with few hosts, 3) barnacles with a medium number of hosts, and 4) barnacles with many hosts. The global percent occurrence for each pairwise association is color-coded according to the matrix binning scheme indicated.

The global percent occurrence of sea turtles hosting each barnacle taxon varied widely by species, revealing a continuum from highly selective to indiscriminate host preference by the barnacles. Hierarchical clustering by percent occurrence revealed differentiation of the barnacles into four broad groups based on the type and quantity of hosts they occupied (Fig. 3). The barnacles *C. testudinaria*, *P. hexastylos* (and perhaps other unidentified *Platylepas* species), and *S. elegans*, comprised a core set of barnacles common to all sea turtles except leatherbacks. Overall, most barnacles partnered with three turtle species each (avg. 2.9). The barnacle calculated to occur numerically more often than any other, was the geographically and host-restricted species *Cylindrolepas darwiniana*. This is a mathematical bias explained by two unusually large surveys of ≥ 12,000 green sea turtles in the Galapagos Islands (Green 1998; Beaumont et al. 2008) where this barnacle was present with 47% of individuals. Ignoring this result, the globally most common, widespread, and least selective barnacle was *C. testudinaria* (Table 1). The largest and most conspicuous barnacle of sea turtles, it was the only species found associated with all host turtles (albeit rarely with leatherbacks) and other non-turtle hosts. *Platylepas decorata* also occurred at a relatively high frequency but with only three host species. Results for this species were also similarly skewed by the large number of Galapagos samples mentioned above, along with the fact that it has probably been frequently confused with its congener *P. hexastylos*, the latter occurring more broadly across host species, appearing with all sea turtles except leatherbacks. Though not occurring as frequently, *S. elegans* was the only other barnacle to occur with six of the seven host species.

At the other extreme, seven barnacle species were recorded as highly selective, associating with only a single host species. Of these, four were reported from green and three from leatherback sea turtles. However, *Calyptolepas bjorndalae*, *Stomatolepas pulchra*, and *Chelonibia ramosa* are known only from single collection events (and only a single unverified specimen for the latter) and thus are of debatable taxonomic certainty. *Stomatolepas pilsbryi*, another solo-host affiliate, was described from only two leatherback sea turtles simultaneously, both from the same locality in the western Atlantic (Nova Scotia). *Stomatolepas transversa*, found in both the Atlantic and Pacific but in every case only with green sea turtles, most commonly occurred in the seams of the plastron and was the only indisputable single-host associate of a hard-shell turtle species. The two other single-host barnacles were *Platylepas coriacea* and *S. dermochelys*, both of which occurred only on the skin of leatherback turtles; existing at higher global frequencies with their hosts than any other barnacles. The barnacle *Chelonibia caretta* also deserves mention for its specificity; though found with hawksbill, green, and loggerhead sea turtles wherever these turtles occurred, it was by a large margin most often found with hawksbill sea turtles. Overall, *C. caretta* occurred more frequently with hawksbill sea turtles while its congener, *C. testudinaria*, was more common with loggerheads and greens. Occurrences of *C. caretta* on turtles other than hawksbills merits further scrutiny.

A few barnacles occurred at high rates only with certain turtle hosts at select locations. The barnacles *C. testudinaria* and *S. elegans* occurred with ≥50% individuals of particular host species in some locations. However, other than these two species, across a global scale, each barnacle species occurred with 35% or fewer individuals of any sea turtle species except in a large survey of the regionally restricted flatback sea turtle of which 100% of individuals hosted an unspecified platylepadid barnacle (Limpus et al. 1983). This was very likely *P. hexastylos* which has been reported occurring with flatback sea turtles previously (Monroe and Limpus 1979) and is the most common and abundant non-leatherback associate of the genus, though it could also have been *P. decorata*.

### Sea turtle perspective

Hierarchical clustering showed a stepwise differentiation of barnacle incidence among sea turtle species (Fig. 3). The leatherback sea turtle stood out as most distinctive in its barnacle associations due to being the sole host for *P. coriacea* and *S. dermochelys*. It was followed by the hawksbill sea turtle which was distinguished by a high incidence of intermediately-occurring barnacles, mostly *C. caretta* and *C. cheloniae*, two species that were infrequent or absent from other hosts. Green sea turtles, though hosting all common barnacles, differed from other hosts in their associations by the presence of several rare species not occurring with other turtles. Loggerhead, Kemp’s ridley, olive ridley, and flatback sea turtles clustered closest together, predominantly due to sharing barnacle species that are common across most sea turtles. Olive ridleys were separated somewhat from this group, possibly due to hosting the barnacles *C. darwiniana* and *P. decorata*, sharing the former only with greens and loggerheads and the latter only with greens and hawksbills.

Among all hosts, green sea turtles exhibited the highest diversity of epizoic barnacles, hosting 13 species, lacking only three nominal barnacles associating exclusively with leatherbacks. Thus, green sea turtles ranked highest on the epibiosis susceptibility index (Table 1). Most turtle species were found associated with 6–7 barnacle species (avg. 6.4). Hawksbill, loggerhead, and olive ridley sea turtles hosted an intermediate diversity of barnacles (eight, six, and five species, respectively), followed by leatherback and flatback turtles with four each. With only three associated barnacles, Kemp’s ridley turtles hosted the fewest barnacle species, registering the least susceptible to epibiosis. Leatherback sea turtles were noteworthy in being the only turtle species with nearly reciprocally exclusive barnacle associations. In other words, the barnacles found on leatherbacks were not present on other sea turtle species, except for the rare occurrence of *C. testudinaria*.

Green sea turtles occur in all tropical to subtropical regions of the world ocean and were sampled for barnacles more than any sea turtle species (68.4%), mainly in the Pacific (62.0%) and to a lesser extent in the Indo-Pacific (27.8%). Few were sampled in the Caribbean/Gulf of Mexico (6.8%) and very few from the Indian (2.1%) or Atlantic (1.0%) oceans and Mediterranean Sea (0.2%). *Chelonibia testudinaria* associated with green sea turtles wherever they occurred and overall was the most prevalent barnacle of greens. *Stomatolepas transversa* was the only barnacle specific to green sea turtles.

Hawksbill sea turtles, the second-most frequently sampled turtles worldwide (17.3%), were examined for barnacles in every ocean and sea except the Mediterranean where the species does not occur. Hawksbills in the Atlantic had the greatest diversity of barnacles where they associated with four species. Most hawksbills were assessed from the Caribbean/Gulf of Mexico (55.9%) and Indo-Pacific (33.5%) with smaller numbers from the Indian (5.5%) and Atlantic (4.8%) oceans and very few from the Pacific (0.4%). *Chelonibia* barnacles were by far the most common barnacle of hawksbills across all regions with *C. caretta* being the most prevalent species overall. In the Caribbean/Gulf of Mexico, *Chelonibia* barnacles were also most frequently encountered with hawksbill sea turtles, but most records did not distinguish between the barnacle species *C. caretta* and *C. testudinaria.* This obscures knowing whether *C. caretta* is the predominant barnacle of hawksbills in the Caribbean as it has been found to be in other regions. In surveys from the Persian Gulf of Iran, a remarkable contrasting distribution was recorded between two sites, both with robust samples sizes. At the island of Hormuz, *C. caretta* was the exclusive chelonibiid barnacle present with each of 41 hawksbill turtles surveyed (Devin and Sadeghi 2010); whereas, at Nakhiloo Island, ∼500 km away, *C. testudinaria* was the only barnacle encountered and present with all but one of 122 hawksbill turtles (Razaghian et al. 2019). Confirmation is needed whether one or the other sites represents a case of mistaken barnacle identity. *Platylepas hexastylos* is another barnacle that was widely reported epizoic in the skin of hawksbill sea turtles as well as the harmful boring barnacle *C. cheloniae* which was frequently found with hawksbills in the Indo-Pacific.

Loggerhead sea turtles were sampled relatively infrequently for barnacles (8.8%) among all sea turtles surveyed. They were assessed most often in the Mediterranean (61.7%) followed by the Atlantic (21.3%), Pacific (11%), and Indian (5.9%) oceans. Very few were sampled in the Caribbean/Gulf of Mexico (0.4%) and none in the Indo-Pacific. As with green sea turtles, *C. testudinaria* was the most common barnacle of loggerheads. It occurred in every region inhabited by the turtle and was the most prevalent species in most instances. The greatest diversity of barnacles on loggerheads was in the Atlantic and Mediterranean. It is notable that *S. elegans* was also found with loggerheads everywhere they were sampled.

Leatherbacks accounted for a small percentage of the sea turtles investigated for barnacles (2.3%). They were sampled for barnacles by an order of magnitude more often in the Pacific (87.0%) than in the Atlantic Ocean (7.4%). But, sampled, at least to a small degree, in every ocean region: Caribbean/Gulf of Mexico (3.6%), Mediterranean (1.3%), Indian Ocean (0.4%), and Indo-Pacific (0.3%). The barnacles *P. coriacea* and *S. dermochelys* were both exclusive to and common on leatherbacks worldwide. Though *S. dermochelys* was more common on leatherbacks overall, it was less common than *P. coriacea* in the Pacific. The reverse was true in the Atlantic. In the Atlantic, *C. testudinaria* was the only non-selective barnacle that was also recorded with leatherback sea turtles, albeit rarely.

Olive ridley sea turtles, which forage in the open sea, do not occur in the Caribbean/Gulf of Mexico nor in the Mediterranean Sea. However, from nesting data they are the most abundant sea turtle in the world’s oceans by an order of magnitude (SWOT online database). Relative to all sea turtles sampled for barnacles, few olive ridleys were examined (1.9%), probably due to their open ocean foraging habitus, and their diversity of barnacles was not high. They were assessed for barnacles almost exclusively in the Pacific Ocean (91.2%) where the barnacle *S. elegans* was its most common associate (60.7%) followed by *C. testudinaria* (27.6%) and *P. hexastylos* (22.9%). Olive ridley turtles were not examined for barnacles in the Atlantic Ocean nor in the Indo-Pacific but a few were surveyed in the Indian Ocean (8.8%) where they were reported lacking in barnacles altogether.

Flatback sea turtles are restricted to a single geographic region spanning the islands of the Torres Strait and northern Australia from the Indian to the Pacific Ocean. They were only examined for barnacles at a single location, Crab Island in the Arafura Sea off the York Peninsula, where they hosted four species (Limpus et al. 1983). The most common was an unspecified species of *Platylepas* followed closely by *C. testudinaria*. Uncommonly they hosted *S. elegans* and a single dead turtle was found with *C. cheloniae*.

Kemp’s ridley sea turtles, the least abundant sea turtle in the world and least inspected for barnacles, inhabit the most limited territory. Restricted to the Caribbean/Gulf of Mexico and northwestern Atlantic, they were mostly examined in the Gulf of Mexico (93.9%) where they nest and to a smaller degree in the western Atlantic (6.1%) where they occasionally roam. Kemp’s ridley turtles hosted three species of barnacles. *Chelonibia testudinaria*, their most frequent associate, but not in great numbers, was common in the Caribbean and Gulf of Mexico but mostly lacking from individuals in the Atlantic where it was replaced by *P. hexastylos* and *S. elegans*.

### Geographic perspective

In addition to host- and symbiont-associated patterns, there were macro-geographic components to barnacle occurrence as well, where some barnacles were found at different rates in different regions (Table 2). There was also at least one instance where a barnacle’s attachment mode differed between host species. Specifically, *S. elegans* was observed attached to external skin of the neck and body in olive ridleys from the Pacific but fastened to the tongue in loggerheads from the western Atlantic (Lazo-Wasem et al. 2011; Pinou et al. 2013).

**Table 2:** Correspondence of epibiont/basibont associations between the 7 sea-turtle species of the world and the 19 coronuloid barnacle taxa (16 named species) obligate with them, grouped by host turtle species (A-G) and apportioned among 6 ocean regions: Caribbean Sea and Gulf of Mexico (C-GoM), Atlantic Ocean (Atl), Mediterranean Sea (Med), Indian Ocean (Ind), Indo-Pacific (Indo-Pac), and Pacific Ocean (Pac). Values presented are percentages of turtles hosting each symbiont by region with the global average for that host species in parentheses. Numbers of turtles surveyed (NT) were obtained from the published literature and other sources as detailed in Appendix 1. In some instances a barnacle species is reported merely as present (P) because no host turtle tally was taken. Additionally, the last column and row of the table presents, respectively, the number of regions in which each species of barnacle occurs (NR) and the number of barnacle species occurring in each region (NB). Dashes (–) indicate regions in which a particular turtle species does not nest or regularly occur while null symbols (ø) designate regions where a turtle resides but for which no barnacle surveys have been conducted. A. Green sea turtle, *C. mydas*

Associated barnacle taxa	Ocean region	C-GoM	Atl	Med	Ind	Indo-Pac	Pac	N_R_
	N_T_ 20,911	1,432	213	35	448	5,811	12,972	
*Calyptolepas bjorndale*	(0.39)	0.00	2.35	0.00	0.00	0.00	0.00	1
*Chelonibia sp.*	(4.43)	23.81	0.00	0.00	2.68	0.02	0.10	
*C. caretta*	(5.35)	0.63	0.00	31.43	0.00	0.03	0.00	3
*C. testudinaria*	(25.93)	1.14	41.31	60.00	13.39	23.90	16.84	6
*C. ramosa*	(0.08)	0.00	0.47	0.00	0.00	0.00	0.00	1
*Chelolepas cheloniae* (syn. *Tubicinella cheloniae*)	(0.50)	0.00	0.00	0.00	2.68	0.22	0.09	3
*Cylindrolepas darwiniana*	(7.36)	0.00	0.00	0.00	0.00	0.00	44.18	1
*C. sinica*	(0.01)	0.00	0.00	0.00	0.00	0.03	0.00	1
*Platylepas sp.*	(4.99)	0.00	29.58	0.00	0.00	0.02	0.32	
*P. decorata*	(5.53)	0.00	0.00	0.00	0.00	0.00	33.17	1
*P. hexastylos*	(5.36)	0.14	14.09	0.00	13.17	0.00	4.79	4
*Stephanolepas muricata*	(0.02)	0.00	0.00	0.00	0.00	0.00	0.14	1
*Stomatolepas sp.*	(0.01)	0.00	0.00	0.00	0.00	0.00	0.07	
*S. elegans* (syn. *S. praegustator*)	(0.03)	0.07	0.00	0.00	0.00	P	0.08	3
*S. pulchra*	(<0.01)	0.00	0.00	0.00	0.00	0.02	0.00	1
*S. transversa*	(0.17)	0.00	P	0.00	0.45	0.02	0.36	4
	N_B_	4	6	2	4	7	8	

**Table:** B. Hawksbill sea turtle, *E. imbricata*

Associated barnacle taxa	Ocean region	C-GoM	Atl	Med	Ind	Indo-Pac	Pac	*N_R_*
	N_T_ 5,288	2,954	253	—	293	1,769	19	
*Chelonibia* sp.	(4.60)	22.65	0.00	—	0.34	0.00	0.00	
*C. caretta*	(43.40)	2.37	79.05	—	26.28	82.98	26.32	5
*C. testudinaria*	(12.07)	1.29	0.79	—	42.32	0.17	15.79	5
*Chelolepas cheloniae* (syn. *Tubicinella cheloniae*)	(5.44)	0.00	0.00	—	0.34	26.85	0.00	2
*Cylindrolepas sinica*		0.00	0.00	—	0.00	0.00	P	1
*Platylepas* sp.	(3.04)	1.22	0.00	—	13.99	0.00	0.00	
*P. decorata*	(2.69)	2.95	0.00	—	0.00	0.00	10.53	2
*P. hexastylos*	(7.04)	0.27	1.58	—	0.00	P	26.32	4
*Stephanolepas muricata*		0.00	0.00	—	0.00	P	P	2
*Stomatolepas elegans* (syn. *S. praegustator*)	(0.10)	0.10	0.40	—	0.00	0.00	0.00	2
	N_B_	5	4		3	5	6	

**Table:** C. Loggerhead sea turtle, *C. caretta*

Associated barnacle taxa	Ocean region	C-GoM	Atl	Med	Ind	Indo-Pac	Pac	N_R_
	N_T_ 2,679	10	571	1,654	158	0	286	
*Chelonibia* sp.	(7.74)	0.00	2.98	12.94	22.78	ø	0.00	
*C. caretta*	(6.10)	0.00	21.19	7.92	0.00	ø	1.40	3
*C. testudinaria*	(26.51)	10.00	68.13	24.49	17.72	P	12.24	6
*Cylindrolepas darwiniana*		0.00	P	0.00	0.00	ø	P	2
*Cylindrolepas sinica*		0.00	0.00	0.00	0.00	ø	P	1
*Platylepas* sp.	(0.75)	0.00	0.18	3.57	0.00	ø	0.00	
*P. hexastylos*	(7.53)	P	13.31	9.61	1.27	ø	5.94	5
*Stephanolepas muricata*	(2.53)	0.00	0.18	7.92	0.00	ø	4.55	3
*Stomatolepas elegans* (syn. *S. praegustator*)	(3.46)	P	4.90	6.83	0.00	ø	2.10	4
	N_B_	3	6	5	2	1	7	

**Table:** D. Leatherback sea turtle, *D. coriacea*

Associated barnacle taxa	Ocean region	C-GoM	Atl	Med	Ind	Indo-Pac	Pac	N_R_
	N_T_ 691	25	51	9	3	2	601	
*Chelonibia testudinaria*	(0.33)	0.00	1.96	0.00	0.00	0.00	0.00	1
*Platylepas coriacea*	(50.01)	36.00	31.37	0.00	100.00	50.00	82.70	5
*Stomatolepas dermochelys*	(45.18)	4.00	60.78	22.22	66.67	50.00	67.39	6
*S. pilsbryi*	(1.31)	ø	3.92	0.000	ø	ø	0.00	1
	N_B_	2	4	1	2	2	2	

**Table:** E. Olive ridley sea turtle, *L. olivacea*

Associated barnacle taxa	Ocean region	C-GoM	Atl	Med	Ind	Indo-Pac	Pac	N_R_
	N_T_ 569	―	ø	―	50	ø	519	
*Chelonibia* sp.	(0.67)	―	ø	―	0.00	ø	1.35	
*C. testudinaria*	(13.78)	―	ø	―	0.00	ø	27.55	1
*Cylindrolepas darwiniana*	(0.06)	―	ø	―	0.00	ø	0.19	1
*Platylepas decorata*	(0.58)	―	ø	―	0.00	ø	1.16	1
*P. hexastylos*	(11.46)	―	ø	―	0.00	ø	22.93	1
*Stephanolepas muricata*		―	ø	―	0.00	ø	P	1
*Stomatolepas elegans* (syn. *S. praegustator*)	(60.69)	―	ø	―	P	ø	60.69	2
	N_B_	―	ø	―	1	ø	6	

**Table:** F. Flatback sea turtle, *N. depressus*

								
Associated barnacle taxa	Ocean region	C-GoM	Atl	Med	Ind	Indo-Pac	Pac	N_R_
	N_T_ 328	―	―	―	ø	328	ø	
*Chelonibia testudinaria*	(75.00)	―	―	―	ø	75.00	ø	1
*Chelolepas cheloniae* (syn. *Tubicinella cheloniae*)	(0.30)	―	―	―	ø	0.30	ø	1
*Platylepas* sp.^a^	(100.0)	―	―	―	ø	100.00	ø	
*P. hexastylos*		―	―	―	ø	P	ø	1
*Stomatolepas elegans* (syn. *S. praegustator*)	(1.52)	―	―	―	ø	1.524	ø	1
	N_B_	―	―	―	ø	4	ø	

a Possibly *P. hexastylos* or *P. decorata*.

**Table:** G. Kemp’s ridley sea turtle, *L. kempii*

								
Associated barnacle taxa	Ocean region	C-GoM	Atl	Med	Ind	Indo-Pac	Pac	N_R_
	N_T_ 114	107	7	―	―	―	―	
*Chelonibia testudinaria*	(18.83)	23.36	14.29	―	―	―	―	2
*Platylepas hexastylos*	(7.61)	0.93	14.29	―	―	―	―	2
*Stomatolepas elegans* (syn. *S. praegustator*)	(7.14)	0.00	14.29	―	―	―	―	1
	N_B_	2	3	―	―	―	―	—

Of barnacles associated with sea turtles, there were on average 9.2 species per geographic region, with diversity highest in the Pacific Ocean (12 species) and lowest in the Mediterranean Sea (6 species). The rate of incidence of turtles with barnacles, averaged across all turtle and barnacle species, was highest in the Mediterranean and the Atlantic (9.2% and 9.1%, respectively). It was lowest for the Caribbean/Gulf of Mexico (2.9%) and intermediate for the Pacific, Indian, and Indo-Pacific (7.2%, 5.4%, and 4.2%, respectively). However, the accuracy of this measure is questionable and must be interpreted cautiously since individual turtles hosting multiple barnacle species were tabulated as separate host occurrences for each barnacle species. Only four barnacle species were found in all geographic regions (*C. caretta*, *C. testudinaria*, *P. hexastylos*, and *S. dermochelys*) but, except for the last, each was not always primarily associated with the same hosts in each region. For instance, the barnacle *P. hexastylos* in the Pacific was hosted more frequently by hawksbill (26.3%) and olive ridley (22.9%) sea turtles but in the Atlantic more often by green, loggerhead, and Kemp’s ridley sea turtles in proportions nearly equal to each other (14.1%, 13.3%, and 14.3%, respectively). And *S. muricata* predominantly occurred with green turtles in the major oceans of the world and Caribbean Sea whereas in the Mediterranean it was reported only from loggerheads and in the Indo-Pacific from hawksbill and flatback sea turtles.

*Cylindrolepas darwiniana* and *P. decorata* were the most commonly reported barnacles of sea turtles in the Pacific (39.8% and 29.9%, respectively), biased by their high occurrence in the unusually large samples from the Galapagos Islands mentioned above. Otherwise, *C. testudinaria* was the barnacle most commonly associated with sea turtles in the Pacific (16.4%).

Within the Atlantic Ocean were found 11 barnacle species associated with sea turtles, of which *C. testudinaria* was the most prevalent by a wide margin (43.9%), followed by *C. caretta* (29.3%), then *P. hexastylos* (10.1%). *Chelonibia caretta* was strongly associated with hawksbill (79.1%) and to a lesser degree loggerhead sea turtles (21.2%) while *C. testudinaria* was most often found with loggerhead (68.1%), followed by green (41.3%), followed by Kemp’s ridley (14.3%), sea turtles.

Of eight barnacle species recorded from the Caribbean/Gulf of Mexico, *Chelonibia* barnacles occurred most commonly overall (22.3%) but whether *C. caretta* or *C. testudinaria* predominated was not detectable due to a lack of specificity in identifications. All other barnacle species found in the region occurred with ≤2.0% of sampled sea turtles.

More sea turtles of the Mediterranean Sea hosted *C. testudinaria* (25.1%) than any other barnacle species. *Platylepas hexastylos*, *C. caretta*, and *S. muricata* were next most prevalent (9.4%, 8.4%, and 7.7%, respectively). Interestingly, the barnacle *P. coriacea*, commonly associated with leatherbacks throughout the world, was not reported from the Mediterranean. This may be due to limited sampling since the species has an otherwise robust global presence.

Of the seven barnacles most commonly occurring on sea turtles in the Indian Ocean, *C. testudinaria* (22.3%) was most common followed by *P. hexastylos* (6.4%). Surprisingly, *S. elegans*, though never highly prevalent but nevertheless widespread on multiple turtle species, was only reported from olive ridleys in the Indian Ocean. This too may be an artifact of under sampling.

The Central Indo-Pacific was another area of relatively high diversity, with 11obligate sea-turtle barnacles. *Chelonibia testudinaria* was most prevalent (20.7%), followed closely by *C. caretta* (18.6%). The boring barnacle *C. cheloniae*, only known to range from the Indian to western Pacific Oceans, was modestly present in the center of its range (6.2%). *Stomatolepas pulchra* recorded only once from a single site in the South China Sea (Ren 1980) may be an endemic species though it is likely a synonym of *S. transversa* (Hayashi 2013a). If endemic, it would have a distribution similar to *Cylindrolepas sinica*, known only from the South China Sea to the western and perhaps central-Pacific (Zardus and Balazs 2007; Hayashi 2009, 2013b).

## Discussion

### Barnacle substratum selectivity

The present analysis, confirms that a number of coronuloid barnacles are obligate epizoites of sea turtles, but illuminates somewhat of a paradox. Despite affiliating exclusively with particular sea turtles, many barnacle species exhibit relaxed fidelity for several turtle species, associating with overlapping suites of hosts, but with each symbiont occupying a virtually unique host subset. This elasticity might not be surprising were the barnacles all inhabiting turtle hosts in a similar way. But the various barnacle species exploit different microhabitats on turtles, glueing to shell, clamping to skin, or penetrating one or the other of these parts, raising the question *why have more of them not become specific to single hosts?* Host substratum may or may not vary substantially among turtle species. Both the yielding substratum of skin and the firm surface of shell are composed of keratin (Block and Bolling 1939; Solomon et al. 1986; Alibardi 2005; Kardong 2019), yet these two portions of the turtle body certainly differ physically and perhaps chemically as well. On observation, the surface micro-structure of turtle shell also appears different between species but how biomechanical or biochemical properties vary among hosts and between their parts have not been characterized. Regardless, the key to barnacle flexibility may lie not so much in their ability to detect properties of the substratum but in the versatility of the larval attachment organs, the antennules of the cyprid stage, which across disparate taxa are highly similar in form and structure, targeting substrata that is animate or inanimate, yielding or firm (Al-Yahya et al. 2016; Dreyer et al. 2020). Mechanically assessing the substratum, these larval organs also chemically sense and respond in complex ways to the presence of compounds from conspecific individuals and microbial biofilms (Hadfield 2011), in some cases strengthening adhesion as a response (Zardus et al. 2008). This may in part explain, for instance, how *C. testudinaria* attaches to keratinous, chitinous, and artificial substrates alike, allowing the maintenance of high host plasticity for millions of generations without specializing (Ewers-Saucedo et al. 2017).

This high versatility in attachment begs the question *why are turtle barnacles not found more often on other kinds of hosts?* The most common turtle barnacle, *C. testudinaria*, is the least selective, being the only species reported from all sea turtles. It is also the only “turtle” barnacle occurring on non-turtle hosts excepting the single report of *P. hexastylos* on a fish (Ryder 1879). *Chelonibia testudinaria* routinely occurs on various crabs and occasionally on sirenians, crocodilians, and artificial substrata (Zardus et al. 2014). Evolutionary radiation within the Coronuloidea has generated several species of “whale barnacles” that specialize on cetaceans (Scarff 1986; Collareta et al. 2016) and a pair of “sea snake” barnacles (Lanchester 1902; Daniel 1958). But there are no records of Coronuloid barnacles from other prospective living hard substrates such as mollusks or echinoids. Stalked barnacles by comparison are epizoic with a much wider variety of animals (e.g., jellyfish [Pagès 2000], sea urchins [Grygier and Newman 1991], mollusks [Landman et al.1987; Kolbasov and Zevina 1999], sharks [Rees et al. 2019], and opportunistically, pinnipeds, penguins [Reisinger and Bester 2010], and fish [Pilsbry 1907; Sumner et al. 1913; Crozier 1916]). The contrasting question also needs asking: *Why are non-obligate, hard-substratum barnacle species not found more frequently on sea turtles?* They are occasionally reported, mostly other balanomorph species in the Balanidae (Bugoni et al. 2001; Frick et al. 2004; Hayashi 2017) but also lepadomorph species (Eckert and Eckert 1987; Tachikawa 1995; Frick et al. 1998; Kitsos et al. 2005) and rarely verrucomorphs (Miranda and Moreno 2002). But more often than not, when present, these opportunists are found attached to underlying coronuloid barnacles, not to turtle shell directly.

### Single-host barnacles

A few turtle barnacles are very host-selective, associating with only a single species of turtle. For instance, all barnacles occurring with leatherback sea turtles, except for *C. testudinaria*, are found with no other turtles. This degree of selectivity is perhaps not surprising since leatherbacks differ greatly from all other turtles, both phylogenetically and morphologically. The evolutionarily earliest extant sea turtle, the leatherback, pre-dates all others by ∼45 mya or more (Cadena and Parham 2015) and instead of being shielded by a shell of keratinous scutes, it is covered by dermis. Somewhat surprising is that among barnacles of hard-shelled sea turtles, excluding single and unverified reports, the barnacle *S. transversa* is the only single-host species, associating exclusively with the green sea turtle, occupying the very particular niche of the seams of the plastron. This species of turtle is noteworthy in being the most susceptible to barnacle epibiosis, hosting the widest variety of coronuloid cirripeds. At the other extreme, leatherback and olive ridley sea turtles host a low diversity of barnacles, possibly due to their lifestyle of spending most of their time in the open ocean where acorn barnacle larvae are likely less abundant. Stalked barnacles particularly, a component of the open ocean rafting community, are an indication of pelagic existence and are common on leatherbacks (Hughes 1970; Eckert and Eckert 1988; Robinson et al. 2017b), olive ridleys (Robinson et al. 2017a, 2017b), and distantly-straying Kemp’s ridley sea turtles (Covelo et al. 2016). Ridley sea turtles are the most recently evolved sea turtles (Dutton et al. 1996; Naro-Maciel et al. 2008) and are known for their waxy surfaces (Weldon et al. 1990), which may be a deterrent to epibiosis, especially Kemp’s ridleys which spend much of their lives near coastlines but host the lowest diversity of barnacles of any sea turtle. The barnacle *S. muricata* has been found associated in the South Pacific with open-ocean loggerhead sea turtles but not with coastal residents (Limpus and Limpus 2003). Sea turtle natural history may also influence rates of epibiosis. Both ridley species nest in *arribadas* or synchronized, mass assemblages, though olive ridleys can also be solitary nesters (Dornfeld et al. 2015). Synchronized nesting may reduce chances for barnacle attachment by narrowing the window of recruitment opportunity in a region. Activities of mass nesting may also increase barnacle removal by dislodgement (Robinson et al. 2019). But, whether olive ridley turtles carry more barnacles in solitary versus mass nesting populations has not been investigated.

### Geographic overlap

The geographic distributions of sea turtles and barnacles overlap but not exactly. Most sea turtles and many of the barnacles occur worldwide but with gaps for various members of each in some regions. In general, barnacles exhibit a greater degree of localization than sea turtles, there being only two species of highly regional turtles (Kemp’s ridley and flatback sea turtles) and perhaps six species of regional barnacles. Thus, presenting barnacle incidence rates at large scales for some species is not meaningful. For instance, *S. transversa*, exclusive to green sea turtles, is reported both from the Atlantic and Pacific; but, in the Pacific it has only been reported from localities in the western Pacific and Indo-Pacific (Nilsson-Cantell 1930; Monroe and Limpus 1979; Hayashi 2012) and in the Atlantic only from Paraíba State in Brazil (Young 1991). A similarly disjunct and enigmatic Atlantic-Pacific distribution has been found among genotypes of *S. elegans* (Pinou et al. 2013) that cannot be simply explained by linkage through the Panama canal which lies 26 m above sea level and extends 82 km through freshwater Gatun Lake. Barnacle distributions, in addition to being patchy in space, can also be uneven in density, spread perhaps over large areas in generally low abundance but present in high levels at certain locations. For example, *C. darwiniana* is present in the western Atlantic in low numbers and from both sides of the Pacific associated with several hosts but present on virtually 100% of nesting green sea turtles in the Galapagos Islands (Zullo 1991). *Cylindrolepas sinica* on the other hand appears localized to a single area, perhaps just a few islands, of the South China Sea (Ren 1980). Yet others, while not abundant, are widely but discontinuously distributed, in particular *S. muricata* globally (Frick et al. 2011) and *C. cheloniae* in the Indo-Pacific (Monroe and Limpus 1979 [as *T. cheloniae*]; Nolte et al. 2020). The dual dispersion experienced by epizoic barnacles, distributed first as larvae in the plankton for several weeks then as adults transported by their hosts, may account for some of these discontinuities.

### Evolutionary patterns

The evolutionary route taken by barnacles in specializing on sea turtles is not entirely clear. Pairwise associations between host and symbiont do not conform to phylogenetic affiliations among the turtles and especially not among the barnacles, as evidenced by scattered patterns in hierarchical clustering of occurrence data (Fig. 3). The leatherback sea turtle hosts the majority of highly selective barnacles which, however, belong to two platylepadid genera that are widely spread across all sea turtles. Though fossil remains suggest chelonibiid barnacles are basal, there is evidence that platylepadids preceded them (Hayashi et al. 2013) and radiated to all turtle hosts. In spite of historical legacies, the underlying template for barnacle/sea turtle associations may be driven by ecology more than phylogeny. Differences in diet and habitat of the hosts may affect barnacle recruitment and survival. Life on a benthic-feeding omnivore that disrupts the benthos while foraging may amplify food quantity and quality for barnacles compared to an herbivorous host foraging in sea grass meadows or a pelagic feeder in the open sea. But which host feeding-mode is most optimal for barnacles is debatable. Herbivorous green sea turtles host the greatest diversity of barnacles whereas omnivorous loggerheads may host the highest barnacle loads and leatherbacks the most widespread species. If narrow host use is a clue, investigating the biology of barnacles tending toward specialization might provide further insight.

### Knowledge gaps

Many unknowns remain concerning the association of barnacles with sea turtles. A more complete picture requires further study of both symbionts and hosts, but our knowledge gap of the former is probably larger. To advance understanding, several areas deserve greater attention and exploration. Barnacle shell growth rates, to-date studied only for *C. testudinaria* (Sloan et al. 2014; Doell et al. 2017), could provide insight on short-term or seasonal movements of sea turtles. The idea of harnessing barnacles to track host movements and trace population boundaries has been explored in California gray whales (Killingley 1980) and sea turtles (Killingley and Lutcavage 1983; Detjen et al. 2015; Pearson et al. 2019, 2020), using isotopic analysis of barnacle shells to infer salinity and temperature signatures of the water inhabited by their hosts over time. Barnacle age structure and infestation rates have also been tested as geospatial indicators for dolphins (Di Beneditto and Ramos 2000). A deeper knowledge of shell growth for other barnacles and the natural history of larval dispersal for any species could further progress. It would be highly valuable to know how the supply of barnacle larvae varies in space a time and could, for instance, help explain the anomaly of different species of *Chelonibia* on hawksbill turtles found across a relatively short distance in the Persian Gulf of Iran (compare Devin and Sadeghi 2010 to Razaghian et al. 2019). Indeed, where barnacle larvae are distributed and make their rendezvous with sea turtles is perhaps the most crucial and unresolved aspect of the association. Assessing suites of barnacles present on sea turtles might also offer a community level “barnacle fingerprint” mechanism for matching snapshots of epibiont diversity to regional patterns. So might also assaying the distribution of barnacle population genotypes among sea turtles. How barnacle presence varies seasonally or with ontogenetic stages or between genders has also not been thoroughly explored. Potential ecological succession of barnacles has been suggested between home-range and migrating Kemp’s ridleys (Lutcavage and Musick 1985), newly recruiting loggerheads (Limpus and Limpus 2003), and across a nesting interval for female loggerhead sea turtles (Frick et al. 2002). It would also be valuable to examine attachment mode among barnacles and ascertain, as with *S. elegans* (compare Lazo-Wasem et al. 2011 with Pinou et al. 2013), how it may vary with host species.

On the sea turtle side of the association, a lack of comparative information on scute growth, scute shedding, and epidermal turnover, and the impact of these on barnacle attachment, presents a major gap in understanding. As a general model, scutes of turtles grow along their margins and from beneath while shedding layers superficially (Zangerl 1969). But differences in details among host species may influence adaptations in barnacles. Hawksbill sea turtles for instance add new material along the anterior margin of the scutes while material at the posterior edge is eroded but not sloughed and the scutes grow continuously thicker from beneath with age (Tucker et al. 2001). In contrast, loggerhead and green turtles maintain relatively thin scutes by sloughing outer layers at indeterminate rates (López-Castro et al. 2014). As I have observed, the former sheds continuously in flakes or pieces and the latter periodically in large thin sheets. But lacking more detailed characterization of scute growth and shedding, how barnacles respond to different circumstances cannot at present be elucidated.

Results of this study are inevitably constrained by certain limitations and caveats of the data. Information on the occurrence of barnacles on sea turtles comes primarily from nesting females, thus patterns may vary for pelagic individuals, including males and juveniles, which are less easily sampled. Records are also not distributed equally across host species, thus uneven effort may have generated artificial differences between species. That barnacles are not mentioned in many reports of sea turtles does not necessarily indicate their absence, while their presence on the other hand, when recorded, cannot be understood as a complete inventory of the barnacle diversity present, unless stated otherwise in either case. It is likely that greater attention has been paid to the obvious, shell-cementing *Chelonibia* barnacles. Easy-to-miss species of the skin and those in hard-to-reach places such as the plastron, base of limbs, or mouth, are probably under-represented in many records. A large-scale and consistent lack of reports of some barnacles in some locations of the world or with some turtle hosts likely reflects actual distributions but artifacts of underreporting always remain a possible concern, especially for uncommon species. Another significant assumption is that the turtles, and more especially the barnacles, are correctly reported. In this study, corrections were applied for the analysis where apparent. In particular, one common assumption was that *Platylepas* barnacles on leatherbacks, often reported as *P. hexastylos*, were scored as *P. coriacea*. If incorrect, this assumption overinflates the specificity of these species of barnacles.

In summary, the association of epizoic coronuloid barnacles with sea turtles is both stringent and flexible; narrowly obligate to sea turtles but not tightly linked with single species of hosts except for a few cases. The barnacles are also more taxonomically diverse and geographically subdivided than their chelonian partners, a fact which could be utilized to give insight on where sea turtles travel and how they operate in the environment by tracing various distributions of their barnacle epifauna. The sea turtle/epibiont relationships described herein also offer a valuable baseline for monitoring redistributions of hosts and symbionts in a time of changing climate.

## Supplementary Material

obab002_Supplementary_Data
