# Corrigendum to: A Global Synthesis of the Correspondence Between Epizoic Barnacles and Their Sea Turtle Hosts

**DOI:** 10.1093/iob/obab018

**Published:** 2021-07-16

Corrigendum to: “A Global Synthesis of the Correspondence Between Epizoic Barnacles and Their Sea Turtle Hosts” by John D Zardus.

*Integrative Organismal Biology*, Volume 3, Issue 1, 2021, obab002, https://doi.org/10.1093/iob/obab002

In the originally published version of this manuscript, the truncation of some generic names in Tables 1 and 2 had introduced errors due to several genera starting with the same letter.

This has now been corrected, and each genus in the two Tables has been spelled out in full.

