# Supplementary material for: A Global Synthesis of the Correspondence Between Epizoic Barnacles and Their Sea Turtle Hosts
Source: Integr Org Biol. 2021 Feb 5;3(1):obab002. doi: 10.1093/iob/obab002 (PMC8077887; doi:10.1093/iob/obab002)
Supplement: obab002_Supplementary_Data [file obab002_supplementary_data.pdf]

Appendix I

Catalog of Source Material used in Enumerating Barnacle Occurrence from Sea Turtles

References and sources are numbered and coded by record and data type. Annotations are provided that summarize the quantitative or qualitative information associated with the source along with ocean region and locality where given. Corresponding turtle counts are arranged in columns coded by turtle species (**CC,** *Caretta caretta* ; **CM**, *Chelonia mydas* ; **DC,** *Dermochelys coriacea* ; **EI**, *Eretmochelys imbricata* ; **LK**, *Lepidochelys kempii* ; **LO**, *L. olivacea* ; and **ND**, *Natator depressus*) and by barnacle species (***Che.car.**, Chelonibia caretta;* ***Che.tes.**, C. testudinaria;* ***Che.ram.**, C. ramosa;* ***Che.sp.**, unidentified Chelonibia;* ***Clp.che.**, Chelolepas cheloniae;* ***Cal.bjr.**, Calyptolepas bjorndalae;* ***Cyl.dar.**, Cylindrolepas darwiniana;* ***Cyl.sin.**, C. sinica;* ***Pla.cor.**, Platylepas corieacea;* ***Pla.dec.**, P. decorata;* ***Pla.hex.**, P. hexastylos;* ***Pla.sp.***, unidentifed *Platylepas;* ***Ste.mur.**, Stephanolepas muricata;* ***Sto.drm.**, Stomatolepas dermochelys;* ***Sto.ele.**, S. elegans;* ***Sto.pil.**, S. pilsbryi;* ***Sto.pul.**, S. pulchra;* ***Sto.tra.**, S. transversa* , and ***Sto.sp.***, unidentified *Stomatolepas).* Turtles with barnacle species present but not quantified are indicated with a plus sign (**+**) while barnacles that could have been present but were not found or recorded are designated by a zero (**0**). Shaded cells denote entries that are corrected or altered from the original source as explained in the annotation: either an estimated value, inferred species, synonymized name, misapplied name, or miscalculated count.

Ref. No.	Reference/Source	Record Type	Data Type	Annotation	Ocean Region	Locale
1.	Affronte, M. & Scaravelli, D. (2001). Analysis of stranded sea turtles in the north-western Adriatic sea. *Zoology of Middle East* **24,** 101-108.	peer	survey	95 small CC turtles, all with heavy cover of *Che.tes.* (*Pla.hex.* not listed but visible in Fig. 6).	Mediter.	Adriatic
2.	Aguirre, A.A., Balazs, Zimmerman, B., & Spraker, T.R. (1994). Evaluation of Hawaiian green turtles (*Chelonia mydas*) for potential pathogens associated with fibropapillomas. *Journal of Wildlife Diseases* **30**, 8-15.	peer	record	CM turtles with *Che.tes., Pla.hex.* and *Ste.mur.*	Pacific	Hawaii
3.	Alexander, J., Garrett, K., Garner, S.A., Conrad, J., & Coles, W. (2004). Tagging and nesting research on leatherback sea turtle (*Dermochelys coriacea*) on Sandy Point, St. Croix, U.S. Virgin Islands, 2004, Annual Report to Fish and Wildlife Service, **40.**	tech report	record	100 DC turtles, some with *Pla.hex.* (scored herein as *Pla.cor.*) and *Sto.drm.*	Carib.	St. Croix
4.	Alonso, L., Estrades, A., Scarabino, F., & Calcagno, J.A. (2010). *Conchoderma virgatum* (Spengler, 1790) (Cirripedia: Pedunculata) associated with sea turtles in Uruguayan shallow coastal waters. *Pan-American Journal of Aquatic Sciences* **5**, 166-168.	peer	record	185 CM turtles, some with *Pla.hex.*	Atlantic	Uruguay, 3 islands of
5.	Alonso, L., Calcagno, J.A., & Scarabino, F. (2010). Epibionts associated with juvenile green turtle (*Chelonia mydas*) from the foraging and developmental area of Cerro Verde, Uurguay. Twenty-Eighth Annual Symposium on Sea Turtle Biology and Conservation. Loreto, Baja California Sur, México, NOAA Technical Memorandum NMFS-SEFSC-602, 2-3.	tech report	survey	87 CM turtles, 13 with *Che.tes.* , 63 with *Pla.sp.*	Atlantic	Uruguay
6.	Angulo-Lozano, L., Nava-Duran, P.E., & Frick, M.G. (2007). Epibionts of olive ridley turtles nesting at Playa Ceuta, Sinaloa, México. *Marine Turtle Newsletter* **18,** 13-14.	peer	survey	12 LO turtles, some with *Che.tes.*	Pacific	Mexico, Sinaloa, Playa Ceuta

Ref. No.	total turtles analyzed by species							counts of turtles hosting corresponding species of barnacles																		
	CC	CM	DC	EI	LK	LO	ND	*Che.car.*	*Che.tes.*	*Che.ram.*	*Che.sp.*	*Chl.che.*	*Cal.bjr.*	*Cyl.dar.*	*Cyl.sin.*	*Pla.cor.*	*Pla.dec.*	*Pla.hex.*	*Pla.sp.*	*Ste.mur.*	*Sto.drm.*	*Sto.ele.*	*Sto.pil.*	*Sto.pul.*	*Sto.tra.*	*Sto.sp.*
1.	95							0	95			0		0	0		0	+		0						
2.		+						0	+	0		0	0	0	0		0	+		+				0	0	
3.			+						0							+					+		0			
4.		+						0	0	0		0	0	0	0		0	+		0		0		0	0	
5.		87						0	13	0		0	0	0	0				63	0		0		0	0	
6.						+			+					0			0	0		0		0				

Ref. No.	Reference/Source	Record Type	Data Type	Annotation	Ocean Region	Locale
7.	Annandale, N. (1905). Report on the Cirripedia collected by Professor Herdman at Ceylon in 1902, in Re*port of the Government of Ceylon on Pearl Oyster Fisheries of Gulf of Manaar Suppl.* **5**, 137-150 + 9 figures.	tech report	list	EI turtles, some with *Che.tes.*	Indian	Laccadive Sea, Sri Lanka
8.	Bacon, P.R. (1970). Studies on the leatherback turtle, *Dermochelys coriacea* (L.), in Trinidad, West Indies. *Biological Conservation* 2, 213-217.	peer	record	DC turtles, some with *Pla.sp.* (scored herein as *Pla.cor.)*	Atlantic	Trinidad
9.	Bacon, P. R. (1976). The Cirripedia of Trinidad. St*udies on the Fauna of Curaçao and other Caribbean Islands* , No. 163 **50,** 3-55.	peer	list	CC turtles, some with *Ch.car, Che.tes.* ; DC turtles, some with *Pla.hex.* (scored herein as *Pla.cor.*)	Atlantic	Trinidad
10.	Badillo, F.J., Aznar, F.J., Tomás, J., & Raga, J.A. (2003). Epibiont fauna of Caretta caretta in the Spanish Mediterranean. Proceedings of the First Mediterranean Conference on Marine Turtles. D. Margaritoulis and A. Demetropoulos. Rome, Italy, Instituto Centrale per la Ricerca scientifica e tecnologica Applicata al Mare/International Union for Conservation of Nature, Cyprus, 62-66.	tech report	survey	13 CC turtles, 11 with *Pla.hex.*	Mediter.	Valencia, Spain
-	Badillo Amador, F. J. (2007). Epizoítos y parásitos de la tortuga boba (*Caretta caretta*) en el Mediterráneo Occidental. Facultat de Ciencies Biologiques. Valencia, Spain, Univesitat de Valencia. Ph.D., 262 pp.			duplicate data (see Domenech et al. 2015)		
11.	Balazs, G.H. (1978). A hawksbill turtle in Kaneohe Bay, Oahu. *Elepaio* **38,** 128-129.	peer	record	1 EI turtle with *Che.tes.* and *Ste.mur.*	Pacific	Hawaii, Oahu, Kaneohe Bay
12.	Balazs, G.H. (1980). Synopsis of the biological data on the green turtle in the Hawaiian Islands. N*OAA Technical Memorandum NMFS, National Marine Fisheries Service* **7**, 1-141.	tech report	record	CM turtles, some with *Che.tes., Pla.hex.* and *Ste.mur.*	Pacific	Hawaii

Ref. No.	total turtles analyzed by species							counts of turtles hosting corresponding species of barnacles																		
	CC	CM	DC	EI	LK	LO	ND	*Che.car.*	*Che.tes.*	*Che.ram.*	*Che.sp.*	*Chl.che.*	*Cal.bjr.*	*Cyl.dar.*	*Cyl.sin.*	*Pla.cor.*	*Pla.dec.*	*Pla.hex.*	*Pla.sp.*	*Ste.mur.*	*Sto.drm.*	*Sto.ele.*	*Sto.pil.*	*Sto.pul.*	*Sto.tra.*	*Sto.sp.*
7.				+				0	+			0			0		0	0		0		0				
8.			+						0							+					0		0			
9.	+							+	+			0		0	0		0	0		0		0				
			+						0							+					0		0			
10.	13							0	0			0		0	0		0	11		0		0				
-																										
11.				1					1											1						
12.		+						0	+	0		0	0	0	0		0	+		+		0		0	0	

Ref. No.	Reference/Source	Record Type	Data Type	Annotation	Ocean Region	Locale
13.	Balazs, G.H. (1985). Status and ecology of marine turtles at Johnston Atoll. *Atoll Research Bulletin* **285,** 1-46.	peer	record	21 CM turtles, 2 with *Pla.hex.*	Pacific	Johnston Atoll
14.	Balazs, G.H., Forsyth, R.G., & Kam, A.K.H. (1987). Preliminary assessment of habitat utilization by Hawaiian green turtles in their resident foraging pastures. *NOAA Technical Memorandum NMFSC-SWFC* **71,** 1-407.	tech report	survey	171 CM turtles from various islands, 2 with *Che.tes., Pla.hex.* "common" (est. herein at 60%) at all sites, 7 turtles with Ste.mur.	Pacific	Hawaii
15.	Barnard, K.H. (1924). Contributions to the crustacean fauna of South Africa No. 7. Cirripedia. *Annals of the South African Museum* **20,** 1-103.	peer	list	CC and CM turtles, both with *Che.car.* and *Che.tes.*	Atlantic	Africa, S. Africa, Table Bay
16.	Beaumont, E.S., Zárate, P., Zardus, J.D., Dutton, P.H., & Seminoff, J.A. (2007). Epibiont occurrence in Galapagos green turtles (*Chelonia mydas*) at nesting and feeding grounds. 27th Annual Symposium on Sea Turtle Biology and Conservation. Myrtle Beach, South Carolina, USA, 8	tech report	survey	5,121 CM turtles: 913 foraging, 0.2% with *Che.tes.* , 36.5% with *Pla.hex.* , and 48.4% with *Cyl.dar* .; 4,208 nesting 11.7% with *Che.tes.* , 0.16% with *Pla.hex.* , and 10.2% with *Cyl.dar.*	Pacific	Galapagos Islands, Ecuador
17.	Benabib, N. M. (1983). Algunos aspectos de la biología de *Dermochelys coriacea* en el Pacifico Mexicano. México D.F., México, Universidad Nacional Autónoma de México (UNAM), technical thesis.	tech report	survey	Epibionts assessed by body region but not by individual for 82 DC turtles. Barnacles not to species *(Platylepas* on carapace scored herein as *Pla.cor.*)	Pacific	Mexico, Michoacan, Mexiquillo
18.	Biasatti, D. M. (2004). Stable carbon isotopic profiles of sea turtle humeri, implications for ecology and physiology. *Palaeogeography, Palaeoclimatology, Palaeoecology* **206,** 203-216.	peer	record	2 CM turtles, both with *Che.tes.*	Carib.	Costa Rica, Tortuguero
				1 DC turtle with *Pla.cor.*	Atlantic	USA, FL, Juno Beach
19.	Borradaile, L. A. (1903). The Fauna and Geography of the Maldive and Laccadive Archipelagoes: Being the Account of the Work carried on and of the Collections made by an Expedition during the years 1899 and 1900. In, Marine Crustaceans. VII. The Barnacles (Cirripedia). Gardner, J. S. ed. Cambridge, U.K., Cambridge University Press. I: Pp. 440-443.	peer	record	1 CM turtle with *Che.tes* .; 1 EI turtle with *Che.car.*	Indian	Laccadive Sea, Lakshadweep Islands

Ref. No.	total turtles analyzed by species							counts of turtles hosting corresponding species of barnacles																		
	CC	CM	DC	EI	LK	LO	ND	*Che.car.*	*Che.tes.*	*Che.ram.*	*Che.sp.*	*Chl.che.*	*Cal.bjr.*	*Cyl.dar.*	*Cyl.sin.*	*Pla.cor.*	*Pla.dec.*	*Pla.hex.*	*Pla.sp.*	*Ste.mur.*	*Sto.drm.*	*Sto.ele.*	*Sto.pil.*	*Sto.pul.*	*Sto.tra.*	*Sto.sp.*
13.		21						0	0	0		0	0	0	0		0	2		0		0		0	0	
14.		171						0	2	0		0	0	0	0		0	103		7		0		0	0	
15.	+							+	+	0		0	0	0	0		0	0		0		0		0	0	
		+						+	+			0		0	0		0	0		0		0				
16.		913						0	2	0		0	0	442	0		0	333		0		0		0	0	
		4208						0	493	0		0	0	429	0		0	7		0		0		0	0	
17.			82								+					77										
18.		2						0	2	0		0	0	0	0		0	0		0		0		0	0	
			1						0							1					0		0			
19.		1						0	1	0		0	0	0	0		0	0		0		0		0	0	
				1				1	0			0			0		0	0		0		0				

Ref. No.	Reference/Source	Record Type	Data Type	Annotation	Ocean Region	Locale
20.	Boyd, L. and Wood, L. (2019). Epibionts of hawksbill sea turtles in Southeast Florida. Thirty-nineth Annual Symposium on Sea Turtle Biology and Conservation, Feb. 2-8, Charleston, South Carolina, U.S.A.	tech report	survey	21 CM turtles, 38.1% with *Che.tes* .; 229 EI turtles, 82.5% with *Che.car.*	Atlantic	USA, FL
21.	Boyd, L., Wood, L., Zardus, J.D. & Knauer, C. (unpublished) Evidence for epibiont host-selectivity among chelonibia barnacles between hawksbill and green sea turtles. Fortieth Annual Symposium on Sea Turtle Biology and Conservation, Mar. 14-20, 2020, Cartagena, Colombia CANCELLED	tech report	survey	32 CM turtles, 6.3% with *Che.tes* .; 120 EI turtles, 27.5% with *Che.car.*	Indian	Madagascar
22.	Brito-Carrasco, B., Varela, R.G., Rojas-Cañizales, D., Gooch, J., & Harrison, E. (2019). Importance of epibiont registry coupled with the minimum curved carapace length (CCLmin) measurement. 36th Annual Symposium on Sea Turtle Biology and Conservation. J. C. Mangel, A. F. Rees, M. Pajuelo, F. Córdova and N. Acuña. Lima, Peru, *NOAA Technical Memorandum NMFS-SEFSC-734* , **3.**	tech report	record	1,419 CM turtles, 24% with barnacles on carapace, (no species listed, scored herein as *Che.sp.*).	Carib.	Costa Rica, Tortuguero
23.	Brittain, R., Handy, S., & Lucas, S. (2012). Two reports of juvenile hawksbill sea turtles (*Eretmochelys imbricata*) on the southeast coast of Guatemala. *Marine Turtle Newsletter* **133,** 20-22.	peer	record	2 EI turtles, both with *Che.tes.*	Pacific	Guatemala, La Barrona
24.	Broch, H. (1927). Studies on Moroccan cirripeds. *Bulletin de la Société des Sciences Naturelles du Maroc* **7**, 11-38 + 4 plates.	peer	list	1 CC turtle with *Pla.hex.*	Mediter.	Africa, Morocco

Ref. No.	total turtles analyzed by species							counts of turtles hosting corresponding species of barnacles																		
	CC	CM	DC	EI	LK	LO	ND	*Che.car.*	*Che.tes.*	*Che.ram.*	*Che.sp.*	*Chl.che.*	*Cal.bjr.*	*Cyl.dar.*	*Cyl.sin.*	*Pla.cor.*	*Pla.dec.*	*Pla.hex.*	*Pla.sp.*	*Ste.mur.*	*Sto.drm.*	*Sto.ele.*	*Sto.pil.*	*Sto.pul.*	*Sto.tra.*	*Sto.sp.*
20.		21						0	8	0	0	0	0	0	0		0	0		0		0		0	0	
			229					189	0			0			0		0	0		0		0				
21.		32							2	0	0	0	0	0	0		0	0		0		0		0	0	
			120					33	0			0			0		0	0		0		0				
22.		1419						0	0	0	341	0	0	0	0		0	0		0		0		0	0	
23.				2				0	2			0			0		0	0		1		0				
24.	1							0	0			0		0	0		0	1		0		0				

Ref. No.	Reference/Source	Record Type	Data Type	Annotation	Ocean Region	Locale
25.	Brongersma, L.D. (1972). European Atlantic turtles. *Zoologische Verhandelingen* **121,** 1-318, pls. 1-12.	peer	record	522 records of turtles (unknown numbers of turtles in some records) across 724 years, from ca. 1248 to Oct. 29, 1971, most from 1680's to 1971 (290+ yrs). 200 records of DC turtles, both live and dead, most do not mention epbionts, 9 mention stomach contents and/or parasites so barnacles would likely have been mentioned if present, 2 with *Sto.ele.* (scored herein as *Sto.drm*). 194 records of CC turtles, 8 mention epibionts, mostly goose barnacles, only 2 with *Che.car.* and *Pla.hex.* 25 records of LK turtles, 1 mentions unidentified barnacles. 25 records of CM turtles, 1 mentions unidentified barnacles. 1 record of an EI turtle. 77 unidentified turtles.	Atlantic	northeast
26.	Brown, C.H. & Brown, W.M. (1995). Status of sea turtles in the Southeastern Pacific, emphasis on Perú. In, Biology and Conservation of Sea Turtles. K. A. Bjorndal ed. Washington, DC, Smithsonian Institution Press, pp. 235-242.	peer	record	CM turtles with *Che.tes.*	Pacific	southeast
27.	Bugoni, L., Krause, L., Oliveira de Almeida, A., & Angélica de Pádua Bueno, A. (2001). Commensal barnacles of sea turtles in Brazil. *Marine Turtle Newsletter* **94,** 7-9.	peer	survey	9 CC turtles, 1 with *Pla.sp.* , 9 with *Pla.hex* .; 13 CM turtles, 4 with *Che.tes.*	Atlantic	Brazil, Rio Grande do Sul

Ref. No.	total turtles analyzed by species							counts of turtles hosting corresponding species of barnacles																		
	**CC**	**CM**	**DC**	**EI**	**LK**	**LO**	**ND**	***Che.car.***	***Che.tes.***	***Che.ram.***	***Che.sp.***	***Chl.che.***	***Cal.bjr.***	***Cyl.dar.***	***Cyl.sin.***	***Pla.cor.***	***Pla.dec.***	***Pla.hex.***	***Pla.sp.***	***Ste.mur.***	***Sto.drm.***	***Sto.ele.***	***Sto.pil.***	***Sto.pul.***	***Sto.tra.***	***Sto.sp.***
25.			9						0							0					2		0			
	8							2	0			0		0	0		0	2		0		0				
26.		+							+																	
27.	9							0	0			0		0	0		0	9	1	0		0				
		13						0	4	0		0	0	0	0		0	0		0		0		0	0	

Ref. No.	Reference/Source	Record Type	Data Type	Annotation	Ocean Region	Locale
28.	Caine, E.A. (1986). Carapace epibionts of nesting loggerhead sea turtles: Atlantic coast of U.S.A. *Journal of Experimental Marine Biology and Ecology* **95,** 15-26.	peer	survey	138 CC turtles, 2 with *Che.car.* , 125 with *Che.tes.*	Atlantic	USA, southeast
29.	Caldwell, D.E. (1963). Second record of the loggerhead sea turtle, *Caretta caretta gigas* , from the Gulf of California. *Copeia* **1963,** 568-569.	peer	record	1 CC turtle with *Coronula reginae* (listed herein as *Che.tes.*)	Pacific	Gulf of California
30.	Cárdenas-Palomo, N. & Maldonado-Gasca, A. (2005). Epibiontes de tortugas de carey juveniles *Eretmochelys imbricata* en El Santuario de Tortugas Marinas de Rio Lagartos, Yucatán, Mexico. *CICIMAR Oceánides* **20,** 29-35.	peer	survey	37 EI turtles, 33 with *Che.tes.* and 36 with *Pla.sp.*	Gulf of Mexico	Mexico, Yucatan, Rio Lagartos
31.	Carr, A.F., Hirth, H.F., & Ogren, L. (1966). The ecology and migrations of sea turtles, 6 – The hawksbill turtle in the Caribbean Sea. *American Museum Novitates* **2248,** 1-29.	peer	record	EI turtles, no epibionts directly mentioned but photos with *Che.car.* on carapace and *Che.tes.* on plastron (Figs. 1-2, pg. 4 and Fig. 8., pg. 13).	Carib.	Costa Rica, Tortuguero
32.	Carriol, R.-P. and W. Vader (2002). Occurrence of *Stomatolepas elegans* (Cirripedia: Balanomorpha) on a leatherback turtle from Finnmark, northern Norway. *Journal of the Marine Biological Association of the United Kingdom* **82,** 1033-1034.	peer	record	1 DC turtle with *Sto.ele.* (scored herein as *Sto.derm.*)	Atlantic	Norway
33.	Casale, P., D'Addario, M., Freggi, D., & Argano, R. (2012). Barnacles (Cirripedia: Thoracica) and associated epibionts from sea turtles in the Central Mediterranean. *Crustaceana* **85,** 533-549.	peer	survey	117 CC turtles, 75% with *Ch.car,* 76% with *Che.tes.* , 50% with *Pla.sp.,* 100% with *Ste.mur.* , and 75% with *Sto.ele* .	Mediter.	Adriatic Sea, Italy, Lampedusa
34.	Chaieb, O., Karaa, S., & Bradai, M.N. (2018). First record of the turtle barnacle *Stephanolepas muricata* (Fischer, 1886) from the Bay of Monastir (eastern coast of Tunisia). *Bulletin de l'Institut National des Sciences et Technologie de la Mer Salammbô, Tunisia* **(numéro spécial)**, 75-77.	tech report	record	1 CC turtle with "numerous" *Steph.mur* . on leading edges of foreflippers.	Mediter.	Tunisia

Ref. No.	total turtles analyzed by species							counts of turtles hosting corresponding species of barnacles																		
	**CC**	**CM**	**DC**	**EI**	**LK**	**LO**	**ND**	***Che.car.***	***Che.tes.***	***Che.ram.***	***Che.sp.***	***Chl.che.***	***Cal.bjr.***	***Cyl.dar.***	***Cyl.sin.***	***Pla.cor.***	***Pla.dec.***	***Pla.hex.***	***Pla.sp.***	***Ste.mur.***	***Sto.drm.***	***Sto.ele.***	***Sto.pil.***	***Sto.pul.***	***Sto.tra.***	***Sto.sp.***
28.	138							2	125			0		0	0		0	0		0		0				
29.	1							0	1			0		0	0		0	0		0		0				
30.				37				0	33			0			0		0	0	36	0		0				
31.				+				+	+			0			0		0	0		0		0				
32.			1						0							0					1		0			
33.	117							88	89					0	0		0	0	59	117		88				
34.	1																			1						

Ref. No.	Reference/Source	Record Type	Data Type	Annotation	Ocean Region	Locale
35.	Chan, B. K. K., Hayashi, R. (2012). Epibiotic barnacles (Crustacea, Cirripedia, Thoracica) collected by the Kumejima 2009 Expedition, with descriptions of two new species. *Zootaxa* **3367,** 21-48.	peer	list	1 CM turtle with 10 *Pla.hex.*	Pacific	Japan, Ryukyu Islands
36.	Chevreux, E. & De Guerne, J. (1893). Crustacés et Cirrhipèdes commensaux des tortues marines de la Méditerranée. *Comptes rendus de l'Académie des sciences, Paris, Sciences de las vie* **116,** 443-445.	peer	list	CC turtles, some with *Pla.hex.*	Mediter.	Balearic and Alboran seas
37.	Cintrón-De Jesús, J. (2001). Barnacles Associated with Marine Vertebrates in Puerto Rico and Florida, University of Puerto Rico, Mayagüez. Thesis.	tech report	record	2 C.m turtles, 2 with *Che.car.,* 2 with *Pl.hex,* 1 with *Sto.drm.* (scored herein as *Sto.ele* .); 4 EI turtles, 2 with *Che.car.* , 4 with *Pla.hex* ., and 1 with *Sto.drm.* (scored herein as *Sto.ele* .)	Carib.	Puerto Rico
38.	Corrales-Gómez, N. & Herrera-Ulloa, Á. (2012). First record of the turtle barnacle *Stephanolepas muricata* from the Pacific coast of Costa Rica. *Marine Turtle Newsletter* **135,** 9-10.	peer	record	1 EI turtle with *Ste.mur.*	Pacific	Costa Rica, Isla Cedros
39.	Corrêa, G.V.V., Ingels, J., Valdes, Y.V., Fonsêca-Genevois, V.G., Farrapeira, C.M.R., & dos Santos, G.A.P. (2014). Diversity and composition of macro- and meiofaunal carapace epibionts of the hawksbill sea turtle (*Eretmochelys imbricata* Linnaeus, 1822) in Atlantic waters. *Marine Biodiversity* **44,** 391-401.	peer	survey	19 EI turtles, 11 with *Che.car.*	Atlantic	Brazil, Pernambuco, Ipojuca,
40.	Covelo, P., Nicolau, L., López, A. (2016). Four new records of stranded Kemp's ridley turtle Lepidochelys kempii in the NW Iberian Peninsula. Marine Biodiversity Records 9, 80.	peer	record	4 LK turtles, some with *Lepas* but no coronuloid barnacles	Atlantic	Spain
41.	Daniel, A. (1955). The Cirripedia of the Madras coast. *Bulletin of the Madras Government Museum, Natural History Section* **6**, 1-40 + 10 pl.	peer	record	CM turtles with *Che.car., Che.tes.* , and *Pla.hex.* ; EI turtles with *Che.tes.*	Indian	Bay of Bengal, Andaman Islands

Ref. No.	total turtles analyzed by species							counts of turtles hosting corresponding species of barnacles																		
	**CC**	**CM**	**DC**	**EI**	**LK**	**LO**	**ND**	***Che.car.***	***Che.tes.***	***Che.ram.***	***Che.sp.***	***Chl.che.***	***Cal.bjr.***	***Cyl.dar.***	***Cyl.sin.***	***Pla.cor.***	***Pla.dec.***	***Pla.hex.***	***Pla.sp.***	***Ste.mur.***	***Sto.drm.***	***Sto.ele.***	***Sto.pil.***	***Sto.pul.***	***Sto.tra.***	***Sto.sp.***
35.		1						0	0	0		0	0	0	0		0	1		0		0		0	0	
36.	+							0	0			0		0	0		0	+		0		0				
37.		2						2	0	0		0	0	0	0		0	2		0		1		0	0	
				4				2	0			0			0		0	4				1				
38.				1				0	0			0			0		0	0		1		0				
39.				19				11	0			0			0		0	0		0		0				
40.					4				0									0				0				
41.		+						+	+	0		0	0	0	0		0	+		0		0		0	0	
				+				0	+			0			0		0	0		0		0				

Ref. No.	Reference/Source	Record Type	Data Type	Annotation	Ocean Region	Locale
42.	Daniel, A. (1962). A new species of platylepadid barnacle (Cirripedia: Crustacea) from the green turtle (Eretmochelys sp.) from little Andaman Island. *Annals of the Magazine of Natural History* **5** (Ser. 13), 641-645.	peer	record	Uncertain turtle species, listed by author as "green turtle (Eretmochelys sp.)", with "Platylepas multidecorata" (syn. *Pla.dec.*).	Indian	Bay of Bengal, India, Chennai
43.	Darwin, C. R. (1854). A Monograph on the Sub-class Cirripedia, with Figures of All the Species. The Balanidae, (or Sessile Cirripedes); the Verrucidae, etc., etc., etc., London, The Ray Society.	peer	record	Unspecified turtles with *Che.car., Che.tes.,* and *Pl.bisex.* (syn. *Pla.hex.)* ; CM turtles with *Pla.dec.*	global	
					Pacific	Galapagos
44.	Davenport, J. (1994). A cleaning association between the oceanic crab *Planes minutus* and the loggerhead sea turtle *Caretta caretta* . *Journal of the Marine Biological Association of the United Kingdom* **74,** 735-737.	peer	record	3 CC turtles, all with *Che.tes.*	Atlantic	Portugal, Madiera
45.	Dawydoff, C. (1952). Contribution a` l'e´tude des inverte´bre´s de la faune marine benthique de l'Indochine. *Bulletin Biologique de la France et de la Belgique* **No. 9**, Supplement 37, 1-158.	peer	list	CM turtles with *Che.tes.* ; EI turtles with *Che.car.*	Indo-Pacific	South China Sea, Southeast Asia
46.	De Loreto, B.O. & Bondioli, A.C.V. (2008). Epibionts associated with green sea turtles *(Chelonia mydas)* from Cananéia, Southeast Brazil. *Marine Turtle* Newsletter **122,** 5-8.	peer	survey	50 juvenile CM turtles, 31 with *Che.tes.* , 26 with *Pla.hex.*	Atlantic	Brazil, Cananéia SE
47.	Deraniyagala, P.E.P. (1939). The tetrapod reptiles of Ceylon. Colombo Museum natural history series. Colombo Museum, Dulau & Co., London, 412 pp.	peer	record	DC turtles with *Che.sp.* ; 3 EI turtles, 2 wtih *Che.car.* on carapace (photos Plate 13, facing page 210 and Plate 14, facing pg. 211), and 1 with *Che.tes.* and *Ste.mur.* (scored herein as Chl.ch.e due to description of boring into plastron).	Indian	Laccadive Sea, Sri Lanka

Ref. No.	total turtles analyzed by species							counts of turtles hosting corresponding species of barnacles																		
	CC	CM	DC	EI	LK	LO	ND	*Che.car.*	*Che.tes.*	*Che.ram.*	*Che.sp.*	*Chl.che.*	*Cal.bjr.*	*Cyl.dar.*	*Cyl.sin.*	*Pla.cor.*	*Pla.dec.*	*Pla.hex.*	*Pla.sp.*	*Ste.mur.*	*Sto.drm.*	*Sto.ele.*	*Sto.pil.*	*Sto.pul.*	*Sto.tra.*	*Sto.sp.*
42.		?		?													+									
43.								+	+									+								
								0	0	0		0	0	0	0		+	0		0		0		0	0	
44.	3							0	3			0		0	0		0	0		0		0				
45.		+						0	+	0		0	0	0	0		0	0		0		0		0	0	
				+				+	0			0			0		0	0		0		0				
46.		50						0	31	0		0	0	0	0		0	26		0		0		0	0	
47.			+								+					0					0					
				3				2	1			1			0		0	0		0		0				

Ref. No.	Reference/Source	Record Type	Data Type	Annotation	Ocean Region	Locale
48.	Detjen, M., Sterling, E., & Gómez, A. (2015). Stable isotopes in barnacles as a tool to understand green sea turtle *(Chelonia mydas)* regional movement patterns. *Biogeosciences Discussions* **12,** 4655-4669.	peer	record	CM turtles with *Pla.sp.*	Pacific	Palmyra Atoll
49.	Devin, M.L., Sadeghi, P. (2010). Barnacles on hawksbill sea turtles, *Eretmochelys imbricata* , in Hormoz Island, Iran (Reptilia, Cheloniidae). *Zoology in the Middle East* **49,** 45-48.	peer	survey	41 EI turtles, all with *Che.car.* and some with *Pla.sp.*	Indian	Arabian Sea, Iran, Hormus Island
50	Díaz, M.A. & Contreras, I.G. (2006). Epibiontes de tortuge golfina *(Lepidochelys olivacea)* en las costas de San José del Cabo, Baja California Sur, Universidad Autónoma de Baja California Sur, Área Interdisciplinaria de Ciencias del Mar, Departamento de Biología Marina.	tech report	survey	28 LO turtles, none with coronuloid barnacles	Pacific	Mexico, Baja
51.	Díaz M., M., Gutiérrez B., J., Jasso L., D., López S., C., Sarti M., L., & Vallejo A., C. (1992). Epibiontes y de las tortugas *Lepidochelys olivacea* y *Dermochelys coriacea* en el playón de Mexiquillo, Michoacán, durante la temporada de anidación 1988-1989. *Publicaciones de la Sociedad Herpetológica Mexicana* **1**, 19-25.	peer	survey	499 DC turtles, 403 with *Pla.hex* . (scored herein as *Pla.cor* .) and *Sto.sp.* (tallied herein as *Sto.drm* .); 59 LO turtles, 39 with *Pl.hex,* and *Sto.sp.* (scored herein as *Sto.ele.*).	Pacific	Mexico, Michoacan
52.	Dobbs, K. A. and A. M. Landry, Jr. (2004). Commensals of nesting hawksbill turtles (Eretmochelys imbricata), Milman Island, northern Great Barrier Reef, Australia. Memoirs of the Queensland Museum 49 part 2, 672.	peer	survey	1,392 EI turtles, 1,123 with *Che.car.* , 30% with *Chl.che.* , and some with *Pla.hex.*	Indo-Pacific	Australia, Milman Island
53.	Dodd, C.K., Jr. (1988). Synopsis of the biological data on the loggerhead sea turtle *Caretta caretta* (Linnaeus 1758). Washington, DC, Fish and Wildlife Service, U.S. Department of the Interior. 88, 110.	tech report	record	CC turtles with *Che.car., Che.tes., Pla.dec., Pla.hex., Ste.mur.,* and *Sto.ele.*	global	

Ref. No.	total turtles analyzed by species							counts of turtles hosting corresponding species of barnacles																		
	CC	CM	DC	EI	LK	LO	ND	*Che.car.*	*Che.tes.*	*Che.ram.*	*Che.sp.*	*Chl.che.*	*Cal.bjr.*	*Cyl.dar.*	*Cyl.sin.*	*Pla.cor.*	*Pla.dec.*	*Pla.hex.*	*Pla.sp.*	*Ste.mur.*	*Sto.drm.*	*Sto.ele.*	*Sto.pil.*	*Sto.pul.*	*Sto.tra.*	*Sto.sp.*
48.		+						0	0	0		0	0	0	0		0	0	+	0		0		0	0	
49.				41				41	0			0			0				+	0		0				
50						28			0					0			0	0		0		0				
51.			499						0							403					403		0			
						59								0			0	39		0		39				
52.				1392				1123	0			418			0		0	+		0		0				
53.	+							+	+			+					+	+	+	+		+				

Ref. No.	Reference/Source	Record Type	Data Type	Annotation	Ocean Region	Locale
54.	Doell, S.A., Connolly, R.M., Limpus, C.J., Pearson, R.M., & van de Merwe, J.P.(2017). Using growth rates to estimate age of the sea turtle barnacle *Chelonibia testudinaria. Marine Biology* **164,** article 222.	peer	survey	CC turtles with *Che.tes.*	Pacific	Australia, Mon Repos
55.	Doğan, A., Bakir, K., & Katağan, T, (2015). The first record of *Chelonibia testudinaria* (Linnaeus, 1758) (Cirripedia: Chelonibiidae) on the Turkish Aegean coast. *Crustaceana* **88,** 1251-1254.	peer	record	CC turtle with *Che.tes.* , photos confirm	Mediter.	Aegean Sea, Izmar Bay, Turkey
56.	Domènech, F., Badillo, F.J., Tomás, J., Raga, J.A., & Aznar, F.J. (2015). Epibiont communities of loggerhead marine turtles *(Caretta caretta)* in the western Mediterranean, influence of geographic and ecological factors. *Journal of the Marine Biological Association of the United Kingdom* **95,** 851-861.	peer	survey	104 CC turtles, 1 with *Che.car.,* 6 with *Che.tes.* , 80 with *Pla.hex.* , 1 wtih *Ste.mur.,* and 1 *with Sto.ele.*	Mediter.	Valencia, Spain
57.	Duron-Dufrenne, M. (1986). Fréquentation de la tortue Luth Dermochelys coriacea L. en Méditerranée Occidentale de juin 1985 à juillet 1986. Mesogée 46, 63-65.	peer	record	8 DC turtles, 1 with *Sto.ele.* barnacles (scored herein as *Sto.drm* .)	Mediter.	Balearic and Alboran seas
58.	Eckert, K.L. & S.A. Eckert (1987). Growth rate and reproductive condition of the barnacle *Conchoderma virgatum* on gravid leatherback sea turtles in Caribbean waters. *Journal of Crustacean Biology* **7**, 682-690.	peer	record	13 DC turtles, some with *Sto.drm.* and *Pla.hex.* (scored herein as *Pl.cor.*)	Carib.	Caribbean Sea, St. Croix
59.	Eckert, K.L. & S.A. Eckert (1988). Pre-reproductive movements of leatherback sea turtles (*Dermochelys coriacea*) nesting in the Caribbean. *Copeia* **1988,** 400-406.	peer	record	83 DC turtles, some with *Pla.hex.* (scored herein as *Pla.cor.*) and *Sto.drm.*	Carib.	Caribbean Sea, St. Croix
60.	Edmondson, C. H. (1946). Reef and Shore Fauna of Hawaii. Honolulu, HI, Bishop Museum, special publication No. 22.	peer	list	CM turtles with *Che.tes.*	Pacific	Hawaii

Ref. No.	total turtles analyzed by species							counts of turtles hosting corresponding species of barnacles																		
	CC	CM	DC	EI	LK	LO	ND	*Che.car.*	*Che.tes.*	*Che.ram.*	*Che.sp.*	*Chl.che.*	*Cal.bjr.*	*Cyl.dar.*	*Cyl.sin.*	*Pla.cor.*	*Pla.dec.*	*Pla.hex.*	*Pla.sp.*	*Ste.mur.*	*Sto.drm.*	*Sto.ele.*	*Sto.pil.*	*Sto.pul.*	*Sto.tra.*	*Sto.sp.*
54.	+							0	+			0					0	0		0		0				
55.	+							0	+			0					0	0		0		0				
56.	104							1	6			0					0	80		1		1				
57.			8						0							0					1		0			
58.			+						0							+					+		0			
59.			+						0							+					+		0			
60.		+						0	+	0		0	0	0	0		0	0		0		0		0	0	

Ref. No.	Reference/Source	Record Type	Data Type	Annotation	Ocean Region	Locale
61.	Enciso-Padilla, I., Almazán-López, D.I., Jaobo-Pérez, F.J., & Jiménez-Piedragil, C.D. (2019). Epibiont diversity in *Lepidochelys olivacea* females on two beaches of the mexican tropical Pacific. 36th Annual Symposium on Sea Turtle Biology and Conservation. J. C. Mangel, A. F. Rees, M. Pajuelo, F. Córdova and N. Acuña. Lima, Peru, NOAA Technical Memorandum NMFS-SEFSC-734, 9-10.	tech report	survey	446 LO turtles, 261 with barnacles (*Che.tes.* and *Sto.ele.*) but prevalence by species not stated	Pacific	Mexico, Jalisco & Guerrero
62.	Ernst, C.H. & Barbour, R.W. (1972). Turtles of the United States. Lexington, KY, University of Kentucky Press.	peer	list	CC turtles with *Che.car., Che.tes., Pla.hex.,* and *Sto.ele.* ; CM turtles with *Che.tes., Pla.hex., Ste.mur.,* and *Stom.el.* ; DC turtles with *Che.tes., Pla.sp.* (scored herein as *Pla.cor.*), and *Sto.ele.* (scored herein as Sto.drm.); EI turtles with *Che.tes.* and *Ste.mur.* ; no barnacle epibionts mentioned for LK turtles; LO turtles with *Sto.ele* .	Atlantic & Pacific	North America, USA
63.	Ewers-Saucedo, C., Hope, N.B., & Wares, J.P. (2016). The unexpected mating system of the androdioecious barnacle *Chelonibia testudinaria* (Linnaeus, 1758). *Molecular Ecology* **25,** 2081–2092.	peer	record	CC turtles with *Che.tes.*	Atlantic	USA, southeast
64.	Ewers-Saucedo, C., Zardus, J.D., & Wares, J.P. (2016). Microsatellite loci discovery from next-generation sequencing data and loci characterization in the epizoic barnacle *Chelonibia testudinaria* (Linnaeus, 1758). *PeerJ* **4**, (e2019).	peer	record	1 CM turtle with *Che.tes.*	Pacific	Australia, Townsville

Ref. No.	total turtles analyzed by species							counts of turtles hosting corresponding species of barnacles																		
	CC	CM	DC	EI	LK	LO	ND	*Che.car.*	*Che.tes.*	*Che.ram.*	*Che.sp.*	*Chl.che.*	*Cal.bjr.*	*Cyl.dar.*	*Cyl.sin.*	*Pla.cor.*	*Pla.dec.*	*Pla.hex.*	*Pla.sp.*	*Ste.mur.*	*Sto.drm.*	*Sto.ele.*	*Sto.pil.*	*Sto.pul.*	*Sto.tra.*	*Sto.sp.*
61.						+			+					0			0	0		0		+				
62.	+							+	+			0					0	+		0		+				
		+						0	+	0		0	0	0	0		0	+		+		+		0	0	
			+						+							+					+		0			
				+				0	+			0			0		0	0		+		0				
					+				0									0				0				
						+			0					0			0	0		0		+				
63.	+							0	+			0		0	0		0	0		0		0				
64.		1						0	1	0		0	0	0	0		0	0		0	0	0		0	0	

Ref. No.	Reference/Source	Record Type	Data Type	Annotation	Ocean Region	Locale
65.	Ewers-Saucedo, C., Chan, B.K.K., Zardus, J.D., & Wares, J.P. (2017). Parallel patterns of host-specific morphology and genetic admixture in sister lineages of a commensal barnacle. *The Biological Bulletin* **232,** 171-185.	peer	record	CC turtles with *Che.tes.*	Atlantic	USA, southeast
66.	Farrapeira, CMR. (2010). Shallow water Cirripedia of the northeastern coast of Brazil: The impact of life history and invasion on biogeography. *Journal of Experimental Marine Biology and Ecology* **392,** 210-219.	peer	list	CC turtles with *Che.car.* and *Che.tes.* ; CM turtles with *Che.tes.*	Atlantic	Brazil
67.	Farrapeira-Assunção, C.M. (1991). Revisão do gênero Chelonibia Leach, 1817 na costa Brasileira (Crustacea, Cirripedia). XVIII Congresso Brasileiro de Zoologia. Salvador, Brazil, 133.	tech report	record	CC turtles with *Che.car.* and *Che.tes.*	Atlantic	Brazil
68.	Fernandez, I., Retamal, M.A. , Mansilla, M., Yáñez, F., Campos, V., Smith, C., Puentes, G., Valenzuela, A., & González, H. (2015). Analysis of epibiont data in relation with the debilitated turtle syndrome of sea turtles in *Chelonia mydas* and *Lepidochelys olivacea* from Concepcion coast, Chile. *Latin American Journal of Aquatic Research* **43,** 1024-1029.	peer	record	1 CM turtle with *Che.tes.*	Pacific	Chile, Concepcion
69.	Fernando, S.A. (2006). Monograph on Indian Barnacles. OSTC Marine Benthos series, vol. 2, Ocean Science & Technology Cell, Cochin University of Science & Technology, Kochi, India.	peer	list	CM turtles with *Che.tes.* and *Pla.hex.* ; LO turtles with *Sto.prae.* (syn. *Sto.ele.*)	Indian	
70.	Fischer, P. (1884). Cirrhipedes de l'archipel de la Nouvelle-Caladonie. *Bulletin de la Société Zoologique de France* , **9**, 355-360.	peer	record	unspecified turtle with *Che.tes.*	Indo-Pacific	Vietnam, Condor Island
71.	Fischer, P. (1886). Description d'un nouveau genre de cirrhipèdes (*Stephanolepas*). Parasite des tortues marines. *Actes de Société Linnéenne Bordeaux* **10,** 193-196.	peer	list	1 EI turtle with *Ste.mur.*	Pacific	New Caledonia

Ref. No.	total turtles analyzed by species							counts of turtles hosting corresponding species of barnacles																		
	CC	CM	DC	EI	LK	LO	ND	*Che.car.*	*Che.tes.*	*Che.ram.*	*Che.sp.*	*Chl.che.*	*Cal.bjr.*	*Cyl.dar.*	*Cyl.sin.*	*Pla.cor.*	*Pla.dec.*	*Pla.hex.*	*Pla.sp.*	*Ste.mur.*	*Sto.drm.*	*Sto.ele.*	*Sto.pil.*	*Sto.pul.*	*Sto.tra.*	*Sto.sp.*
65.	+							0	+			0		0	0		0	0		0		0				
66.	+							+	+			0		0	0		0	0		0		0				
		+						0	+	0		0	0	0	0		0	0		0	0	0		0	0	
67.	+							+	+			0		0	0		0	0		0		0				
68.		1						0	1	0		0	0	0	0		0	0		0	0	0		0	0	
69.		+						0	+	0		0	0	0	0		0	+		0	0	0		0	0	
						+			0					0			0	0		0		+				
70.									+																	
71.				1				0	0			0					0	0		1		0				

Ref. No.	Reference/Source	Record Type	Data Type	Annotation	Ocean Region	Locale
72.	Flint, M., Morton, J.M., Limpus, C.J., Patterson-Kane, J.C., Murray, P.J., & Mills, P.C. (2010). Development and application of biochemical and haematological reference intervals to identify unhealthy green sea turtles (*Chelonia mydas*). *The Veterinary Journal* **185,** 299-304.	peer	survey	178 CM turtles examined for barnacles, carapace and plastron load compared for subsets of healthy and unhealthy hosts resulting in more with *Che.tes.* on plastron than carapace, 83% (value used herein) for healthy hosts.	Pacific	Australia, Moreton Bay
73.	Foster, B.A. (1978). The marine fauna of New Zealand: Barnacles (Cirripedia: Thoracica). *Memoires of the New Zealand oceanographic Institution* **69,** 1-160.	peer	list	EI turtles with *Pla.hex.* ; uncertain host turtles, "Loggerhead, *Lepidochelys olivacea* ", with *Sto.ele* .	Pacific	New Zealand
74.	Frazier, J.G. (1971). Observations on sea turtles at Aldabra Atoll. *Philosophical Transactions of the Royal Society of London. B* **260,** 373-410.	peer	survey	65 CM turtles, 85% with *Che.tes.* and *Pl.hex.* ; 4 EI turtles, 1 with "3 small barnacles"; 1 CC turtle with large *Che.sp.* on the carapace (scored herein as *Che.tes* .) and 300 "small barnacles" on the appendages and plastron (scored herein as *Pla.hex.*).	Indian	Seychelles, Aldabra Atoll

Ref. No.	total turtles analyzed by species							counts of turtles hosting corresponding species of barnacles																		
	**CC**	**CM**	**DC**	**EI**	**LK**	**LO**	**ND**	***Che.car.***	***Che.tes.***	***Che.ram.***	***Che.sp.***	***Chl.che.***	***Cal.bjr.***	***Cyl.dar.***	***Cyl.sin.***	***Pla.cor.***	***Pla.dec.***	***Pla.hex.***	***Pla.sp.***	***Ste.mur.***	***Sto.drm.***	***Sto.ele.***	***Sto.pil.***	***Sto.pul.***	***Sto.tra.***	***Sto.sp.***
72.		178						0	148	0		10	0	0	0		0	0		0	0	0		0	0	
73.				+				0	0			0					0	+		0		0				
	?					?																+				
74.		65						0	55	0		0	0	0	0		0	55		0		0		0	0	
				4				0	0			0					0	0		0		0				
	1							0	1			0		0	0		0	1		0		0				

Ref. No.	Reference/Source	Record Type	Data Type	Annotation	Ocean Region	Locale
75.	Frazier, J. G. (1983). Analisis estadistico de la tortuga golfina *Lepidochelys olivacea* (Eschscholtz) de Oaxaca, Mexico. *Ciencia Pesquera Inst. Nat. Pesca Sria. Pesca Mexico* **4**, 49-75.	peer	survey	100 LO turtles examined, barnacle assessments split between dorsal and ventral but not by individual host and barnacle species not identifed. The higher, ventral counts used herein, calculated from bar graphs Figs. 20 and 30. Barnacles from carapace and plastron assumed herein to be *Chelonibia testudinaria Che.tes.* and barnacles from the skin assumed to be *Sto.ele* .	Pacific	Mexico, Oaxaca
76.	Frazier, J.G. (1989). Observations on stranded green turtles, *Chelonia mydas* , in the Gulf of Kutch. *Journal of the Bombay Natural History Society* **86,** 250-252.	peer	survey	1 CM turtle with *Che.tes.* and *Pla.hex.*	Indian	Arabian Sea, India, Gujarat
77.	Frick, M.G., Williams, K.L., Robinson, M. (1998). Epibionts associated with nesting loggerhead sea turtles (*Caretta caretta*) in Georgia, USA. *Herpetological Review* **29,** 211-214.	peer	survey	65 CC turtles, all with *Che.car.* and *Che.tes.*	Atlantic	USA, GA
78.	Frick, M.G., Slay, C.K. (2000). *Caretta caretta* (Loggerhead Sea Turtle) epizoans. *Herpetological Review* **31,** 102-103.	peer	record	33 CC turtles, all with *Che.tes.*	Atlantic	Canada, Nova Scotia
79.	Frick, M.G., Williams, K.L., Veljacic, D.C., Jackson, J.A., & Knight, S.E. (2002). Epibiont community succession on nesting loggerhead sea turtles, *Caretta caretta,* from Georgia, USA. Proceedings of the twentieth annual symposium on sea turtle biology and conservation. NOAA technical memorandum NMFS-SEFSC-477. A. Mosier, A. Foley and B. Brost, U.S. Department of Commerce, 281-282.	tech report	record	12 CC turtles, all with *Che.tes.*	Atlantic	USA, southeast

Ref. No.	total turtles analyzed by species							counts of turtles hosting corresponding species of barnacles																		
	CC	CM	DC	EI	LK	LO	ND	*Che.car.*	*Che.tes.*	*Che.ram.*	*Che.sp.*	*Chl.che.*	*Cal.bjr.*	*Cyl.dar.*	*Cyl.sin.*	*Pla.cor.*	*Pla.dec.*	*Pla.hex.*	*Pla.sp.*	*Ste.mur.*	*Sto.drm.*	*Sto.ele.*	*Sto.pil.*	*Sto.pul.*	*Sto.tra.*	*Sto.sp.*
75.						87			13													59				
76.		1						0	1	0		0	0	0	0		0	1		0		0		0	0	
77.	65							65	65			0		0	0		0	0		0		0				
78.	33							0	33			0		0	0		0	0		0		0				
79.	12							0	12			0		0	0		0	0		0		0				

Ref. No.	Reference/Source	Record Type	Data Type	Annotation	Ocean Region	Locale
80.	Frick, M.G., Mason, P.A., Williams, K.L., Andrews, K.R., & Gerstung, H. (2003). Epibionts of Hawksbill turtles in a Caribbean nesting ground: a potentially unique association with snapping shrimp (Crustacea: Alpheidae). *Marine Turtle Newsletter* **99,** 8-11.	peer	survey	28 EI turtles, 27 with *Che.car.* and 2 with *Che.tes.*	Carib.	Antigua, Pasture Bay
81.	Frick, M.G., Ross, A., Williams, K.L., Bolten, A.B., Bjorndal, K.A., & Martins, H.R. (2003). Epibiotic associates of oceanic-stage loggerhead turtles from the southeastern North Atlantic. *Marine Turtle Newsletter* **101,** 18-20.	peer	record	17 CC turtles, some with *Che.tes.* and *Che.tes.*	Pacific	Mexico, Baja
82.	Frick, M.G. & Zardus, J.D. (2010). First authentic report of the turtle barnacle *Cylindrolepas darwiniana* since its description in 1916. *Journal of Crustacean Biology* , **30,** 292-295.	peer	record	CC turtles, some with *Cyl.dar.* and *Pla.dec.*	Pacific	Mexico, Sinaloa
83.	Frick, M.G., Zardus, J.D., & Lazo-Wasem, E.A. (2010). A new *Stomatolepas* barnacle species (Cirripedia: Balanomorpha:, Coronuloidea) from leatherback sea turtles. *Bulletin of the Peabody Museum of Natural History* **51,** 123–136.	peer	survey	2 DC turtles with *Sto.pil.*	Atlantic	USA, GA
84.	Frick, M.G., Zardus, J.D., Lazo-Wasem, E.A. (2010). A new coronuloid barnacle subfamily, genus and species from cheloniid sea turtles. *Bulletin of the Peabody Museum of Natural History* **51**, 169–177.	peer	record	5 CM turtles with *Cal.bjr.*	Atlantic	USA, FL-GA
85.	Frick, M.G., Zardus, J.D., Ross, A., Senko, J., Montano-Valdez, D., Bucio-Pacheco, M., & Sosa-Cornejo, I. (2011). Novel records and observations of the barnacle *Stephanolepas muricata* (Cirripedia: Balanomorpha: Coronuloidea); including a case for chemical mediation in turtle and whale barnacles. *Journal of Natural History* **45**, 629-640.	peer	record	CC, CM, and LO turtles with *Ste.mur.*	Atlantic	Panama

Ref. No.	total turtles analyzed by species							counts of turtles hosting corresponding species of barnacles																		
	CC	CM	DC	EI	LK	LO	ND	*Che.car.*	*Che.tes.*	*Che.ram.*	*Che.sp.*	*Chl.che.*	*Cal.bjr.*	*Cyl.dar.*	*Cyl.sin.*	*Pla.cor.*	*Pla.dec.*	*Pla.hex.*	*Pla.sp.*	*Ste.mur.*	*Sto.drm.*	*Sto.ele.*	*Sto.pil.*	*Sto.pul.*	*Sto.tra.*	*Sto.sp.*
80.				28				27	2			0			0		0	0		0		0				
81.	+							+	+			0		0	0		0	0		0		0				
82.	+							0	0			0		+	0		+	0		0		0				
83.			2						0							0					0		2			
84.		5						0	0	0		0	5	0	0		0	0		0		0		0	0	
85.	+							0	0			0		0	0		0	0		+		0				
		+						0	0	0		0	0	0	0		0	0		+		0		0	0	
						+			0			0					0	0		+		0				

Ref. No.	Reference/Source	Record Type	Data Type	Annotation	Ocean Region	Locale
86.	Frick, M.G. (2015). Commensal Barnacles from Leatherback Sea Turtles *(Dermochelys coriacea)* in Nova Scotia, Technical report for the Leatherback Trust.	tech report	record	28 DC turtles; 11 with *Pla.cor.* , 20 with *Sto.ele.* (scored herein as *Sto.drm* .)	Atlantic	USA, FL
87.	Fuller, W.J., Broderick, A.C., Enever, R., Thorne, P., & Godley, B.J. (2010). Motile homes, a comparison of the spatial distribution of epibiont communities on Mediterranean sea turtles. *Journal of Natural History* **44**, 1743-1753.	peer	survey	100 CC turtles, 27 with *Che.car.* and 65 with *Che.tes.* ; 35 CM turtles, 11 with *Che.car.* and 21 with *Che.tes.*	Mediter.	Cyprus, Alagadi beach
88.	García Gallego, I., Lara Uc, M.M., Reséndiz Moreles, E., Lopéz-Vivas, J.M., Barrientos Torres, S., Mota-Rodríguez, C., Fernández-Sanz, H., Moreno-López, G., & Villavicencio-Zúñiga, C. (2019). Identificatión de balanos en tortuga verde Pacifico oriental (*Chelonia mydas*) y tortuga amarilla (*Caretta caretta*) en Baja California sur. El Uso del Conocimiento de las Tortugas Marinas como herramienta para la restauración de sus poblaciones y hábitats asociados México. Carmen, México, Universidad autónoma del Carmen, 205-220.	peer	survey	56 CC turtles 33.3% with *Che.tes.* , 14.91% with *Pla.hex.* , 3.33% with *Ste.mur.* , 0.5% with *Sto.ele* . (some percentage incorrectly calculated, 3 turtles scored herein with *Sto.ele* .); 117 CM turtles, 16.6% with *Che.tes.* , 14.6% with *Pla.hex.* , and 0.83% with *Ste.mur.*	Pacific	Baja California, Mexico
89	Gauld, D.T. (1957). An annotated check-list of the Crustacea of the Gold Coast. I. Cirripedia. *Journal of the West African Science Association* **3**, 10-11.	peer	list	1 CM turtle with *Che.tes.*	Pacific	Baja California, Mexico
90.	Geldiay, R., Koray, T., & Balik, S. (1982). Status of sea turtle populations (*Caretta caretta* and *Chelonia mydas*) in the northern Mediterranean Sea, Turkey. Biology and Conservation of Sea Turtles. K. A. Bjorndal ed. Washington, DC, Smithsonian Institution Press, 425-437.	peer	record	CC turtles with *Che.sp.*	Atlantic	Africa, Ghana
91.	Glazebrook, J.S. & Campbell, R.S.F. (1990). A survey of the diseases of marine turtles in northern Australia. II. Oceanarium-reared and wild turtles. *Diseases of Aquatic Organisms* **9**, 97-104.	peer	survey	21 wild CM turtles, 7 with *Che.sp.* and 3 with *Pla.sp.*	Pacific	Australia, Torres Strait
				1 wild EI turtle with *Che.sp.* and *Pla.sp.*	Indo-Pacific	Australia, Townsville, Nelly Bay

Ref. No.	total turtles analyzed by species							counts of turtles hosting corresponding species of barnacles																		
	CC	CM	DC	EI	LK	LO	ND	*Che.car.*	*Che.tes.*	*Che.ram.*	*Che.sp.*	*Chl.che.*	*Cal.bjr.*	*Cyl.dar.*	*Cyl.sin.*	*Pla.cor.*	*Pla.dec.*	*Pla.hex.*	*Pla.sp.*	*Ste.mur.*	*Sto.drm.*	*Sto.ele.*	*Sto.pil.*	*Sto.pul.*	*Sto.tra.*	*Sto.sp.*
86.			28						0							11					20		0			
87.	100							27	65			0		0	0		0	0		0		0				
		35						11	21	0		0	0	0	0		0	0		0		0		0	0	
88.	56							0	19			0		0	0		0	8		2		3				
		117						0	19	0		0	0	0	0		0	17		1		0				
89		1						0	1	0		0	0	0	0		0	0		0		0		0	0	
90.	+							0	0		+	0		0	0		0	0		0		0				
91.		21									7	0	0	0	0		0	0	3	0		0		0	0	
				1							1	0			0				1	0		0				

Ref. No.	Reference/Source	Record Type	Data Type	Annotation	Ocean Region	Locale
92.	Gönülal, O. (2016). New distributional record of *Chelonibia testudinaria* (Linnaeus, 1758) from the island of Gökçeada, northern Aegean Sea, Turkey. *Turkish Journal of Zoology* **40,** 125-128.	peer	record	2 CC turtles both with *Che.tes.*	Mediter.	Aegean Sea, Turkey
93.	Goodman, M.A., Braun-McNeill, J., Avens, L., & Goshe, L. (2010). Epibiont collection from sea turtles in the estuarine waters of North Carolina. Twenty-Eighth Annual Symposium on Sea Turtle Biology and Conservation. Loreto, Baja California Sur, México, *NOAA Technical Memorandum NMFS-SEFSC-602* , 41.	tech report	record	CC turtles most common of several turtle species, with *Che.tes.* and *Pla.sp.*	Atlantic	North Carolina, Pamlico Sound
94.	Gramentz, D. (1988). Prevalent epibiont sites on *Caretta caretta* in the Mediterranean Sea. *Naturalista Siciliana* **12,** 33-46.	peer	survey	107 CC turtles, 2 with *Che.car.* and 6 with *Che.tes.*	Mediter.	Malta, Greece, Italy
95.	Green, D. (1998). Epizoites of Galapagos green turtles. Proceedings of the sixteenth annual symposium on sea turtle biology and conservation. R. Byles and Y. Fernandez, eds.. Hilton Head, SC, National Marine Fisheries Service. NOAA Technical Memorandum NMFS-SEFSC-412, 63.	tech report	survey	"Almost 7,000 CM turtles": 532 on feeding grounds, 91% with *Cyl.dar.* , 78% with *Pla.dec.* , and 11% with *Che.tes.* ; nesting females (count not given), 68% with *Cyl.dar.* , 60% with *Pla.dec.,* and 21% with *Che.tes.* (6,435 nesting females estimated herein).	Pacific	Galapagos Islands, Ecuador
96.	Guess, R.C. (1982). Occurrence of a Pacific loggerhead turtle, *Caretta caretta gigas* Deraniyagala, in the waters off Santa Cruz Island, California. *California Fish & Game* **68,** 122-123.	peer	record	1 CC turtle with *Che.tes.*	Pacific	USA, CA, Channel Islands
97.	Haelters, J. & Kerckhof, F. (1999). Een waarneming van de lederschildpad *Dermochelys coriacea* (Linnaeus, 1758), en de eerste waarneming van *Stomatolepas dermochelys* Monroe and Limpus, 1979 aan de Belgische kust. *De Strandvlo* **19,** 30-39.	peer	record	1 DC turtle with *Sto.drm.*	Atlantic	North Sea, Belgium

Ref. No.	total turtles analyzed by species							counts of turtles hosting corresponding species of barnacles																		
	CC	CM	DC	EI	LK	LO	ND	*Che.car.*	*Che.tes.*	*Che.ram.*	*Che.sp.*	*Chl.che.*	*Cal.bjr.*	*Cyl.dar.*	*Cyl.sin.*	*Pla.cor.*	*Pla.dec.*	*Pla.hex.*	*Pla.sp.*	*Ste.mur.*	*Sto.drm.*	*Sto.ele.*	*Sto.pil.*	*Sto.pul.*	*Sto.tra.*	*Sto.sp.*
92.	2							0	2			0		0	0		0	0		0		0				
93.	+							0	+			0		0	0				+	0		0				
94.	107							2	6			0		0	0		0	0		0		0				
95.		532						0	59	0		0	0	484	0		415	0		0		0		0	0	
		6435						0	1351	0		0	0	4376	0		3861	0		0		0		0	0	
96.	1							0	1			0		0	0		0	0		0		0				
97.			1						0							0					1		0			

Ref. No.	Reference/Source	Record Type	Data Type	Annotation	Ocean Region	Locale
98.	Hayashi, R. & Tsuji, K. (2008). Spatial distribution of turtle barnacles on the green sea turtle, *Chelonia mydas* . *Ecological Research* **23,** 121–125.	peer	survey	47 CM turtles, 39 with *Che.tes.* , 36 with *Sto.ele.* , 37 with *Pla.sp.*	Pacific	Japan
99.	Hayashi, R. (2009). New host records of the turtle barnacle, *Cylindrolepas sinica* , a case study of sea turtles' behaviour and their epibionts. *Marine Biodiversity Records* **2,** 1-4.	peer	survey	190 CC turtles, 1 with *Cyl.sin.*	Pacific	Japan, Okinawa
100.	Hayashi, R., Takuma, S., Narazaki, T., & Sato, K. (2011). *Chelonia mydas agassizii* (Black [Pacific] sea turtle). Epibiont barnacles. *Herpetological Review* **42,** 264-265.	peer	record	1 CM turtle with *Pla.hex.*	Pacific	Japan
101.	Hayashi, R. (2012). Atlas of the barnacles on marine vertebrates in Japanese waters including taxonomic review of superfamily Coronuloidea (Cirripedia, Thoracica). *Journal of the Marine Biological Association of the United Kingdom* **92,** 107-127.	peer	list	CC turtles with *Che.car., Che.tes., Chl.che., Cyl.dar., Cyl.sin., Pla.hex., Sto.ele.,* and *Ste.mur* .; CM turtles with *Che.tes., Chl.che., Cyl.sin., Pla.dec., Pla.hex., Sto.trn.,* and *Ste.mur.* ; EI turtles with *Che.car., Cyl.sin., Pla.dec., Pla.hex.,* and *Ste.mur.*	Pacific	Japan
102.	Hayashi, R. (2013). Intraspecific variation in the turtle barnacle, *Cylindrolepas sinica* Ren, 1980 (Cirripedia: Thoracica: Coronuloidea), with brief notes on habitat selectivity. *ZooKeys* **327,** 35-42.	peer	survey	1 CM turtle with *Cyl.sin.*	Pacific	Japan, Iwate, Miyako
103.	Hendrickson, J.R. (1958). The green turtle, *Chelonia mydas* (Linn.) in Malaya and Sarawak. *Proceedings of the Zoological Society of London* **130,** 455-535.	peer	survey	2,720 CM turtles, approximately 1/3 with *Che.tes.* and some with *Ste.mur.* (misidentified, scored herein as *Chl.che.*)	Indo-Pacific	Borneo, Sarawak

Ref. No.	total turtles analyzed by species							counts of turtles hosting corresponding species of barnacles																		
	CC	CM	DC	EI	LK	LO	ND	***Che.car.***	***Che.tes.***	***Che.ram.***	***Che.sp.***	***Chl.che.***	***Cal.bjr.***	***Cyl.dar.***	***Cyl.sin.***	***Pla.cor.***	***Pla.dec.***	***Pla.hex.***	***Pla.sp.***	***Ste.mur.***	***Sto.drm.***	***Sto.ele.***	***Sto.pil.***	***Sto.pul.***	***Sto.tra.***	***Sto.sp.***
98.		47						0	39	0		0	0	0	0				37	0		36		0	0	
99.	190								+						1			+		+						
100.		1						0	0	0		0	0	0	0		0	1		0		0		0	0	
101.	+							+	+			+		+	+		0	+		+		+				
		+						0	+	0		+	0	0	+		+	+		+		0		0	+	
				+				+	0			0			+		+	+		+		0				
102.		1						0	0	0		0	0	0	1		0	0		0		0		0	0	
103.		2720						0	907	0		+	0	0	0		0	0		0		0		0	0	

Ref. No.	Reference/Source	Record Type	Data Type	AnnotatIon	Ocean Region	Locale
104.	Hernández-Vázquez, S. & Valadez-González, C. (1998). Observations of the epizoa found on the turtle *Lepidochelys olivacea* at La Gloria, Jalisco, Mexico. *Ciencias Marinas* **24,** 119-125.	peer	survey	46 LO turtles, some with *Che.tes.*	Pacific	Mexico, Jalisco, La Gloria
105.	Hiro, F. (1936). Occurrence of the cirriped *Stomatolepas elegans* on a loggerhead turtle found at Seto. *Annotationes Zoologicae Japonenses* **15,** 312-320.	peer	record	1 CC turtle with *Che.tes., Pla.hex.,* and *Sto.ele.* ; CM turtles with *Pla.hex.*	Pacific	Japan, Honshu, Wakayama
106.	Hiro, F. (1937a). Studies on the Cirripedian fauna of Japan. II. Cirripeds found in the vicinity of the Seto Marine Biological Laboratory. *Memoirs of the College of Science, Kyoto University, series B* **12,** 385-478.	peer	list	CC turtles with *Che.tes., Pla.hex.,* and *Sto.ele.* ; 1 CM turtle with *Pla.hex.*	Pacific	Japan, Honshu, Wakayama, Senri Beach
107.	Hiro, F. (1937b). Cirripeds of the Palao Islands. *Palao Tropical Biological Station Studies* **1,** 37-72.	peer	list	1 CM turtle with *Che.tes.* ; 1 EI turtle with *Che.car.* and *Che.tes.*	Pacific	western Pacific, Palau
108.	Holthuis, L.B. (1952). Enige interessante met drijvende voorwerpen op de Nederlandse kust aangespoelde Zeepissebedden en Zeepokken. *Levende Natuur* **55,** 72-77.	peer	record	1 CC turtle with *Che.car.* and *Pla.hex.*	Atlantic	North Sea, Netherlands
109.	Houle, S. (2014). A preliminary morphometric analysis of diagnostic characters in three geographic clades of *Stomatolepas* (Cirripedia, Balanomorpha, Coronuloidea). MA Thesis, Department of Biological and Environmental Science, Western Connecticut State University, Danbury, CT, USA.	tech report	record	71 *Sto.ele. and Sto.sp* . barnacles: 20 from CM turtles, 22 from CC and LK turtles, and 29 from LO turtles	Atlantic	USA, South Carolina
					Pacific	USA, Hawaii, Maui
					Atlantic	USA, South Carolina
					Pacific	Mexico, Jalisco, La Gloria
110.	Hubbs, C.L. (1977). First record of mating of ridley turtles in California, with notes on commensals, characters, and systematics. *California Fish and Game* **63,** 263-267.	peer	record	1 LO turtle with *Cyl.dar.*	Pacific	USA, CA, La Jolla

Ref. No.	total turtles analyzed by species							counts of turtles hosting corresponding species of barnacles																		
	CC	CM	DC	EI	LK	LO	ND	*Che.car.*	*Che.tes.*	*Che.ram.*	*Che.sp.*	*Chl.che.*	*Cal.bjr.*	*Cyl.dar.*	*Cyl.sin.*	*Pla.cor.*	*Pla.dec.*	*Pla.hex.*	*Pla.sp.*	*Ste.mur.*	*Sto.drm.*	*Sto.ele.*	*Sto.pil.*	*Sto.pul.*	*Sto.tra.*	*Sto.sp.*
104.						+			+					0			0	0		0		0				
105.	1							0	1			0		0	0		0	1		0		1				
		+						0	0	0		0	0	0	0		0	+		0		0		0	0	
106.	+							0	+			0		0	0		0	+		0		+				
		1						0	0	0		0	0	0	0		0	1		0		0		0	0	
107.		1						0	1	0		0	0	0	0		0	0		0		0		0	0	
				1				1	1			0			0		0	0		0		0				
108.	1							1	0			0		0	0		0	1		0		0				
109.	+							0	0			0		0	0		0	0		0		+				
		+						0	0	0		0	0	0	0		0	0		0						+
					+				0									0				+				
						+			0			0		0			0	0		0		+				
110.						1			0			0		1			0	0		0		0				

Ref. No.	Reference/Source	Record Type	Data Type	Annotation	Ocean Region	Locale
111.	Hughes, G.R. (1971). Preliminary report on the sea turtles and dugongs of Mozambique. *Veterinária Moçambicana* **4,** 45-62.	peer	record	CC turtle with *Che.tes.* visible in photo.	Indian	Africa, Moxambique
112.	Hughes, G.R. (1974). The sea turtles of South-East Africa. II. The biology of the Tongaland loggerhead turtle, *Caretta caretta* L. Oceanographic Research Institute, South African Association for Marine Biological Research Durban, South Africa, Oceanographic Research Institute. Investigational Report 36, 1-96.	tech report	survey	74 CC turtles, 36 with *Che.sp.* , some with *Ste.mur.* "burrowed in carapace" (scored herein as *Chl.che.*), and some with *Pla.sp.* ; 343 CM turtles, 25 either with *Che.sp.* or with *Ste.mur.* "burrowed in carapace" (scored herein as *Chl.che.*); .DC turtles with *Pla.sp.* scored herein as *Pla.cor.*)	Indian	Africa, Mozambique channel, Europa Island
113.	Hunt, T.L. (1995). Preliminary survey of commensals associated with *Caretta caretta* . Proceedings of the Twelfth Annual Workshop on Sea Turtle Biology and Conservation. NOAA-Technical Memorandum NMFS-SEFSC-361. J. I. Richardson and T. H. Richardson, eds. Jekyll Island, GA, 204-207.	tech report	list	32 CC turtles sampled but only 4 turtles analyzed. *Che.car.* and *Che.tes.* present, but no frequencies given	Carib.	USA, FL, Casey Key
114.	Ihwan, M.Z., Joseph, J., Jaaman, S.A., Wahab, W., & Hassan, M. (2018). Occurrence of epibiont barnacles *Chelonibia testudinaria* on green turtle *Chelonia mydas* at Brunei Bay. *International Journal of Zoological Research* **14,** 43-48.	peer	survey	5 CM turtles, all with *Che.tes.*	Indo-Pacific	Brunei, Borneo, Brunei Bay
115.	Jones, D.S., Hewitt, M.A., & Sampay, A. (2000). A checklist of the Cirripedia of the South China Sea. *Raffles Bulletin of Zoology* **48** (Supplement No. 8), 233-307.	peer	list	CM and EI turtles with *Che.car.* ; unspecified sea turtles with *Pla. dec., Pl.hex, Sto.ele., Cyl.sin.,* and *Ste.mur.*	Indian	Australia
116.	Jones, D.S. (2003). The biogeography of Western Australian shallow-water barnacles. In, The Marine Flora and Fauna of Dampier, Western Australia. Wells, F.E., Walker, D.I., & Jones. D. S. (eds.). Perth, Western Australian Museum. Pp. 479-496.	peer	list	unspecified sea turtles with *Che.car., Che.tes., Pla.dec., Pla.hex., Sto.ele.,* and *Chl.che.*	Indian	Australia

Ref. No.	total turtles analyzed by species							counts of turtles hosting corresponding species of barnacles																		
	CC	CM	DC	EI	LK	LO	ND	*Che.car.*	*Che.tes.*	*Che.ram.*	*Che.sp.*	*Chl.che.*	*Cal.bjr.*	*Cyl.dar.*	*Cyl.sin.*	*Pla.cor.*	*Pla.dec.*	*Pla.hex.*	*Pla.sp.*	*Ste.mur.*	*Sto.drm.*	*Sto.ele.*	*Sto.pil.*	*Sto.pul.*	*Sto.tra.*	*Sto.sp.*
111.	+							0	+			0		0	0		0	0		0		0				
112.	74										36	+		0	0				+	0		0				
		343						0			12	12	0	0	0		0	0		0		0		0	0	
			+						0							+					0		0			
113.	+							+	+			0		0	0		0	0		0		0				
114.		5						0	5	0		0	0	0	0		0	0		0		0		0	0	
115.		+						+	0	0		0	0	0	0		0	0		0		0		0	0	
				+				+	0			0			0		0	0		0		0				
															+		+	+		+		+				
116.								+	+			+					+	+				+				

Ref. No.	Reference/Source	Record Type	Data Type	Annotation	Ocean Region	Locale
117.	Jones, D.S. (2004). Barnacles (Cirripedia: Thoracica) of the Dampier Archipelago, Western Australia. *Records of the Western Australian Museum* **Supplement No. 66**, 121-157.	peer	list	EI turtles with *Che.car., Che.tes.,* and *Pla.dec.*	Indo-Pacific	Australia
118.	Jones, D.S. (2010). The littoral and shallow-water barnacles (Crustacea: Cirripedia) of south-eastern Queensland. Proceedings of the Thirteenth International Marine Biological Workshop, The Marine Fauna and Flora of Moreton Bay, Queensland. P. J. F. Davie and J. A. Phillips. International Marine Biological Workshop North Stradbroke Island, Qld. 2005, *Memoirs of the Queensland Museum-Nature* **54,** 199-233.	tech report	list	CC, CM, and EI turtles with *Che.car and Che.tes.* ; LO and ND turtles with *Che.tes.*	Indo-Pacific	South China Sea
119.	Karaa, S., Jribi, I., Bouain, A., & Bradai, M.N. (2012). Cirripedia associated with loggerhead sea turtles, *Caretta caretta,* in the Gulf of Gabès, Tunisia. *Cahiers de Biologie Marine* **53,** 169-176.	peer	survey	113 CC turtles, 13 with *Che.car.* , 35 with *Che.tes.* , 18 with *Pla.hex.* , 12 with *Ste.mur.* , and 7 with *Sto.ele.*	Mediter.	Gulf of Gabès, Tunisia
120.	Killingley, J.S. & Lutcavage, M. (1983). Loggerhead turtle movements reconstructed from 18O and 13C profiles from commensal barnacle shells. *Estuarine, Coastal & Shelf Science* **16,** 345-349.	peer	record	6 CC turtles, all with *Che.tes.*	Atlantic	USA, Chesapeake Bay
121.	Kitsos, M.-S., Christodoulou, M., Kalpakis, S., Noidou, M., & Koukouras, A. (2003). Cirripedia Thoracica associated with *Caretta caretta* (Linnaeus, 1758) in the northern Aegean Sea. *Crustaceana* **76,** 403-409.	peer	survey	37 CC turtles, 36 with *Che.tes.* , 4 with *Pla.hex.* , and 4 with *Sto.ele.*	Mediter.	Aegean Sea, Greece
122.	Kitsos, M.-S., Christodoulou, M., Arvantidis, C., Mavidis, M., Kirmitzoglou, I., & Koukouras, A. (2005). Composition of the organismic assemblage associated with *Caretta caretta* . *Journal of the Marine Biological Association of the U.K.* **85,** 257-261.	peer	survey	29 CC turtles, 25 with *Che.tes* . and 3 with *Sto.ele.*	Mediter.	Aegean Sea, Greece

Ref. No.	total turtles analyzed by species							counts of turtles hosting corresponding species of barnacles																		
	CC	CM	DC	EI	LK	LO	ND	*Che.car.*	*Che.tes.*	*Che.ram.*	*Che.sp.*	*Chl.che.*	*Cal.bjr.*	*Cyl.dar.*	*Cyl.sin.*	*Pla.cor.*	*Pla.dec.*	*Pla.hex.*	*Pla.sp.*	*Ste.mur.*	*Sto.drm.*	*Sto.ele.*	*Sto.pil.*	*Sto.pul.*	*Sto.tra.*	*Sto.sp.*
117.				+				+	+			0			0		+	0		0		0				
118.	+							+	+			0		0	0		0	0		0		0				
		+						+	+	0		0	0	0	0		0	0		0		0		0	0	
				+				+	+			0			0		0	0		0		0				
						+			+					0			0	0		0		0				
							+		+			0						0				0				
119.	113							13	35			0		0	0		0	18		12		7				
120.	6							0	6			0		0	0		0	0		0		0				
121.	37							0	36			0		0	0		0	4		0		4				
122.	29							0	25			0		0	0		0	0		0		3				

Ref. No.	Reference/Source	Record Type	Data Type	AnnotatIon	Ocean Region	Locale
123.	Kobayashi, M. (2000). Relationship between brightness or size and the presence of barnacles on the carapace of the hawksbill turtles (*Eretmochelys imbricata*). *Current Herpetol* **19,** 91-96.	peer	survey	2,756 EI turtles, 669 with *Che.sp.*	Carib.	Cuba
124.	Korschelt, E. (1933). Über zwei parasitäre Cirripedien, *Chelonibia* und *Dendrogaster,* nebst Angaben über die Beziehungen der Balanomorphen zu ihrer Unterlage. *Zoologische Jahrbüch. Abteilung für Systematik, Geographie und Biologie der Tiere* **64,** 1-40.	peer	record	1 CM turtle with *Che.ram.*	Atlantic	USA, FL
125.	Koukouras, A. & Matsa, A. (1998). The thoracican cirriped fauna of the Aegean Sea, new information, check list of the Mediterranean species, faunal comparisons. *Senckenbergiana Maritima* **28,** 133-142.	peer	list	CC turtles with *Che.tes.* and *Pla.hex.*	Mediter.	Aegean
126.	Lanfranco, G. (1979). *Stomatolepas elegans* Costa (Crustacea, Cirripedia) on *Dermochelys coriacea* Linn., in Maltese waters. *Central Med. Naturalist* **1,** 24.	peer	record	DC turtle with *Sto.ele.* (scored herein as *Sto.drm.*)	Mediter.	Malta
127.	Lara Uc, M., Trejo Méndez, M.C., Reséndiz Moreles, E., López-Vivas, J.M., Hinojosa Arango, G., Gumeta Gómez, F., Ramos Ávila, D., Lucero Silva, V., & Mota-Rodríguez, C. (2019). Fauna asociada a tortuga amarilla *Caretta caretta* , tortuga verde del Pacifico oriental *Chelonia mydas* , tortuga golfina *Lepidochelys olivacea* en la región península de Baja California y Pacifico Norte. El Uso del Conocimiento de las Tortugas Marinas como herramienta para la restauración de sus poblaciones y hábitats asociados México. Univ. autónoma del Carmen.	tech report	survey	CC turtles with *Che.tes., Stp.mur.* and *Sto.ele* .; CM turtles with *Che.tes., Stp.mur.,* and *Pla.hex..* ; 36 CM turtles, 32% with *Che.tes.* ; LO turtles with *Che.tes., Stp.mur.,* and *Sto.ele* .; 20 LO turtles, 2% with *Che.tes.* (percentage equates to less then 1 turtle, scored herein as 20%).	Pacific	Mexico, Baja
128.	Lauckner, G. (1985). Diseases of Reptilia. Diseases of Marine Animals. O. Kinne, ed. Hamburg, Germany, Biologische Anstalt Helgoland. Volume IV, Part 2 Reptilia, Aves, Mammalia, 551-626.	peer	record	CC turtles with *Che.car., Che.tes.* , CM turtles with *Che.tes.* ; DC turtles with *Pla.hex.* (scored herein as *Pla.cor* .) and *Sto.ele* . (scored herein as *Sto.drm* .); EI turtles with *Che.tes.* and [illegible]	global	

Ref. No.	total turtles analyzed by species							counts of turtles hosting corresponding species of barnacles																		
	CC	CM	DC	EI	LK	LO	ND	*Che.car.*	*Che.tes.*	*Che.ram.*	*Che.sp.*	*Chl.che.*	*Cal.bjr.*	*Cyl.dar.*	*Cyl.sin.*	*Pla.cor.*	*Pla.dec.*	*Pla.hex.*	*Pla.sp.*	*Ste.mur.*	*Sto.drm.*	*Sto.ele.*	*Sto.pil.*	*Sto.pul.*	*Sto.tra.*	*Sto.sp.*
123.				2756							669	0			0		0	0		0		0				
124.		1						0	0	1		0	0	0	0		0	0		0		0		0	0	
125.	+							0	+			0		0	0		0	+		0		0				
126.			1						0							0					1		0			
127.	+							0	+			0		0	0		0	0		+		+				
		36						0	12	0		0	0	0	0		0	+		+		0		0	0	
						20			4					0			0	0		+		+				
128.	+							+	+			0		0	0		0	0		0		0				
		+							+									0				0				
			+						0							+					+		0			
				+				0	+	0		+	0	0	0		0	0		0		0		0	0	

Ref. No.	Reference/Source	Record Type	Data Type	Annotation	Ocean Region	Locale
				Chl.car .; LK turtles with *Che.tes.*		
129	Laurent, L. (1988). Observations pélagiques de la caouanne *Caretta caretta* Linnaeus (Chelonii, Cheloniidae) en Méditerranée occidental. *Bulletin de la Société herpétologique de France* **45,** 9-16.	peer	record	2 CC turtles, 1 with *Pla.hex.*	Mediter.	
130.	Lazo-Wasem, E.A., Pinou, T., Peña de Niz, A., & Feuerstein, A. (2011). Epibionts associated with the nesting marine turtles *Lepidochelys olivacea* and *Chelonia mydas* in Jalisco, Mexico, A review and field guide. *Bulletin of the Peabody Museum of Natural History* **52,** 221–240.	peer	survey	124 LO turtles, 30 with *Che.tes.* , 68 with *Sto.ele.,* and 26 with *Pla.hex.;* 6 CM turtles, 5 with *Che.tes.* , 1 with *Pla.dec* ., 1 with *Pla.hex* ., and 2 with *Sto.ele.*	Pacific	Mexico, Jalisco, Tepoa Beach
131.	Limpus, C.J., Parmenter, C.J., Baker, V., & Fleay, A. (1983). The Crab Island sea turtle rookery in the north-eastern Gulf of Carpentaria. *Australian Wildlife Research* **10,** 173-184.	peer	survey	328 ND turtles, "most" with *Che.tes.* (estimated herein at 80%), 1 with *Chl.che.* , every turtle with *Pla.sp.* , and "several" with *Sto.ele.* (estimted herein at 5).	Indo-Pacific	Australia, Moreton Bay
132.	Limpus, C.J., Miller, J.D., Baker, V., & McLachlan, E. (1983). The hawksbill turtle, *Eretmochelys imbricata* (L.), in north-eastern Australia: the Campbell Island Rookery. *Australian Wildlife Research* **10,** 185-197.	peer	survey	EI turtles with *Che.car.* , *Che.tes.* , *Pla.dec.,* *Pla.hex.,* and *Ste.mur.*	Indo-Pacific	Australia, Campbell Island
133.	Limpus, C.J., Couper, P.J., Read, M.A. (1994). The green turtle, *Chelonia mydas,* in Queensland, population structure in a warm temperate feeding area. *Memoirs of the Queensland Museum* **35,** 139-154.	peer	survey	784 CM turtles, 415 with *Che.tes.* , 2 with *Chl.che.* , and some with *Pla.hex., Ste.mur.* and *Sto.ele.* present.	Indo-Pacific	Australia, Crab Island
134.	Limpus, C.J. & Limpus, D.J. (2003). Biology of the loggerhead turtle in western south Pacfic Ocean foraging areas. Biology and Conservation of the Loggerhead Sea Turtle. Bolten, A.B. & Witherington, B., eds. Washington, DC, Smithsonian Institution Press, 99-113.	peer	record	CC pelagic recruits arrive to coast with *Ste.mur.*	Pacific	Australia

Ref. No.	total turtles analyzed by species							counts of turtles hosting corresponding species of barnacles																			
	CC	CM	DC	EI	LK	LO	ND	*Che.car.*	*Che.tes.*	*Che.ram.*	*Che.sp.*	*Chl.che.*	*Cal.bjr.*	*Cyl.dar.*	*Cyl.sin.*	*Pla.cor.*	*Pla.dec.*	*Pla.hex.*	*Pla.sp.*	*Ste.mur.*	*Sto.drm.*	*Sto.ele.*	*Sto.pil.*	*Sto.pul.*	*Sto.tra.*	*Sto.sp.*
					+				+			0							0			0				
129	2							0	0			0		0	0		0	1		0		0				
130.		6						0	5	0		0	0	0	0		1	1		0		2		0	0	
						124			30					0			0	26		0		68				
131.							328		262			1							328			5				
132.				+				+	+								+	+		+						
133.		784						0	415			2			0		0	+		+		+		0	0	
134.	+																			+						

Ref. No.	Reference/Source	Record Type	Data Type	Annotation	Ocean Region	Locale
135.	Limpus, C.J., Limpus, D.J., Munchow, M., & Barnes, P. (2005). Queensland turtle conservation project: Raine Island turtle study, 2004-2005, *Queensland Government Conservation Technical and Data Report* **2005,** 1-37.	tech report	survey	2,104 CM turtles, 5 with *Chl.che.* ; 3 EI turtles no barnacle data	Indo-Pacific	Australia, Raine Island
136.	Loop, K.A., Miller, J.D., & Limpus, C.J. (1995). Nesting by the hawsbill turtle (*Eretmochelys imbricata*) on Milman Island, Great Barrier Reef, Australia. *Wildlife Research* **22,** 241-251.	peer	survey	365 EI turtles, 341 with *Che.car.* and 57 with *Chl.che.*	Indo-Pacific	Australia, Milman Island
137.	Losey, G.S., Jr., Balazs, G.H., & Privitera, L.A. (1994). Cleaning symbiosis between the wrasse, *Thalassoma duperry* , and the green turtle, *Chelonia mydas* . *Copeia* **3,** 684-690.	peer	record	CM turtles with *Pla.hex.*	Pacific	Hawaii
138.	Loureiro, N.d.S. (2008). Sea turtles in Santiago Island, Cape Verde. Marine Turtle Newsletter 120, 6.	peer	survey	21 CC turtles, none with coronuloid barnacles	Atlantic	Cape Verde, Santiago Island
139.	Loza, A.L. & Lopez-Jurado, L.F. (2008). Comparative study of the epibionts on the pelagic and mature female loggerhead turtles on the Canary and Cape Verde Islands. Proceedings of the Twenty-Fourth Annual Symposium on Sea Turtle Biology and Conservation, San Jose, Costa Rica, NOAA Technical Memorandum.	tech report	record	CC turtles with *Che.tes.*	Atlantic	Cape Verde and Canary Islands
140.	Lutcavage, M. & Musick, J.A. (1985). Aspects of the biology of sea turtles in Virginia. *Copeia* **1985,** 449-456.	peer	survey	60 CC turtles, 25 with *Che.tes.* , 10 with *Pla.hex.* , and 6 with *Sto.prae* . (syn. *Sto.ele* .); 23 LK turtles with *Che.tes., Pla.hex., and Sto.prae.* (syn. *Sto.ele* .).	Atlantic	USA, Chesapeake Bay, VA
-	Lutcavage, M. (1981). The status of marine turtles in Chesapeake Bay and Virginia costal waters, The College of William and Mary. M.S. thesis.			duplicate data (see Lutcavage & Musick, 1985).		

Ref. No.	total turtles analyzed by species							counts of turtles hosting corresponding species of barnacles																		
	CC	CM	DC	EI	LK	LO	ND	*Che.car.*	*Che.tes.*	*Che.ram.*	*Che.sp.*	*Chl.che.*	*Cal.bjr.*	*Cyl.dar.*	*Cyl.sin.*	*Pla.cor.*	*Pla.dec.*	*Pla.hex.*	*Pla.sp.*	*Ste.mur.*	*Sto.drm.*	*Sto.ele.*	*Sto.pil.*	*Sto.pul.*	*Sto.tra.*	*Sto.sp.*
135.		2104						0	0			5			0		0	0		0		0		0	0	
				+				0	0								0	0		0						
136.				365				341	0			57			0		0	0		0						
137.		+						0	0	0		0	0	0	0		0	+		0		0		0	0	
138.	21							0	0			0		0	0		0	0		0		0				
139.	+							0	+			0		0	0		0	0		0		0				
140.	60							0	25			0		0	0		0	10		0		0				
					+				+									+				+				
-																										

Ref. No.	Reference/Source	Record Type	Data Type	Annotation	Ocean Region	Locale
141.	Majewska, R., Santoro, M. Bolaños, F., Chaves, G., & De Stefano, M. (2015). Diatoms and other epibionts associated with olive ridley (*Lepidochelys olivacea*) sea turtles from the Pacific coast of Costa Rica. *PLoS ONE* **10,** e0130351.	peer	survey	55 LO turtles, 43 with *Che.tes.* , 1 with *Pla.dec.* , 1 with *Pla.hex* ., and 47 with *Sto.ele.*	Pacific	Costa Rica, Guanacaste, Ostional
142.	Margaritoulis, D. (1985). Preliminary observations on the breeding behaviour and ecology of *Caretta caretta* in Zakynthos, Greece. 2e Congrès International sur la Zoogéographie et L'Écologie de la Grèce et des Régions Avoisinantes, Athènes, Septembre 1981. Biologica Gallo-Hellenica 10, 323.	peer	record	CC turtles with *Che.tes.*	Mediter.	Greece, Zakynthos
143.	Márquez M., R. (1994). Synopsis of biological data on the Kemp's ridley turtle, *Lepidochelys kempi* (Garman, 1880). NOAA-Technical Memorandum NMFS-SEFSC-343, 1-91.	tech report	record	LO turtles with *Che.tes., Pla.hex.,* and *Sto.ele.*	Gulf of Mexico	
144.	Matsuura, I. & Nakamura, K. (1993). Attachment pattern of the turtle barnacle *Chelonibia testudinaria* on carapace of nesting loggerhead turtle *Caretta caretta. Bulletin of the Japanese Society of Scientific Fisheries* **59,** 1803.	peer	survey	118 CC turtles, some with *Che.car.* and *Che.tes.*	Pacific	Japan, Kyushu
145.	Maulida, F.F., Susilo Hadi, M.A.I., & Reischig, T. (2017). Geometry morphometry and health status of hawksbill turtle (*Eretmochelys imbricata* Linnaeus, 1766) in Maratua Island, East Kalimantan-Indonesia. 4th International Conference on Biological Science, KnE Life Sciences. ICBS Conference Proceedings, 100-110.	tech report	record	11 EI turtles, some with *Che.tes.*	Indo-Pacific	Borneo, Maratua Island
146.	McAllister, H.J., Bass, A.J., &van Schoor, H.J. (1965). Marine turtles on the coast of Tongaland, Natal. *Lammergeyer* **III,** 10-40.	peer	survey	82 CC turtles, about 1/3 with *Che.tes.* (assumed 19 DC turtles mentionedare not included in the author's count).	Indian	Africa, S. Africa, Natal

Ref. No.	total turtles analyzed by species							counts of turtles hosting corresponding species of barnacles																		
	CC	CM	DC	EI	LK	LO	ND	*Che.car.*	*Che.tes.*	*Che.ram.*	*Che.sp.*	*Chl.che.*	*Cal.bjr.*	*Cyl.dar.*	*Cyl.sin.*	*Pla.cor.*	*Pla.dec.*	*Pla.hex.*	*Pla.sp.*	*Ste.mur.*	*Sto.drm.*	*Sto.ele.*	*Sto.pil.*	*Sto.pul.*	*Sto.tra.*	*Sto.sp.*
141.						55			43					0			1	1		0		47				
142.	+								+																	
143.						+			+					0			0	+		0		+				
144.	+							+	+			0		0	0		0	0		0		0				
145.				+				0	+			0			0		0	0		0		0				
146.	82							0	27			0		0	0		0	0		0		0				

Ref. No.	Reference/Source	Record Type	Data Type	Annotation	Ocean Region	Locale
147.	McCann, C. (1969). First southern hemisphere record of the platylepadine barnacle *Stomatolepas elegans* (Costa) and notes on the host *Dermochelys coriacea* (Linné). *New Zealand Journal of Marine and Freshwater Research* **3,** 152-158.	peer	record	1 DC turtle with *Sto.ele.* (scored herein as *Sto.drm* .)	Pacific	New Zealand, North Island
148.	Mifsud, C.R., Schembri, P.J., Rizzo, M., Baldacchino, A.E., Stevens, D.T., & Gruppetta, A. (2009). Preliminary data on the epibions of *Caretta caretta* from Maltese waters. Second Mediterranean Conference on Marine Turtles, Kemer, Antalya, Turkey.	tech report	survey	4 CC turtles, each with *Che.tes.*	Mediter.	Malta
149.	Miranda, L. & Moreno, R.A. (2002). Epibiontes de *Lepidochelys olivacea* (Eschscholtz, 1829) (Reptilia: Testudinata: Cheloniidae) en la región centro sur de Chile. *Revista de Biologia Marina y Oceanografia* **37,** 145-146.	peer	survey	5 LO turtles, none with coronuloid barnacles.	Pacific	Chile, Arauco Province
150.	Mohanty-Hejmadi, P., Behera, M., & Dutta, S.K. (1989). Commensals on the olive ridley sea turtle. *Marine Turtle Newsletter* **45,** 11-13.	peer	survey	50 LO turtles, none with coronuloid barnacles	Indian	India, Orissa
151.	Monod, T. (1931). Crustaces de Syrie. Les État de Syrie, Richesses marines et fluviales Exploitation actuelle - Avenir. A. Gruvel. Paris, Société d'Éditions Géographiques, Maritimes et Coloniales, 397-435.	peer	list	CC turtles with *Che.tes.*	Mediter.	Alexandretta, Syria
152.	Monroe, R. & Limpus, C.J. (1979). Barnacles on turtles in Queensland waters with descriptions of three new species. *Memoirs of the Queensland Museum* **19,** 197-223.	peer	record	CC turtles with *Che.car., Che.tes., Pla.dec., Pla.hex., Ste.mur.,* and *Sto.ele.* ; CM turtles with *Che.tes., , Pla.dec., Pla.hex., Ste.mur.,* and *Sto.tra* .; DC with *Pla.cor* .; EI turtles with *Che.car., Che.tes., , Pla.dec., Pla.hex.,* and *Ste.mur* .; ND turtles with *Che.tes.* and *Pla.hex.*	Pacific	Australia, Queensland

Ref. No.	total turtles analyzed by species							counts of turtles hosting corresponding species of barnacles																		
	CC	CM	DC	EI	LK	LO	ND	*Che.car.*	*Che.tes.*	*Che.ram.*	*Che.sp.*	*Chl.che.*	*Cal.bjr.*	*Cyl.dar.*	*Cyl.sin.*	*Pla.cor.*	*Pla.dec.*	*Pla.hex.*	*Pla.sp.*	*Ste.mur.*	*Sto.drm.*	*Sto.ele.*	*Sto.pil.*	*Sto.pul.*	*Sto.tra.*	*Sto.sp.*
147.			1						0							0					1		0			
148.	4							0	4			0		0	0		0	0		0		0				
149.						5			0					0			0	0		0		0				
150.						50			0					0			0	0		0		0				
151.	+							0	+			0		0	0		0	0		0		0				
152.	+							+	+			0		0	0		+	+		+		+				
		+						0	+	0		0	0	0	0		+	+		+		0		0	+	
			+						0							+					0		0			
				+				+	+			0			0		+	+		+		0				
							+		+									+				0				

Ref. No.	Reference/Source	Record Type	Data Type	Annotation	Ocean Region	Locale
153.	Monroe, R. (1981). Studies in the Coronulidae (Cirripedia): shell morphology, growth, and function, and their bearing on subfamily classification. *Memoirs of the Queensland Museum* **20,** 237-251.	peer	list	CC turtles with *Cyl.dar.*	Pacific	Australia, Queensland
154.	Mustaquim, J. & Javed, M. (1993). Occurrence of *Chelonibia testudinaria* (Linnaeus) (Crustacea: Cirripedia) in coastal waters of Pakistan. *Pakistan Journal of Marine Sciences* **2,** 73-75.	peer	survey	1 CM turtle with *Che.tes.*	Indian	Arabian Sea, Pakistan, Karachi
155.	Nájera-Hillman, E., Bass, J.B., & Buckham, S. (2012). Distribution patterns of the barnacle, *Chelonibia testudinaria* , on juvenile green turtles (*Chelonia mydas*) in Bahia Magdalena, Mexico. *Revista Mexicana de Biodiversidad* **83,** 1171-1179.	peer	survey	72 CM turtles, 49 with *Che.tes.* and 2 with *Pla.hex.*	Pacific	Mexico, Baja, Bahia Magdalena
156.	Newman, W.A. & Ross, A. (1976). Revision of the balanomorph barnacles; including a catalog of the species. *Memoirs of the San Diego Society of Natural History* **9,** 1-108.	peer	list	unspecified turtles with *Che.tes., Cyl.dar., Pla.dec., Pla.hex., Sto.ele.,* and *Ste.mur.*	Atlantic<br>Carib.<br>Mediter.<br>Indian<br>Indo-Pacific<br>Pacific	
157.	Niaz Rizvi, S.H. & Moazzam, M. (2006). Sessile barnacles (Cirripedia) from the Pakistan coast. *Pakistan Journal of Marine Sciences* **15,** 91-118.	peer	list	CM turtles with *Che.tes* .; unspecified turtles with *Che.car.*	Indian	Arabian Sea, Pakistan, Karachi
158.	Nilsson-Cantell, C.A. (1930). Diagnoses of some new cirripedes from the Netherlands Indies collected by the expedition of His Royal Highness the Prince Leopold of Belgium in 1929. *Bulletin du Musée royal d'Histoire naturelle de Belgique* **6,** 1-2.	peer	record	CM turtles with *Sto.tra.*	Indian	Sri Lanka, Bentota
159.	Nilsson-Cantell, C.A. (1932). The barnacles *Stephanolepas* and *Chelonibia* from the turtle *Eretmochelys imbricata. Ceylon Journal of Science. Section B, Zoology* **16,** 257-264 pls. 2 figs.	peer	record	E.i turtles with *Ch.tes,* and *Ste.mur.* (misidentified, scored herein as *Chl.che.*)	Indo-Pacific	Indonesia, Aru Islands

Ref. No.	total turtles analyzed by species							counts of turtles hosting corresponding species of barnacles																		
	CC	CM	DC	EI	LK	LO	ND	*Che.car.*	*Che.tes.*	*Che.ram.*	*Che.sp.*	*Chl.che.*	*Cal.bjr.*	*Cyl.dar.*	*Cyl.sin.*	*Pla.cor.*	*Pla.dec.*	*Pla.hex.*	*Pla.sp.*	*Ste.mur.*	*Sto.drm.*	*Sto.ele.*	*Sto.pil.*	*Sto.pul.*	*Sto.tra.*	*Sto.sp.*
153.	+							0	0			0		+	0		0	0		0		0				
154.		1						0	1	0		0	0	0	0		0	0		0		0		0	0	
155.		72						0	49	0		0	0	0	0		0	2		0		0		0	0	
156.								+	+									+				+				
									+					+				+				+				
									+									+				+				
									+									+		+		+				
								+	+								+	+		+		+				
									+								+	+				+				
157.		+						0	+	0		0	0	0	0		0	0		0		0		0	0	
								+																		
158.		1						0	0	0		0	0	0	0		0	0		0		0		0	1	
159.				1				0	1			1			0		0	0		0		0				

Ref. No.	Reference/Source	Record Type	Data Type	Annotation	Ocean Region	Locale
160.	Nilsson-Cantell, C.A. (1937). On a second collection of Indo-Malayan cirripeds from the Raffles Museum. *Bulletin of the Raffles Museum* **13,** 93-96.	peer	list	EI turtles with *Che.tes.*	Indo-Pacific	Singapore
161.	Ooi, S.L. & Palaniappan, P.M. (2011). Silent turtle dwellers: Barnacles on resident green and hawksbill turtles of Mabul and Sipadan Islands. *Borneo Science* **28,** 66-72.	peer	survey	189 CM turtles, 62 with *Che.tes.* (pers. comm.); 7 EI turtles, 2 with *Che.tes.*	Indo-Pacific	Borneo
162.	O'Riordan, C.E. & Holmes, J.M.C. (1978). Marine fauna notes from the National Museum of Ireland. 5. Passengers on the North Atlantic currents. *Irish Naturalists' Journal* **19,** 152-153.	peer	record	DC turtles with *Sto.ele.* (scored herein as *Sto.drm* .)	Atlantic	Ireland
163.	O'Riordan, C.E. (1979). Marine fauna notes from the National Museum of Ireland 6. *Irish Naturalists' Journal* **19,** 356-358.	peer	record	DC turtles with *Pla.hex* . (scored herein as *Pla.cor.*)	Atlantic	Ireland
164.	Pereira, S., Lima, E.H.S.M., Ernesto, L., Mathews, H., & Ventura, A. (2006). Epibionts associated with *Chelonia mydas* from Northern Brazil. *Marine Turtle Newsletter* **111,** 17-18.	peer	survey	32 CM turtles, all with *Che.tes.*	Atlantic	Brazil, Ceará State, Almofala
165.	Pérez, J.d.A. (2001). Estudio descriptivo inicial de los epibiontes de tortuga boba (*Caretta caretta* , Linnaeus, 1758) en el Mediterráneo occidental. Granada, Spain, Universidad de Granada y Instituto Español de Oceanografía, 27.	tech report	survey	670 CC turtles, some with balanomorph barnacles (scored herein as *Che.sp* . observed in photo from Fig. 18)	Mediter.	Balearic and Alboran seas
166.	Pfaller, J.B., Bjorndal, K.A., Reich, K.J., Williams, K.L., & Frick, M.G.(2006). Distribution patterns of epibionts on the carapace of loggerhead turtles, *Caretta caretta* . *Biodiversity Records* **5381,** 1-4.	tech report	survey	18 CC turtles, 17 with *Che.sp.*	Atlantic	USA, FL, Brevard Co.
167.	Pfaller, J.B., Frick, M.G., Reich, K.J., Williams, K.L., & Bjorndal, K.A. (2008). Carapace epibionts of loggerhead turtles (*Caretta caretta*) nesting at Canaveral National Seashore, Florida. *Journal of Natural History* **42,** 1095-1102.	peer	survey	52 CC turtles, 47 with *Che.car.* and 52 with *Che.tes.*	Atlantic	USA, GA, Wassaw Island

Ref. No.	total turtles analyzed by species							counts of turtles hosting corresponding species of barnacles																		
	CC	CM	DC	EI	LK	LO	ND	*Che.car.*	*Che.tes.*	*Che.ram.*	*Che.sp.*	*Chl.che.*	*Cal.bjr.*	*Cyl.dar.*	*Cyl.sin.*	*Pla.cor.*	*Pla.dec.*	*Pla.hex.*	*Pla.sp.*	*Ste.mur.*	*Sto.drm.*	*Sto.ele.*	*Sto.pil.*	*Sto.pul.*	*Sto.tra.*	*Sto.sp.*
160.				1				0	1			0			0		0	0		0		0				
161.		189						0	62			0			0		0	0		0		0		0	0	
				7				0	2								0	0		0						
162.			+						0							0					+		0			
163.			+						0							+					0		0			
164.		32						0	32	0		0	0	0	0		0	0		0		0		0	0	
165.	670										214	0		0	0		0	0		0		0				
166.	18										17	0		0	0		0	0		0		0				
167.	52							47	52			0		0	0		0	0		0		0				

Ref. No.	Reference/Source	Record Type	Data Type	Annotation	Ocean Region	Locale
168.	Pilsbry, H.A. (1910). *Stomatolepas,* a barnacle commensal in the throat of the loggerhead turtle. *American Naturalist* **44,** 304-306.	peer	record	CC turtles with *Sto.prae* . (syn. *Sto.ele* .).	Atlantic	
169.	Pilsbry, H.A. (1916). The sessile barnacles (Cirripedia) contained in the collections of the U.S. National Museum; including a monograph of the American species. *Bulletin of the United States National Museum* **93,** 1-366.	peer	list	CC turtles all with *Sto.prae* . (syn. *Sto.ele* .); unspecified turtles with *Che.car., Che.tes., Cyl.dar.,* and *Pla.hex.*	Gulf of Mexico	USA, FL, Tortugas islands
					global	
170.	Pinou, T., Lazo-Wasem, E.A., Dion, K., & Zardus, J.D. (2013). Six degrees of separation in barnacles? Assessing genetic variability in the sea-turtle epibiont Stomatolepas elegans (Costa) among turtles, beaches, and oceans. *Journal of Natural History* **47,** 2193-2212.	peer	record	6 CC turtles with *Sto.ele* .; 2 CM turtles with *Sto.ele* . (scored herein as an unidentified *Sto.sp* .); 1 EI turtle with *Sto.ele* .; 9 LO turtles with *Sto.ele.*	Atlantic	USA, southeast
					Pacific	Hawaii, Maui
					Carib.	Panama, Bocas del Toro
					Pacific	Mexico, Jalisco, Tepoa Beach
171.	Plotkin, P.T. (1996). Occurrence and diet of juvenile loggerhead sea turtles, *Caretta caretta* , in the northwestern Gulf of Mexico. *Chelonian Conservation and Biology* **2,** 78-80.	peer	record	10 CC turtles, 1 with *Che.tes.*	Gulf of Mexico	USA, TX, Mustang Island
172.	Prazzi, E., Piovano, S. Pessani, D., Comparetto, G., & Giacoma, C. (2009). Preferential position of cirripeds epibiont on specimens of *Caretta caretta* captured in Linosa and Lampedusa waters (Pelagie Islands, Sicily, Italy). Second Mediterranean Conference on Marine Turtles, Kemer, Antalya, Turkey.	tech report	survey	81 CC turtles, 11% with *Che.tes.* , 10% with *Pla.hex* ., and 2% with *Sto.ele.*	Mediter.	Pelagie Islands, Sicily, Italy
173.	Rawson, P.D., Macnamee, R., Frick, M.G., & Williams, K.L. (2003). Phylogeography of the coronulid barnacle, *Chelonibia testudinaria* , from loggerhead sea turtles, *Caretta caretta* . *Molecular Ecology* **12,** 2697-2706.	peer	record	56 CC turtles with *Che.tes.*	Atlantic	USA, FL-GA
					Mediter.	Aegean Sea, Greece
					Pacific	Mexico, Baja
					Pacific	Japan

Ref. No.	total turtles analyzed by species							counts of turtles hosting corresponding species of barnacles																		
	CC	CM	DC	EI	LK	LO	ND	*Che.car.*	*Che.tes.*	*Che.ram.*	*Che.sp.*	*Chl.che.*	*Cal.bjr.*	*Cyl.dar.*	*Cyl.sin.*	*Pla.cor.*	*Pla.dec.*	*Pla.hex.*	*Pla.sp.*	*Ste.mur.*	*Sto.drm.*	*Sto.ele.*	*Sto.pil.*	*Sto.pul.*	*Sto.tra.*	*Sto.sp.*
168.	+							0	0			0		0	0		0	0		0		+				
169.	+							0	0			0		0	0		0	0		0		+				
								+	+					+				+								
170.	6							0	0			0		0	0		0	0		0		6				
		2						0	0	0		0	0	0	0		0	0		0						2
				1				0	0			0			0		0	0		0		1				
						9			0					0			0	0		0		9				
171.	10							0	1			0		0	0		0	0		0		0				
172.	81							0	9			0		0	0		0	8		0		2				
173.	34							0	34			0		0	0		0	0		0		0				
	9							0	9			0		0	0		0	0		0		0				
	5							0	5			0		0	0		0	0		0		0				
	8							0	8			0		0	0		0	0		0		0				

Ref. No.	Reference/Source	Record Type	Data Type	Annotation	Ocean Region	Locale
174.	Razaghian, H., Esfandabad, B.S., Hesni, M.A., Shoushtari, R.V., Toranjzar, H., & Miller, J. (2019). Distribution patterns of epibiotic barnacles on the Hawksbill turtle, *Eretmochelys imbricata* , nesting in Iran. *Regional Studies in Marine Science* **27,** 1-6.	peer	survey	122 EI turtles, 121 with *Che.tes.*	Indian	Arabian Sea, Persian Gulf, Iran
175.	Rees, E.I.S. & Walker, G. (1977). A record of the turtle barnacle *Chelonobia* in the Irish Sea. *Porcupine Newsletter* **5,** 189.	peer	record	1 DC turtle with *Che.tes.*	Atlantic	Irish Sea
176.	Reisser, J.W. (2006). Tartugas marinhas da Ilha do Arvoredo, Reserva Biológica Marinha do Arvoredo, SC. Rio Grande, Rio Grande do Sul, Brazil, Fundação Universidade Federal do Rio Grande (FURG).	tech report	record	CM turtles with *Che.tes.* and *Pla.sp.*	Atlantic	Brazil, Santa Catarina
177.	Relini, G. (1980). Guide per il riconoscimento delle specie animali delle ascue lagunari e costiere Italiane. 2. Cirripedi Toracici. Rome, Consiglio Nazionale delle Ricerche, 1-116.	peer	list	Unspecified turtles with *Che.car., Che.tes., Pla.hex.,* and *Sto.ele.*	Mediter.	Italy
178.	Ren, X. (1980). Turtle barnacles of the Xisha Islands, Guangdong Province, China. *Studia Marina Sinica* **17,** 187-197.	peer	list	CC turtles with *Che.tes* .; CM turtles with *Che.tes., Cyl.sin., Pla.dec., Pl,hex.,* and *Sto.pul.*	Indo-Pacific	South China Sea
179.	Ren, X. (1987). Studies on Chinese Cirripedia (Crustacea) VIII. Supplementary Report. *Studia Marina Sinica* **28,** 175-187.	peer	list	CM turtles with *Sto.ele.*	Indo-Pacific	South China Sea, China, Xisha Islands
180.	Richards, H.G. (1930). Notes on barnacles from Cape May County, New Jersey. *Proceedings of the National Academy of Sciences U.S.A.* **82,** 143-144.	peer	record	1 CC turtle with *Che.tes* .; 1 CM turtle with *Pla.hex.*	Atlantic	USA, New Jersey
181.	Ritchie, J. (1924). Turtle barnacles in Scottish waters. *Scottish Nature* **149,** 166.	peer	record	1 CC turtle with *Che.car.*	Atlantic	Scotland, Isle of Skye

Ref. No.	total turtles analyzed by species							counts of turtles hosting corresponding species of barnacles																		
	CC	CM	DC	EI	LK	LO	ND	*Che.car.*	*Che.tes.*	*Che.ram.*	*Che.sp.*	*Chl.che.*	*Cal.bjr.*	*Cyl.dar.*	*Cyl.sin.*	*Pla.cor.*	*Pla.dec.*	*Pla.hex.*	*Pla.sp.*	*Ste.mur.*	*Sto.drm.*	*Sto.ele.*	*Sto.pil.*	*Sto.pul.*	*Sto.tra.*	*Sto.sp.*
174.				122				0	121			0			0		0	0		0		0				
175.			1						1							0					0		0			
176.		+						0	+	0		0	0	0	0		0	+		0		0		0	0	
177.								+	+									+				+				
178.	+							0	+			0		0	0		0	0		0		0				
		2						0	+	0		0	0	0	2		+	+		0		0		1	0	
179.		+						0	0	0		0	0	0	0		0	0		0		+		0	0	
180.	1							0	1			0		0	0		0	0		0		0				
		1						0	0	0		0	0	0	0		0	1		0		0		0	0	
181.	1							1	0			0		0	0		0	0		0		0				

Ref. No.	Reference/Source	Record Type	Data Type	Annotation	Ocean Region	Locale
182.	Robinson, N.J., Gatto, C., Figgener, C., Lazo-Wasem, E.A., Paladino, F.V., Tomillo, P.S., Zardus, J.D., & Pinou, T. (2017). Assessing potential limitations when characterising the epibiota of marine megafauna: effect of gender, sampling location, and inter-annual variation on the epibiont communities of olive ridley sea turtles. *Journal of Experimental Marine Biology and Ecology* **497,** 71–77.	peer	survey	81 LO turtles, 28 with *Che.tes,* 23 with *Pla.hex.* , and 61 with *Sto.ele.*	Pacific	Costa Rica, Guanacaste, Ostional
183.	Robinson, N.J., Lazo-Wasem, E.A., Paladino, F.V., Zardus, J.D., & Pinou, T. (2017). Assortative epibiosis on leatherback, olive ridley, and East Pacific green turtles in the Eastern Tropical Pacific. *Journal of the Marine Biological Association of the United Kingdom* **97,** 1233-1240	peer	survey	6 CM turtles, 6 with *Che.tes* ., 3 with *Pla.hex.* , and 2 wtih *Pla.sp* .; 18 DC turtles, 17 with *Pla.cor.* ; 19 LO turtles, 7 with *Che.tes* ., 5 with *Pla.dec* ., 8 with *Pla.hex* ., and 7 with *Sto.ele.*	Pacific	Costa Rica, Guanacaste, Tamarindo Bay
184.	Robinson, N.J., Lazo-Wasem, E., Butler, B.O., Lazo-Wasem, E.A., Zardus, J.D., & Pinou, T. (2019). Spatial distribution of epibionts on olive ridley sea turtles at Playa Ostional, Costa Rica. *PLoS ONE* **14,** e0218838.	peer	survey	30 LO turtles, 23 with *Che.tes* ., 22 with *Pla.hex* ., and 24 with *Sto.ele.*	Pacific	Costa Rica, Guanacaste, Playa Grande
185.	Ross, A. & Frick, M.G. (2007). From Hendrickson (1958) to Monroe & Limpus (1979) and beyond: An evaluation of the turtle barnacle *Tubicinella cheloniae. Marine Turtle Newsletter* **18,** 2-5.	peer	record		Pacific	Australia, Mon Repos
186.	Rudloe, A., Rudloe, J., & Ogren, L. (1991). Occurrence of immature Kemp's ridley turtles, *Lepidochelys kempi,* in coastal waters of northwest Florida. *Northeast Gulf Science* **12,** 49-53.	peer	survey	106 LK turtles, 25 with *Che.tes.*	Gulf of Mexico	USA, Florida
187.	Sánchez Ruiz, M., García Raso, J E., Baez, J.C., & Camiñas, J.A. (2015). Fauna de epibiontes asociada a ejemplares de tortuga boba (*Caretta caretta* Linnaeus, 1758) capturados de forma accesoria en	tech report	survey	121 CC turtles, none with coronuloid barnacles	Mediter.	Belearic Islands, Spain

Ref. No.	total turtles analyzed by species							counts of turtles hosting corresponding species of barnacles																		
	CC	CM	DC	EI	LK	LO	ND	*Che.car.*	*Che.tes.*	*Che.ram.*	*Che.sp.*	*Chl.che.*	*Cal.bjr.*	*Cyl.dar.*	*Cyl.sin.*	*Pla.cor.*	*Pla.dec.*	*Pla.hex.*	*Pla.sp.*	*Ste.mur.*	*Sto.drm.*	*Sto.ele.*	*Sto.pil.*	*Sto.pul.*	*Sto.tra.*	*Sto.sp.*
182.						81			28					0			0	23		0		61				
183.		6						0	6	0		0	0	0	0		0	3	2	0		0		0	0	
			18						0							17					0		0			
						19			7					0			5	8		0		7				
184.						30			23					0			0	22		0		24				
185.	+											+														
186.					106				25									0				0				
187.	121							0	0			0		0	0		0	0		0		0				

Ref. No.	Reference/Source	Record Type	Data Type	Annotation	Ocean Region	Locale
	palangres de superficie en el Mediterráneo occidental y Canarias. 8th Simposio sobre el Margen Ibérico Atlántico, Malaga, Spain.	tech report	survey	3 CC turtles, none with coronuloid barnacles	Atlantic	Spain, Canary Islands
188.	Scaravelli, D. (1998a). Segnalazioni faunistiche. 19. *Platylepas hexastylos* (Fabricius, 1798) (Crustacea, Thoracica, Balanidae). *Quaderno di Studi e Notizie di Storia Naturale della Romagna* **9,** 75.	peer	record	1 CC turtle with *Che.tes.* and *Pla.hex.*	Mediter.	Adriatic Sea, Italy
189.	Scaravelli, D. (1998b). Segnalazioni faunistiche. 29. Stomatolepas elegans (O.G. Costa, 1838) (Crustacea, Thoracica, Balanidae). *Quaderno di Studi e Notizie di Storia Naturale della Romagna* **10,** 77-78.	peer	record	1 CC turtle with *Che.tes.* and *Sto.ele.*	Mediter.	Adriatic Sea, Italy
190.	Scaravelli, D., Affronte, M., & Costa, F. (2003). Analysis of epibiont presence on *Caretta caretta* from Adriatic Sea. Proceedings of the First Mediterranean Conference on Marine Turtles. D. Margaritoulis and A. Demetropoulos. Rome, Italy, Instituto Centrale per la Ricerca scientifica e tecnologica Applicata al Mare/International Union for Conservation of Nature, Cyprus, 221-225.	tech report	survey	30 CC turtles, 22 with *Che.tes* ., 23 with *Pla.hex* ., and 7 with *Sto.ele.*	Mediter.	Adriatic Sea, Italy
191.	Schärer, M. T. (2001). A survey of the epibiota of hawksbill sea turtles (*Eretmochelys imbricata*) of Mona Island, Puerto Rico, University of Puerto Rico, Mayagüez. M.S. thesis	tech report	survey	105 EI turtles, 4.76% with *Che.car* ., 1.90% with *Che.tes* ., and 77.14% with *Pla.dec* . (scored herein as *Pla.sp* . after Schärer 2003)	Carib.	Puerto Rico, Mona Island
192.	Schärer, M. T. (2003). A survey of the epibiota of *Eretmochelys imbricata* (Testudines, Cheloniidae) of Mona Island, Puerto Rico. *Revista de Biología Tropical* **51(Suppl. 4),** 87-90.	peer	record	Data from Schärer, 2001: 105 EI turtles, some with *Che.car.* and *Pla.sp.*	Carib.	Puerto Rico, Mona Island
193.	Schwartz, F.J. (1960). The barnacle *Platylepas hexastylos* encrusting a green turtle, *Chelonia mydas mydas,* from Chincoteague Bay, Maryland. *Chesapeake Science* **1,** 116-117.	peer	record	1 CM turtle with *Pla.hex.*	Atlantic	USA, Chincoteague Bay, MD

Ref. No.	total turtles analyzed by species							counts of turtles hosting corresponding species of barnacles																		
	CC	CM	DC	EI	LK	LO	ND	*Che.car.*	*Che.tes.*	*Che.ram.*	*Che.sp.*	*Chl.che.*	*Cal.bjr.*	*Cyl.dar.*	*Cyl.sin.*	*Pla.cor.*	*Pla.dec.*	*Pla.hex.*	*Pla.sp.*	*Ste.mur.*	*Sto.drm.*	*Sto.ele.*	*Sto.pil.*	*Sto.pul.*	*Sto.tra.*	*Sto.sp.*
	3							0	0			0		0	0		0	0		0		0				
188.	1							0	1			0		0	0		0	1		0		0				
189.	1							0	1			0		0	0		0	0		0		1				
190.	33							0	22			0		0	0		0	23		0		7				
191.				105				5	2			0			0				81	0		0				
192.				+				+	0			0			0				+	0		0				
193.		1						0	0	0		0	0	0	0		0	1		0		0		0	0	

Ref. No.	Reference/Source	Record Type	Data Type	AnnotatIon	Ocean Region	Locale
194.	Shahdadi, A., Sari, A., & Naderloo, R. (2014). A checklist of the barnacles (Crustacea: Cirripedia: Thoracica) of the Persian Gulf and Gulf of Oman with nine new records. *Zootaxa* **3784,** 201-223.	peer	list	CM turtles with *Che.car., Che.tes.,* and *Pla.hex.*	Indian	Arabian Sea, Persian Gulf & Gulf of Oman
195.	Smaldon, G. & Lyster, I.H.I. (1976). *Stomatolepas elegans* (Costa, 1840) (Cirripedia): new records and notes. *Crustaceana* **30,** 317-318.	peer	record	1 DC turtle with *Sto.ele.* (scored herein as *Sto.drm* .)	Atlantic	Scotland
				1 DC turtle with *Sto.ele* . (scored herein as *Sto.drm* .)	Indo-Pacific	Maylaysia, Kuala Lumpur
196.	Sosa-Cornejo, I., Montaño-Valdez, D.I., Bucio-Pacheco, M., Enciso-Saracho, F., Sanchez-Zazueta, J.G., & Fierros-Pérez, E. (2012). *Lepidochelys olivacea* (Eschscholtz, 1829) nesting in Ceuta Beach, Sinaloa, Mexico. *Journal of Agricultural Science and Technology B* **2,** 1190-1194.	peer	survey	104 LO turtles, 43 with epibionts, *Che.tes., Sto.prae* . (syn. *Sto.ele* .), and *Ste.mur.*	Pacific	Mexico, Sinaloa, Ceuta Beach
197.	Stamper, M.A., Harms, C., Epperly, S.P., Braun-McNeill, J., Avens, L., & Stoskopf, M.K. (2005). Relationship between barnacle epibiotic load and hematologic parameters in loggerhead sea turtles (*Caretta caretta*), a comparison between migratory and residential animals Pamlico Sound, North Carolina. *Journal of Zoo and Wildlife Medicine* **36,** 635-641.	peer	survey	33 CC turtles, 15 migratory and 18 resident, all with *Che.tes.*	Atlantic	USA, NC
198.	Stubbings, H.G. (1965). West African Cirripedia in the collections of the Institut Francais d'Afrique Noire, Dakar, Senegal. *Bulletin de Institut Francais d'Afrique Noire, series A* **27,** 876-907.	peer	list	CC turtles with *Che.tes., Pla.hex.* and *Sto.ele* .; CM turtles with *Che.tes.* and *Pla.hex.*	Atlantic	Africa, Senegal
199.	Sundararaj, B. (1927). Littoral fauna of Krusadai Island in the Gulf of Mannar. Cirripedia (Barnacles). *Bulletin of the Madras Government Museum, Natural History Section* **1,** 111-115.	peer	record	Unspecified turtles with *Che.tes.*	Indian	Laccadive Sea, Krusadai Island
200.	Tachikawa, H. (1995). Notes on three species of stalked barnacles found from a turtle barnacle on the carapace of a green turtle, *Chelonia mydas* . *Nanki Seibutu* **37,** 67-68.	peer	record	1 CM turtle with *Che.tes.*	Pacific	Japan

Ref. No.	total turtles analyzed by species							counts of turtles hosting corresponding species of barnacles																		
	CC	CM	DC	EI	LK	LO	ND	*Che.car.*	*Che.tes.*	*Che.ram.*	*Che.sp.*	*Chl.che.*	*Cal.bjr.*	*Cyl.dar.*	*Cyl.sin.*	*Pla.cor.*	*Pla.dec.*	*Pla.hex.*	*Pla.sp.*	*Ste.mur.*	*Sto.drm.*	*Sto.ele.*	*Sto.pil.*	*Sto.pul.*	*Sto.tra.*	*Sto.sp.*
194.		+						+	+	0		0	0	0	0		0	+		0		0		0	0	
195.			1						0							0					1		0			
			1						0							0					1		0			
196.						+			+					0			0	0		+		+				
197.	33							0	33			0		0	0		0	0		0		0				
198.	+							0	+			0		0	0		0	+		0		+				
		+						0	+	0		0	0	0	0		0	+		0		0		0	0	
199.									+																	
200.		1						0	1	0		0	0	0	0		0	0		0		0		0	0	

Ref. No.	Reference/Source	Record Type	Data Type	Annotation	Ocean Region	Locale
201.	Thurston, E. (1895). Rámésvaram Island and fauna of the Gulf of Manaar. *Madras Government Museum Bulletin* **3,** 77-138.	peer	list	CM turtles with *Che.tes.*	Indian	Laccadive Sea, Sri Lanka
202.	Torres-Pratts, H., Schärer, M.T., & Schizas, N.V. (2009). Genetic diversity of *Chelonibia caretta,* commensal barnacles of the endangered hawksbill sea turtle *Eretmochelys imbricata* from the Caribbean (Puerto Rico). *Journal of the Marine Biological Association of the United Kingdom* **89,** 719–725.	peer	survey	10 EI turtles (3 female, 6 male, 1 juvenile) with *Che.car.; Che.tes* . was taken from an 11th EI turtle	Carib.	Puerto Rico
203.	Tucker, A.D. (1988). A summary of leatherback turtle, *Dermochelys coriacea,* nesting at Culebra, Puerto Rico, from 1984-1987 with management recommendations. Washington, DC, Unpublished report to the U.S. Department of Interior, Fish and Wildlife Service.	tech report	record	DC turtles with *Sto.drm* . and *Pla.hex* . (scored herein as *Pla.cor.*)	Carib.	Puerto Rico
204.	Utinomi, H. (1949). Studies on the cirripedian fauna of Japan VI. Cirripeds from Kyusyu and Ryukyu Islands. *Publications of the Seto Marine Biological Laboratory* **1,** 19-37 + figs. 1-6.	peer	list	CC turtles with *Che.tes.*	Indian	Arabian Sea, Iran
205.	Utinomi, H. (1969). Cirripedia of the Iranian Gulf. *Videnskabelige Meddelelser fra dansk naturhistorisk Forening København* **132,** 79-94.	peer	list	CM turtles with *Che.tes.* and *Che.car.*	Pacific	Japan
206.	Utinomi, H. (1970). Studies on the cirripedian fauna of Japan. IX. Distributional survey of thoracic cirripeds in the southeastern part of the Japan Sea. *Publications of the Seto Marine Biological Laboratory* 1**7,** 1-366.	peer	list	CC turtles with *Che.tes.* and *Pla.hex* .; DC turtles with *Sto.ele* . (scored herein as *Sto.drm* .)	Pacific	Japan, Ryukyu Islands
207.	Vallini, C., Rubini, S. Tarricone, L., Mazziotti, C., & Gaspari, S. (2011). Unusual stranding of live, small, debilitated loggerhead turtles along the northwestern Adriatic Coast. *Marine Turtle Newsletter* **131,** 25-28.	peer	record	13 CC turtles, some with *Che.car* . and *Che.tes.* , all with *Pla.hex.*	Mediter.	Adriatic Sea, Italy

Ref. No.	total turtles analyzed by species							counts of turtles hosting corresponding species of barnacles																		
	CC	CM	DC	EI	LK	LO	ND	*Che.car.*	*Che.tes.*	*Che.ram.*	*Che.sp.*	*Chl.che.*	*Cal.bjr.*	*Cyl.dar.*	*Cyl.sin.*	*Pla.cor.*	*Pla.dec.*	*Pla.hex.*	*Pla.sp.*	*Ste.mur.*	*Sto.drm.*	*Sto.ele.*	*Sto.pil.*	*Sto.pul.*	*Sto.tra.*	*Sto.sp.*
201.		+						0	+	0		0	0	0	0		0	0		0		0		0	0	
202.				11				10	1			0			0		0	0		0		0				
203.			+													+					+					
204.	+							0	+			0		0	0		0	0		0		0				
205.		+						+	+	0		0	0	0	0		0	0		0		0		0	0	
206.	+							0	+			0		0	0		0	+		0		0				
			+						0							+					+		0			
207.	13							+	+			0		0	0		0	13		0		0				

Ref. No.	Reference/Source	Record Type	Data Type	Annotation	Ocean Region	Locale
208.	van Gompel, J. (1989). Eerste waarneming aan de Belgische kust van de lederschildpad *Dermochelys coriacea* (Linnaeus, 1758). *De Strandvlo* **9,** 102.	peer	record	DC turtles with *Sto.drm.*	Atlantic	North Sea, Belgium
209.	Van Syoc, R.J. (2009). Part 23. Barnacles. Marine Biodiversity of Costa Rica, Central America. I. S. Wehrtmann and J. Cortés. Netherlands, Springer, 283-289.	peer	list	Unspecified turtles with *Che.car., Ch.man. Ch.pat.* (the latter 2 syn. for *Che.tes.*), *Cyl.dar., Pla.dec., Pla.hex., Ste.mur., Sto.ele.,* and *Sto.trn.*	Carib.	Costa Rica
				hosts unspecified for *Che.tes.* and *Pla.hex.*	Pacific	Costa Rica
210.	Velasco-Charpentier, C., Pizarro-Mora, F., Estrades, A., & Veléz-Rubio, G.M. (2016). Epibiontes en juveniles de tortugas carey *Eretmochelys imbricata* varadas en la costa del Departamento de Rocha, Uruguay. *Revista de Biología Marina y Oceanografía* **51,** 449-453.	peer	survey	4 EI turtles with *Che.tes.* and *Pla.hex.*	Atlantic	Uruguay
211.	Vivaldo, S.G., Sarabia, D.O., Salazar, C.P., Hernández, A.G., & Lezama, J.R. (2006). Identification of parasites and epibionts in the Olive Ridley Turtle (*Lepidochelys olivacea*) that arrived to the beaches of Michoacan and Oaxaca, Mexico. *Veterinaria México* **37,** 431-440.	peer	record	LO turtles with *Che.tes., Pla.hex.* and *Sto.ele.*	Pacific	Mexico, Michoacán & Oaxaca
212.	Wagh, A.B. & Bal, D.V. (1974). Observations on the systematics of sessile barnacles from the west coast of India. *Journal of the Bombay Natural History Society* **71,** 109-123.	peer	record	1 E.i turtle with *Che.tes.*	Indian	India, Bombay
213.	Walker, G. (1978). A cytological study of the cement apparatus of the barnacle, *Chelonibia testudinaria* Linnaeus, an epizoite on turtles. *Bulletin of Marine Science* **28,** 205-209.	peer	record	1 LK turtle with *Che.tes.*	Atlantic	USA, NC, Beaufort

Ref. No.	total turtles analyzed by species							counts of turtles hosting corresponding species of barnacles																		
	**CC**	**CM**	**DC**	**EI**	**LK**	**LO**	**ND**	***Che.car.***	***Che.tes.***	***Che.ram.***	***Che.sp.***	***Chl.che.***	***Cal.bjr.***	***Cyl.dar.***	***Cyl.sin.***	***Pla.cor.***	***Pla.dec.***	***Pla.hex.***	***Pla.sp.***	***Ste.mur.***	***Sto.drm.***	***Sto.ele.***	***Sto.pil.***	***Sto.pul.***	***Sto.tra.***	***Sto.sp.***
208.			+						0							0					+		0			
209.								+	+					+			+	+		+		+			+	
									+								+									
210.				4				0	2			0			0		0	4		0		0				
211.						+			+					0			0	+		0		+				
212.				1				0	1			0			0		0	0		0		0				
213.					1				1											0		0				

Ref. No.	Reference/Source	Record Type	Data Type	Annotation	Ocean Region	Locale
214.	Webber, W.R., Fenwick, G.D., Bradford-Grieve, J.M., Eagar, S.G., Buckeridge, J.S., et al. (2011). Phylum Arthropoda Subphylum Crustacea: shrimps, crabs, lobsters, barnacles, slaters, and kin. New Zealand Inventory of Biodiversity: . D. P. Gordon. Christchurch, New Zealand, Canterbury University Press. Vol. 2: Animalia: Chaetognatha, Ecdysozoa, Ichnofossils: 98-232.	peer	list	Unspecified hosts for *Pla.hex.* and *Sto.ele.*	Pacific	New Zealand
215.	Wells, H.A. (1966). Barnacles of the northeastern Gulf of Mexico. *Quarterly Journal of the Florida Academy of Sciences* **29,** 81-95.	peer	list	CC turtles with *Che.car., Che.tes., Pla.hex.,* and *Sto.prae* . (syn. *Sto.ele* .)	Gulf of Mexico	USA, FL, Franklin Co.
216.	Weltner, W. (1897). Verzeichnis der bisher beschriebene recenten Cirripedienarten. Mit Angabe der im Berliner Museum vorhandenen species und ihrer Fundorte. *Archiv für Naturgeschichte* **63,** 227-280.	peer	list	From turtles: *Che.car. Che.tes., Pla.hex., Pla.dec.,* and *Ste.mur.*	?	
					Carib.	Venezuela
217.	Weltner, W. (1899). Ergebnisse einer Reise nach dem Pacific (Schauinsland 1896-1897), Cirripedien. *Zoologische Jahrbucher* **12,** 441-447.	peer	record	Unspecified host for *Che.tes.*	Pacific	Hawaii, Pearl Harbor
218.	Weltner, W. (1910). Cirripedien von Ostafrika. Reise in Ostafrika in den Jahren 1903-1905, mit Mitteln der Hermann und Elise geb. Heckmann Wentzel-Stiftung ausgefürht. v. A. Voeltzkow. Stuttgart, Germany, E. Schweizerbart'sche Verlagsbuchhandlung, Nägele & Dr. Sproesser. Wissenschaftliche Ergebnisse. Band II. Systematische Arbeiten. Heft IV-V, 527-528.	peer	record	CM turtles with *Ch.car* and *Che.tes.*	Indian	Mozambique Channel, Europa Island
219.	Wirtz, P., Araújo, R., & Southward, A.J. (2006). Cirripedia of Madeira. *Helgoland Marine Research* **60,** 207-212.	peer	list	CC turtles with *Che.car.*	Atlantic	Portugal
220.	Witzell, W.N. (1983). Synopsis of biological data on the hawksbill turtle, *Eretmochelys imbricata* (Linnaeus, 1766). FAO Fisheries Synopsis. Rome, Food and agriculture organization of the United Nations. No. 137, 1-78.	tech report	record	EI turtles with *Che.car., Che.tes., Chl.che. (syn. Tub.che.), Pla.dec., Pla.hex.,* and *Ste.mur.*	global	

Ref. No.	total turtles analyzed by species							counts of turtles hosting corresponding species of barnacles																		
	CC	CM	DC	EI	LK	LO	ND	*Che.car.*	*Che.tes.*	*Che.ram.*	*Che.sp.*	*Chl.che.*	*Cal.bjr.*	*Cyl.dar.*	*Cyl.sin.*	*Pla.cor.*	*Pla.dec.*	*Pla.hex.*	*Pla.sp.*	*Ste.mur.*	*Sto.drm.*	*Sto.ele.*	*Sto.pil.*	*Sto.pul.*	*Sto.tra.*	*Sto.sp.*
214.																		+				+				
215.	+							+	+			0		0	0		0	+		0		+				
216.									+								+	+		+						
								+																		
217.									+																	
218.		+						+	+	0		0	0	0	0		0	0		0		0		0	0	
219.	+							+	0			0		0	0		0	0		0		0				
220.				+				+	+			+			0		+	+		+		0				

Ref. No.	Reference/Source	Record Type	Data Type	Annotation	Ocean Region	Locale
221.	Young, P.S. (1991). The Superfamily Coronuloidea Leach (Cirripedia, Balanomorpha) from the Brazilian coast, with redescription of *Stomatolepas* species. *Crustaceana* **61,** 190-212.	peer	list	CC turtles with *Che.car.* ; CM turtles with *Che.tes., Pla.hex., Sto.ele., and Sto.trn* .; unspecified turtles with *Pla.dec.*	Atlantic	Brazil
222.	Zaghdoudi-Allan, N.A., Roberts, M.A. Frick, M.G., Zardus, J.D. & Andersson, M. (2014). Analysis of population genetic structure in the embedding turtle barnacle *Stephanolepas muricata* . 34th International Sea Turtle Symposium. New Orleans, LA.	tech report	record	Barnacles from a variety of localities and unspecified host turtles	Pacific<br>Pacific<br>Pacific<br>Pacific<br>Atlantic<br>Mediter.	Mexico, Baja<br>Costa Rica, Golfo Dulce<br>Australia<br>Japan<br>USA, FL, Palm Beach<br>Tunisia
223.	Zann, L.P. & Harker, B.M. (1978). Egg production of the barnacles *Platylepas ophiophilus* Lanchester, *Platylepas hexastylos* (O. Fabricius), *Octolasmis warwickii* Gray and *Lepas anatifera* Linnaeus. *Crustaceana* **35,** 206-214.	peer	record	CM turtles with *Pla.hex.*	Pacific	Australia, Magnetic Island, Townsville
224.	Zardus, J.D. & Balazs, G.H. (2007). Two previously unreported barnacles commensal with the green sea turtle, *Chelonia mydas* (Linnaeus, 1758), in Hawaii and a comparison of their attachment modes. *Crustaceana* **80,** 1303-1315.	peer	record	CM turtles with *Pla.dec.* (misidentified, scored herein as *Cyl.sin* .) and *Sto.ele* . (unknown sp., scored herein as *Sto.sp.*).	Pacific	Hawaii
225.	Zardus, J.D. & Hadfield, M. G. (2004). Larval development and complemental males in *Chelonibia testudinaria,* a barnacle commensal with sea turtles. *Journal of Crustacean Biology* **24,** 409-421.	peer	record	CM turtles with *Che.tes.*	Pacific	Hawaii
226.	Zardus, J.D., Lake, D.T., Frick, M.G., & Rawson, P.D. (2014). Deconstructing an assemblage of 'turtle' barnacles, species assignments and fickle fidelity in *Chelonibia. Marine Biology* **161,** 45-59.	peer	record	CC turtles with *Che.car.* and *Che.tes* .; CM turtles with *Che.tes* .; EI turtles with *Che.car* . and *Che.tes* .; LK turtles with *Che.tes* .; and ND turtles with *Che.tes.*	global	

Ref. No.	total turtles analyzed by species							counts of turtles hosting corresponding species of barnacles																		
	CC	CM	DC	EI	LK	LO	ND	*Che.car.*	*Che.tes.*	*Che.ram.*	*Che.sp.*	*Chl.che.*	*Cal.bjr.*	*Cyl.dar.*	*Cyl.sin.*	*Pla.cor.*	*Pla.dec.*	*Pla.hex.*	*Pla.sp.*	*Ste.mur.*	*Sto.drm.*	*Sto.ele.*	*Sto.pil.*	*Sto.pul.*	*Sto.tra.*	*Sto.sp.*
221.	+							+	0			0		0	0		0	0		0		0				
		+						0	+	0		0	0	0	0		0	+		0		+		0	+	
																	+									
222.								0	0	0		0	0	0	0		0	0		+		0		0	0	
																				+						
								0	0	0		0	0	0	0		0	0		+		0		0	0	
								0	0			0		0	0		0	0		+		0				
																				+						
																				+						
223.		+						0	0	0		0	0	0	0		0	+		0		0		0	0	
224.		+						0	0	0		0	0		+		0	0		0		0				+
225.		+						0	+	0		0	0	0	0		0	0		0		0		0	0	
226.	+							+	+			0		0	0		0	0		0		0				
		+						0	+	0		0	0	0	0		0	0		0		0		0	0	
				+				+	+			0			0		0	0		0		0				
					+				+									0				0				
							+		+			0						0				0				

Ref. No.	Reference/Source	Record Type	Data Type	Annotation	Ocean Region	Locale
227.	Zullo, V.A. (1991). Zoogeography of the Shallow-Water Cirriped Fauna of the Galápagos Islands and Adjacent Regions in the Tropical Eastern Pacific. Galápagos Marine Invertebrates, Taxonomy, Biogeography, and Evolution in Darwin's Islands. M. J. James. New York, NY, Plenum Press, 173-192.	peer	list	CM and EI turtles and *Che.tes., Cyl.dar., Pla.dec.,* and *Ste.mur* . barnacles, but host/barnacle matches not given.	Pacific	Galapagos Islands, Ecuador
228.	Zullo, V.A. & Bleakney, J. S. (1966). The cirriped *Stomatolepas elegans* (Costa) on leatherback turtles from Nova Scotian waters. *Canadian Field-Naturalist* **80,** 162-165.	peer	record	2 DC turtles with *Sto.ele* . (scored herein as *Sto.drm* .)	Atlantic	Canada, Nova Scotia
229.	Zullo, V.A., Lang, W.H. (1978). Order Cirripedia. Annotated Checklist of the Biota of the Coastal Zone of South Carolina. R. G. Zingmark. Columbia, SC, University of South Carolina, 158-160.	peer	list	CC and CM turtles with Che.tes.; unspecified turtles with Pla.hex., and Sto.ele.	Atlantic	USA, SC
230.	Zardus collection	collection	record	Collector I-Jiunn Cheng	Indo-Pacific	Papua New Guinea
231.	Zardus collection	collection	record	Collector John Zardus	Atlantic	Trinidad
232.	Zardus collection	collection	record	Collector Al Segars	Atlantic	USA, FL
233	Zardus collection	collection	record	Collector Mike Frick	Atlantic	USA, FL
234.	Zardus collection	collection	record	Collector Tom Pitchford	Atlantic	USA, FL
235.	Zardus collection	collection	record	Collector Mike Frick	Atlantic	USA, MA
236.	Zardus collection	collection	record	Collector Joanne Braun-McNeill	Atlantic	USA, NC
237.	Zardus collection	collection	record	Collector Joanne Braun-McNeill	Atlantic	USA, NC
238.	Zardus collection	collection	record	Collector DuBose Griffin	Atlantic	USA, SC
239.	Zardus collection	collection	record	Collector Kelly Sloan	Atlantic	USA, SC
240.	Zardus collection	collection	record	Collector Kelly Sloan	Atlantic	USA, SC
241.	Zardus collection	collection	record	Collector Kelly Sloan	Atlantic	USA, SC

Ref. No.	total turtles analyzed by species							counts of turtles hosting corresponding species of barnacles																		
	CC	CM	DC	EI	LK	LO	ND	*Che.car.*	*Che.tes.*	*Che.ram.*	*Che.sp.*	*Chl.che.*	*Cal.bjr.*	*Cyl.dar.*	*Cyl.sin.*	*Pla.cor.*	*Pla.dec.*	*Pla.hex.*	*Pla.sp.*	*Ste.mur.*	*Sto.drm.*	*Sto.ele.*	*Sto.pil.*	*Sto.pul.*	*Sto.tra.*	*Sto.sp.*
227.		+		+					+					+			+			+						
228.			2						0							0					2		0			
229.	+							0	+			0		0	0		0	0		0		0				
		+						0	+	0		0	0	0	0		0	0		0		0		0	0	
																		+				+				
230.			1													1							0			
231.			2													2					1					
232.	2																					2				
233				1																		1				
234.	5																					5				
235.			1																		1					
236.	8																	8								
237.		2																2								
238.	3							1														2				
239.	8																	8		1		5				
240.					1											1					1					
241.			1																			1				

Ref. No.	Reference/Source	Record Type	Data Type	Annotation	Ocean Region	Locale
242.	Zardus collection	collection	record	Collector Kelly Thorvalsen	Atlantic	USA, SC
243.	Zardus collection	collection	record	Collector Mike Arendt.	Atlantic	USA, SCDNR
244.	Zardus collection	collection	record	Collector Christina Trapani	Atlantic	USA, VA
245.	Zardus collection	collection	record	Collector Christina Trapani	Atlantic	USA, VA
246.	Zardus collection	collection	record	Collector Christina Trapani	Atlantic	USA, VA
247.	Zardus collection	collection	record	Collector Peter Meylan	Carib.	Panama
248.	Zardus collection	collection	record	Collector Peter Meylan	Carib.	Panama
249.	Zardus collection	collection	record	Collector, Antonio Mignucci-Giannoni	Carib.	Puerto Rico
250.	Zardus collection	collection	record	Collector Phillipe Mayor	Carib.	St. Croix
251.	Zardus collection	collection	record	Collector Marina Fastigi	Carib.	West Indies
252.	Zardus collection	collection	record	Collector Marina Fastigi	Carib.	West Indies
253.	Zardus collection	collection	record	Collector Allen Foley	Gulf of Mexico	USA, FL, Monroe Co.
254.	Zardus collection	collection	record	Collector Ben Higgins	Gulf of Mexico	USA, TX
255.	Zardus collection	collection	record	Collector Ben Higgins	Gulf of Mexico	USA, TX
256.	Zardus collection	collection	record	Collector Jack Frazier	Indian	Seychelles, Aldabra Atoll
257.	Zardus collection	collection	record	Collector Jack Frazier	Indian	Africa, Kenya, Mtwapa Creek
258.	Zardus collection	collection	record	Collector George Huges	Indian	Africa, South Africa
259.	Zardus collection	collection	record	Collector George Huges	Indian	Africa, South Africa
260.	Zardus collection	collection	record	Collector George Huges	Indian	Africa, South Africa
261.	Zardus collection	collection	record	Collector Joshua Reece	Indian	Arabian Sea, Oman
262.	Zardus collection	collection	record	Collector Ian Bell	Indo-Pacific	Australia, Milman Island

Ref. No.	total turtles analyzed by species							counts of turtles hosting corresponding species of barnacles																	
	CC	CM	DC	EI	LK	LO	ND	*Che.car.*	*Che.tes.*	*Che.ram.*	*Che.sp.*	*Chl.che.*	*Cal.bjr.*	*Cyl.dar.*	*Cyl.sin.*	*Pla.cor.*	*Pla.dec.*	*Pla.hex.*	*Pla.sp.*	*Ste.mur.*	*Sto.drm.*	*Sto.ele.*	*Sto.pil.*	*Sto.pul.*	*Sto.tra.*	*Sto.sp.*
242.	1							1																		
243.	34																	34				6				
244.	6							1										5				2				
245.			1													1					1					
246.					1													1								
247.			2													2										
248.				2													1	1				1				
249.				1					0			0			0		1	0		0		0				
250.			16					16																		
251.			6													6					1					
252.				10				10									4	3								
253.		9						9																		
254.			1													1										
255.					1													1								
256.		2																							2	
257.				+								+														
258.	1																	1								
259.		1																1								
260.			3													3					2					
261.		2																2								
262.				4				4																		

Ref. No.	Reference/Source	Record Type	Data Type	Annotation	Ocean Region	Locale
263.	Zardus collection	collection	record	Collector Ian Bell	Indo-Pacific	Australia, Raine Island
264.	Zardus collection	collection	record	Collector John Zardus	Pacific	Australia
265.	Zardus collection	collection	record	Collector John Zardus	Pacific	Australia
266.	Zardus collection	collection	record	Collector John Zardus	Pacific	Australia
267.	Zardus collection	collection	record	Collector Ian Bell	Pacific	Australia, Coombe Reef
268.	Zardus collection	collection	record	Collector Kathy LaFauce	Pacific	Australia, Mon Repos
269.	Zardus collection	collection	record	Collector Kathy LaFauce	Pacific	Australia, Mon Repos
270.	Zardus collection	collection	record	Collector Ian Bell.	Pacific	Australia, Murdoch Island
271.	Zardus collection	collection	record	Collector George Balazs	Pacific	Bora Bora
272.	Zardus collection	collection	record	Collector George Balazs	Pacific	Central Pacific
273.	Zardus collection	collection	record	Collector Ellen Beaumont	Pacific	Galapagos Islands, Ecuador
274.	Zardus collection	collection	record	Collector Ellen Beaumont	Pacific	Galapagos Islands, Ecuador
275.	Zardus collection	collection	record	Collector George Balazs	Pacific	Hawaii
276.	Zardus collection	collection	record	Collector George Balazs	Pacific	Hawaii, Hawaii
277.	Zardus collection	collection	record	Collector George Balazs	Pacific	Hawaii, Hawaii
278.	Zardus collection	collection	record	Collector George Balazs	Pacific	Hawaii, Kauai
279.	Zardus collection	collection	record	Collector George Balazs	Pacific	Hawaii, Maui
280.	Zardus collection	collection	record	Collector George Balazs	Pacific	Hawaii, Molokai
281.	Zardus collection	collection	record	Collector George Balazs	Pacific	Hawaii, Oahu
282.	Zardus collection	collection	record	Collector Hiroyuki Sugunuma	Pacific	Japan
283.	Zardus collection	collection	record	Collector Hiroyuki Sugunuma	Pacific	Japan

Ref. No.	total turtles analyzed by species							counts of turtles hosting corresponding species of barnacles																		
	CC	CM	DC	EI	LK	LO	ND	*Che.car.*	*Che.tes.*	*Che.ram.*	*Che.sp.*	*Chl.che.*	*Cal.bjr.*	*Cyl.dar.*	*Cyl.sin.*	*Pla.cor.*	*Pla.dec.*	*Pla.hex.*	*Pla.sp.*	*Ste.mur.*	*Sto.drm.*	*Sto.ele.*	*Sto.pil.*	*Sto.pul.*	*Sto.tra.*	*Sto.sp.*
263.		6						2				6														
264.	2											+						2								
265.		9							+			+						9		4					9	
266.				1														1		+						
267.				3				3																		
268.	6							4			2											1				
269.		1										1														
270.				1				1																		
271.		1																1								
272.						1														1		1				
273.		3															3	1								
274.				1														1								
275.		+													+											
276.		11															11									
277.				2														2								
278.				2													2			1						
279.		8															1	1				8				
280.		1																								1
281.		28															11	10		1						6
282.	5																	5		1		1				
283.				1														1								

Ref. No.	Reference/Source	Record Type	Data Type	Annotation	Ocean Region	Locale
284.	Zardus collection	collection	record	Collector Hiroyuki Sugunuma	Pacific	Japan, Ogaswara Island
285.	Zardus collection	collection	record	Collector Hiroyuki Sugunuma	Pacific	Japan, Ogaswara Island
286.	Zardus collection	collection	record	Collector Ryota Hayashi	Pacific	Japan, Okinawa
287.	Zardus collection	collection	record	Collector Michael Frick	Pacific	Mexico, Baja
288.	Zardus collection	collection	record	Collector Michael Frick	Pacific	Mexico, Baja
289.	Zardus collection	collection	record	Collectors Caren Barcello & Hoyt Peckham	Pacific	Mexico, Baja

Ref. No.	total turtles analyzed by species							counts of turtles hosting corresponding species of barnacles																		
	**CC**	**CM**	**DC**	**EI**	**LK**	**LO**	**ND**	***Che.car.***	***Che.tes.***	***Che.ram.***	***Che.sp.***	***Chl.che.***	***Cal.bjr.***	***Cyl.dar.***	***Cyl.sin.***	***Pla.cor.***	***Pla.dec.***	***Pla.hex.***	***Pla.sp.***	***Ste.mur.***	***Sto.drm.***	***Sto.ele.***	***Sto.pil.***	***Sto.pul.***	***Sto.tra.***	***Sto.sp.***
284.	1																	1								
285.		129																129								
286.		2													+										2	
287.		5																		5						
288.			1																		1					
289.	10																			10						
